# RIME optimization with dynamic multi-dimensional random mechanism and Nelder–Mead simplex for photovoltaic parameter estimation

**DOI:** 10.1038/s41598-025-99105-6

**Published:** 2025-07-01

**Authors:** Yuanping Zheng, Fangjun Kuang, Ali Asghar Heidari, Lipei Yuan, Siyang Zhang, Huiling Chen

**Affiliations:** 1 Department of Computer Science and Artificial Intelligence, Wenzhou University, Wenzhou, 325035 China; 2 School of Information Engineering, Wenzhou Business College, Wenzhou, 325035 China; 3 School of Surveying and Geospatial Engineering, College of Engineering, University of Tehran, Tehran, Iran

**Keywords:** RIME optimization algorithm, Optimization, Photovoltaic parameter extraction, Dynamic multi-dimensional random mechanism, Nelder–Mead simplex, Engineering, Mathematics and computing, Computer science

## Abstract

Solar photovoltaic technology is efficient and clean, but extracting photovoltaic cell parameters is challenging due to various influencing factors. The rime optimization algorithm (RIME) is a recently proposed metaheuristic algorithm (MAs). This paper introduces the dynamic multi-dimensional random mechanism (DMRM) combined with the Nelder–Mead simplex (NMs) to propose an enhanced version of RIME, called DNMRIME. DMRM improves the convergence accuracy of RIME by random non-periodic convergence, and NMs accelerate convergence, enabling DNMRIME to escape local optima and perform better on hybrid and composite functions. To evaluate the performance of DNMRIME, a qualitative analysis and an ablation study were conducted on CEC 2017. To verify its effectiveness, DNMRIME was compared with 14 well-known MAs, including some champion algorithms, and the results of the Wilcoxon signed rank test showed that DNMRIME ranked first. To extract parameters on SDM, DDM, TDM, and PV, DNMRIME was applied, resulting in mean RMSE values of 9.8602188324E − 04, 9.8296993325E − 04, 9.8393451046E − 04, and 2.4250748704E − 03 respectively. Moreover, under varying temperature and irradiation conditions on three manufacturers (KC200GT, ST40, SM55), DNMRIME extracted parameters with simulation data matching the actual data. Therefore, unlike previous studies, this study proposes DMRM and DNMRIME, demonstrating the efficiency and practicality of DNMRIME and further highlighting potential value of DNMRIME in photovoltaic parameter extraction. The source code of DNMRIME is available at https://github.com/zyetpink/DNMRIME-Solar-Model-dataset.

## Introduction

In recent years, escalating environmental pollution has increasingly threatened human health and sustainable social development, sparking an urgent quest for clean and renewable energy sources to replace traditional, highly polluting ones^1,2^. Photovoltaic energy, as a clean, pollution-free, and renewable form of energy, not only holds economic advantages but also boasts broad application prospects, including daily power supply, electric vehicles, aerospace, and other fields^3,4^. Photovoltaic energy has become crucial for ditching traditional energy sources^5^. Furthermore, in numerous regions and countries, solar energy significantly promotes energy diversity and security^6^. Therefore, the rising global need for sustainable energy has made photovoltaic energy crucial^7^.

Owing to the influence of varying light intensity, temperature, and the non-uniform and intricate structure of photovoltaic cells^8^, the photovoltaic model exhibits nonlinear relationships and complex structures, resulting in transcendental equations^9,10^. Nonlinearity and precise estimation of unknown parameters pose a challenge in solar energy. Precise parameter extraction is vital for maximizing solar energy utilization. Analytical methods^11–13^, numerical methods^14–16^ and metaheuristic algorithms (MAs)^17^ are three commonly used approaches for determining the parameters or characteristics of photovoltaic systems. Analytical methods face challenges such as high computational costs and significant model dependencies when using mathematical models and computational techniques in various applications^18^.

Meanwhile, numerical techniques are constrained by the quality and limitations of experimental data^19^. Conversely, MAs demonstrate robustness and adaptability in estimating composite photovoltaic parameters, enabling them to flexibly address diverse systems and optimization objectives, improving accuracy and reliability^20^. Through its parallel processing and computational efficiency, it not only minimizes costs in time and resources but also quickly reaches the global optimum, surpassing the constraints of traditional methods and emerging as a highly effective optimization technique.

In recent years, MAs have emerged in various research fields by establishing mathematical models by simulating natural behaviors, demonstrating strong adaptability and flexibility^21^. Notable MAs include genetic algorithm (GA)^22^, particle swarm optimization (PSO)^23^, differential algorithm (DE)^24^, artificial bee colony (ABC)^25^, whale optimization algorithm (WOA)^26^, grey wolf optimization (GWO)^27^, Harris hawks optimization (HHO)^28^, hunger games search (HGS)^29^, teaching–learning based optimization (TLBO)^30^, colony predation algorithm (CPA)^31^, liver cancer algorithm (LCA)^32^, Runge Kutta optimizer (RUN)^33^, competitive swarm optimizer (CSO)^34^ and others. Recently proposed algorithms include, slime mould algorithm (SMA)^35,36^, educational competition optimizer (ECO)^37^, artemisinin optimization (AO)^38^, the weighted mean of vectors (INFO)^39^, fata morgana algorithm (FATA)^40^, rime optimization algorithm (RIME)^41^, polar lights optimization (PLO)^42^, parrot optimizer (PO)^43^, quantum-based avian navigation optimizer (QANA)^44^ and more.

Recent studies have highlighted the versatile applications of MAs and their enhanced versions across various fields^45^. These algorithms have proven to be particularly effective in addressing complex optimization problems. For example, the offline learning-enhanced CSO has been applied to efficiently solve nonlinear fixed-cost transportation problems^46^. At the same time, the reinforcement learning-improved HHO has been utilized for high-dimensional feature selection^47^. The enhanced ABC has also shown promising results in breast cancer image segmentation^48^. Using MAs to estimate photovoltaic parameters is the mainstream method^49^. Table 1 summarizes recent research on MAs for photovoltaic parameter estimation, including methods and targeted solar models. Many researchers have used (MAs) or enhanced versions for photovoltaic parameter estimation, and these significant contributions have provided valuable insights into the field. However, these studies lack in-depth performance analysis of the algorithms used and do not comprehensively evaluate photovoltaic parameter models and commercial model systems.

**Table 1:** Summary of MAs for photovoltaic parameter estimation.

Year	References	Main work and method	Solar model
2015	^50^	Used flower pollination algorithm (FPA)	SDM, DDM
2016	^51^	Proposed an adaptive NMs to improve artificial bee colony (ABC)	SDM, DMM, PV
2016	^52^	Applied moth-flame optimizer (MFO)	DDM, TDM
2017	^53^	Proposed a hybrid algorithm of ABC and FPA	SDM, DDM
2018	^54^	Improved PSO with adaptive mutation strategy	SDM, DDM, PV
2018	^55^	Proposed a new hybrid of ABC and TLBO	SDM, DDM, PV
2019	^56^	Improved interior search algorithm	SDM, DDM
2020	^57^	Proposed a hybrid of GWO and cuckoo search	SDM, DDM, PV
2020	^58^	Improved WOA with reflection principle	SDM
2020	^59^	Improved bat algorithm with Lévy flight	SDM, DDM
2020	^60^	Proposed a hybrid of TLBO and DE	SDM, DDM, PV
2020	^61^	Used manta ray foraging optimization	TDM
2021	^62^	Improved DE with population information and search direction	SDM, DDM, TDM, PV
2021	^63^	Proposed a new hybrid of MPA and SMA	TDM
2021	^64^	Improved TLBO by dividing into three phases based on scores	SDM, DDM, PV
2021	^65^	Improved SMA by local search capability	SDM, DDM, PV
2021	^66^	Improved MPA by population enhancement	SDM, DDM, PV
2022	^67^	Used circle search algorithm	TDM
2022	^68^	Improved WOA by information sharing strategy and NMs	SDM, DDM, TDM, PV
2022	^69^	Improved colony predation algorithm by opposition and level learning	SDM, DDM, PV
2022	^70^	Combined QANA and the Newton–Raphson method	SDM, DDM, PV
2023	^71^	Improved atomic search optimization by anti-sine–cosine strategy	SDM, DDM, TDM, PV
2023	^72^	Improved GWO by spiral updating and multiple learning backtracking	
2023	^73^	Improved elephant herding optimization by fast moving operator and elite strategy	PV
2023	^74^	Improved FPA by combining three new strategies	SDM, DDM, PV
2023	^75^	Improved DE by four strategies	SDM, DDM, PV
2024	^76^	Improved clonal selection algorithm using golden sine and dual-feedback strategies	SDM, DDM
2024	^77^	Improved exponential distribution optimization by opposition learning	SDM, DDM, TDM, PV

Lately, the domain of MAs has been consistently observing the sprouting of novel algorithms. Remarkably, the rime optimization algorithm (RIME), a novel approach grounded in physical phenomena^78^, was introduced in 2023 and quickly attracted widespread interest. This is due to its advantages: (1) Simple and intuitive working mechanism is easy to understand and implement; (2) It requires fewer parameters, reducing the need for algorithm adjustment; (3) RIME exhibits a relatively fast convergence speed when dealing with multimodal functions. Considering the notable advantages of the RIME, we have recognized its potential and attempted to apply it to practical photovoltaic parameter estimation.

RIME demonstrates strong exploration capabilities on unimodal functions, such as F2 and F3 on the CEC 2017 benchmark, where it effectively searches for the global optimum. Additionally, RIME can escape local optima on multimodal functions, as seen on CEC2017 functions F8, F9 and F10. However, when faced with more complex hybrid and composition functions, the performance of RIME is less satisfactory, often struggling to escape local optima and exhibiting deficiencies in convergence accuracy. This indicates that RIME still has room for improvement in balancing global exploration and local search in complex search spaces. Photovoltaic parameter extraction is a complex optimization problem where optimization algorithms need to escape local optima while also requiring precise local search capabilities to ensure rapid convergence to high-accuracy solutions during the search process. According to the "No Free Lunch Theorem" (NFL)^79^, there is no universal algorithm that performs optimally on all problems. Consequently, choosing the appropriate algorithm based on the problem is necessary. In the photovoltaic parameter extraction experiments, we found that the original RIME algorithm did not perform satisfactorily.

To better accelerate the convergence speed and improve the convergence accuracy of RIME, we propose an enhanced algorithm called DNMRIME in our research. This paper combines dynamic multi-dimensional random mechanism (DMRM) and NMs into a straightforward and effective RIME algorithm to better solve complex optimization issues like photovoltaic parameter extraction. In the Soft-rime search strategy of RIME, although the cosine function introduces a certain level of controlled volatility, providing some adjustment ability to the search process, this volatility follows a deterministic periodic variation. Specifically, we introduce DMRM, which uses uncertain perturbations and a non-periodic sine function to improve RIME’s convergence accuracy and local search capability. We also used the NMs to improve the local search ability and convergence speed. To validate the performance of the DNMRIME, we conducted extensive testing on the CEC 2017 and a series of simulation experiments in photovoltaic models. Through rigorous analysis of the experimental results, we found that DNMRIME demonstrated superior performance across multiple test cases. Compared to RIME and other relevant algorithms, DNMRIME exhibited higher convergence speeds and better parameter estimation accuracy.

Based on the above, the key differences of this study compared to previous research are as follows.

- The dual-mechanism improvement of RIME, where DMRM is proposed for the first time and combined with NMs to enhance RIME.
- This study conducts comprehensive validation and photovoltaic parameter extraction by evaluating DNMRIME on CEC 2017 and testing DNMRIME on SDM, DDM, TDM, KC200GT, ST40, and SM55.

Therefore, the contributions of this study are as follows:

- Proposal of DMRM: This study introduces DMRM, which enhances RIME’s ability to escape local optima and improves its convergence accuracy by incorporating a sine function and a sigmoid function.
- Integration of DMRM and NMs into RIME: By combining DMRM and NMs to enhance RIME, the proposed algorithm, DNMRIME, achieves a better balance between exploration and exploitation.
- Qualitative analysis of DNMRIME: A qualitative analysis of DNMRIME on the CEC 2017 benchmark is conducted to evaluate the convergence trend of the population and the dimensional change trajectories.
- Ablation study of DNMRIME: The ablation study of DNMRIME on CEC 2017 validates the effectiveness of DMRM and NMs, confirming their contributions to DNMRIME.
- Competitiveness analysis: This study compares DNMRIME with 14 well-known algorithms on CEC 2017, and the Wilcoxon signed-rank test confirms its competitive performance.
- Application in photovoltaic parameter extraction: The photovoltaic parameter extraction capability of DNMRIME is comprehensively evaluated using SDM, DDM, TDM, and PV, showing lower RMSE than existing algorithms.
- Performance under real-world conditions: Three commercial photovoltaic models (KC200GT, ST40, SM55) are used to assess DNMRIME under different temperature and illumination conditions, further validating its optimization performance.

The main structure of this paper is as follows: Section “Photovoltaic problem definition and equations”, the photovoltaic problem and its formulas are described. In “The proposed DNMRIME algorithm”, the DNMRIME algorithm is introduced. Section “Experimental results”, experiments are conducted on DNMRIME. Section “Discussion on the results” presents a discussion on DNMRIME. Section “Conclusions and future directions”, finally, prospects are outlined.

## Photovoltaic problem definition and equations

This section introduces modeling methods, equivalent circuit models, and relevant mathematical equations for photovoltaic energy problems. Establishing precise mathematical models is vital for analyzing system performance, optimizing designs, and developing effective control strategies in photovoltaic energy systems. We will describe four commonly used photovoltaic models: SDM, DDM, TDM, and PV.

### Solar cell model

#### Single diode model (SDM)

**Fig. 1:**
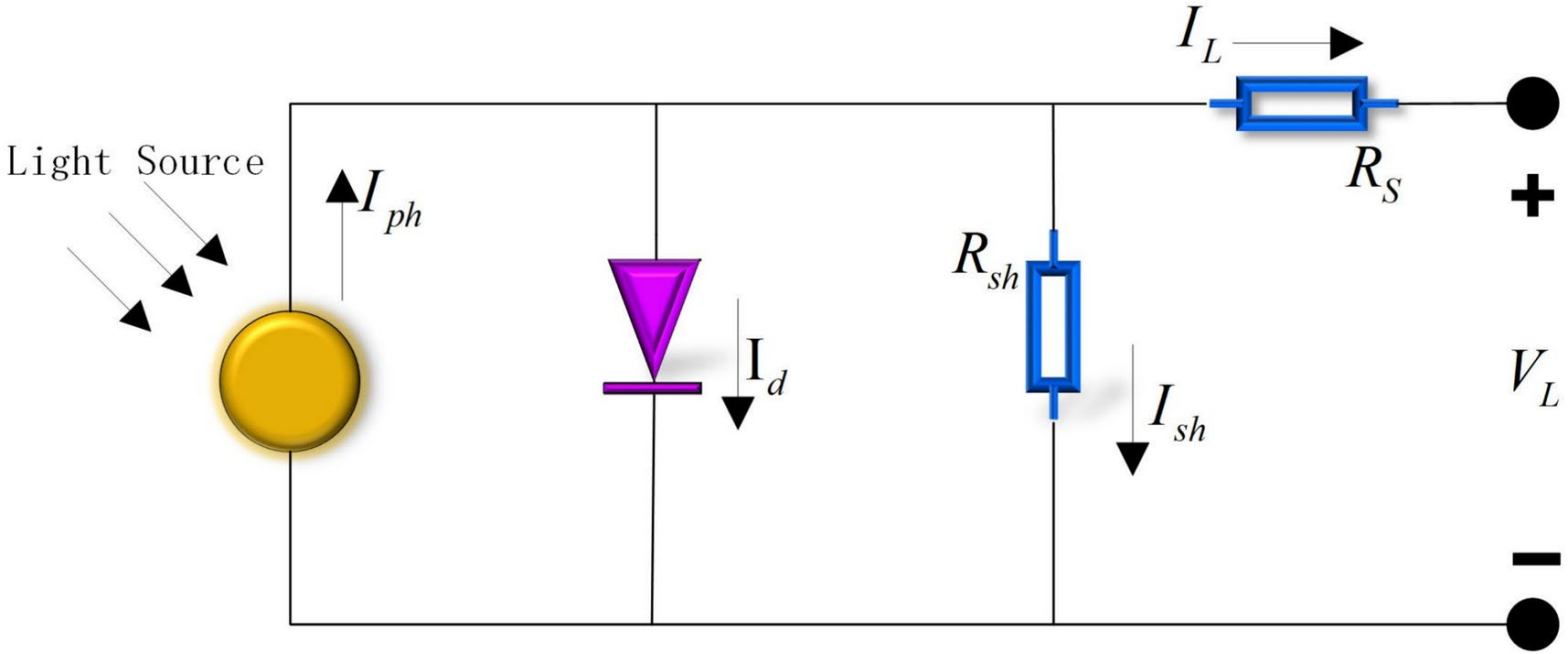
Equivalent circuit diagram for SDM.

The SDM is a widely employed simplified mathematical model utilized to elucidate the operational characteristics of photovoltaic cells. Grounded in both circuit theory and semiconductor physics principles, this model conceptualizes a PV cell as an equivalent circuit comprising a diode and a resistor. The schematic representation of the SDM’s equivalent circuit is depicted in Fig. 1. Equation (1) elucidates the method for calculating the output current of the photovoltaic cell in the SDM case.

1 $$I_{L} = I_{ph} - I_{sh} - I_{d}$$

where the current $I_{d}$ that through the diode can be calculated using Eq. (2), $I_{sh}$ stands for the current flowing through the parallel resistance $R_{sh}$, $R_{sh}$ can be derived from Eq. (3), $I_{ph}$ represents the current generated by light shining on the surface of the photovoltaic cell, $I_{L}$ is the final output current.

2 $$I_{d} = \left[ {\exp \left( {\frac{{V_{L} + R_{s} \times I_{L} }}{n \times k \times T} \times q} \right) - 1} \right] \times I_{sd}$$

3 $$I_{sh} = \frac{{V_{L} + R_{s} \times I_{L} }}{{R_{sh} }}$$

where $V_{L}$ is the final output voltage, $R_{s}$ is the series resistance used for voltage division, $n$ represents the ideality factor or coefficient of an ideal diode, $n$ is typically in the range (1, 2), $k$ is the Boltzmann constant approximately valued at 1.380649 × 10E − 23 J/K, the parameter $T$ denotes the Kelvin temperature of the photovoltaic cell.

Substituting Eqs. (2) and (3) into Eq. (1) yields Eq. (4).

4 $$I_{L} = I_{ph} - \left[ {\exp \left( {\frac{{V_{L} + R_{s} \times I_{L} }}{n \times k \times T} \times q} \right) - 1} \right] \times I_{sd} - \frac{{V_{L} + R_{s} \times I_{L} }}{{R_{sh} }}$$

In the SDM, there are five unknown parameters to be determined. These parameters include: $I_{ph} , \, I_{sd} , \, R_{s} , \, R_{sh} , \, n$. Determining the values of these parameters is crucial for accurately describing the behavior and performance of photovoltaic systems.

#### Double diode model (DDM)

**Fig. 2:**
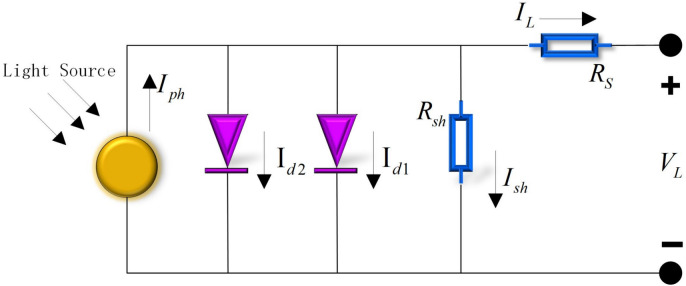
Equivalent circuit diagram of DDM.

DDM adds an extra parallel diode outside the components of SDM to better capture the intricate dynamics within the photovoltaic cell. The equivalent circuit diagram of DDM is illustrated in Fig. 2. On DDM, $I_{L}$ is calculated by Eq. (5).

5 $$I_{L} = I_{ph} - I_{sh} - I_{d1} - I_{d2}$$

where $I_{d1}$ is the current passing through the first diode, and $I_{d2}$ is the current passing through the second diode.

By substituting Eq. (4) into Eq. (5), Eq. (6) can be obtained.

6 $$I_{L} = I_{ph} - \frac{{V_{L} + R_{s} \times I_{L} }}{{R_{sh} }} - I_{sd1} \times \left[ {\exp \left( {\frac{{V_{L} + R_{s} \times I_{L} }}{{n_{1} \times k \times T}} \times q} \right) - 1} \right] - I_{sd2} \times \left[ {\exp \left( {\frac{{V_{L} + R_{s} \times I_{L} }}{{n_{2} \times k \times T}} \times q} \right) - 1} \right]$$

where $n_{1}$ and $n_{2}$ respectively represent the ideality factors or ideality coefficients of the two diodes.

On DDM, there are 7 unknown parameters to be determined. These parameters include: $I_{ph} , \, I_{sd1} , \, I_{sd2} , \, R_{s} , \, n_{1} , \, n_{2}$.

#### Three diode model (TDM)

**Fig. 3:**
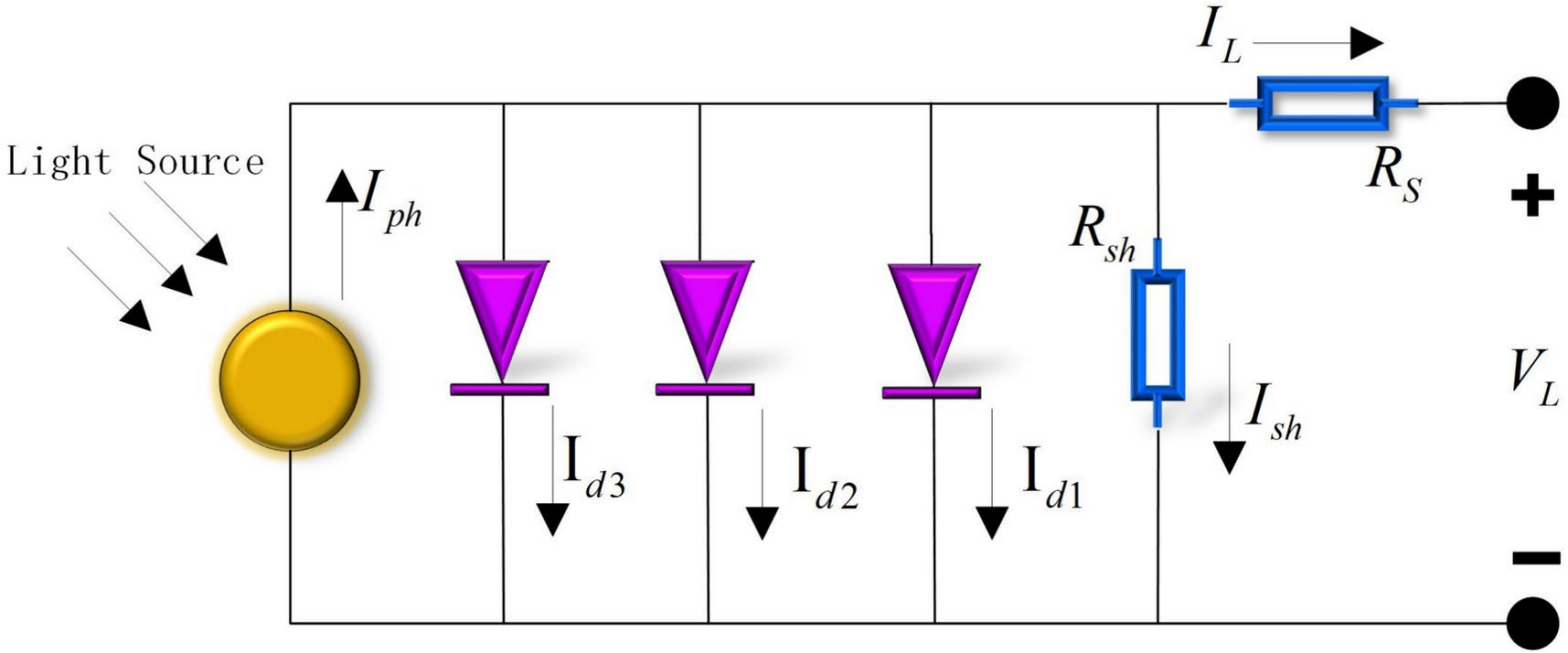
Equivalent circuit diagram of TDM.

TDM consists of three diodes in parallel, along with a parallel resistor and a series resistor, forming an equivalent circuit, as depicted in Fig. 3.

In the DDM, $I_{L}$ is calculated using Eq. (7).

7 $$I_{L} = I_{ph} - I_{sh} - I_{d1} - I_{d3}$$

where $I_{d1} , \, I_{d2} , \, I_{d3}$ represent the current through the first, second, and third diodes, respectively.

8 $$\begin{aligned} I_{L} & = I_{ph} - \frac{{V_{L} + R_{s} \times I_{L} }}{{R_{sh} }} - I_{sd1} \cdot \left[ {\exp \left( {\frac{{V_{L} + R_{s} \times I_{L} }}{{n_{1} \cdot k \cdot T}} \cdot q} \right) - 1} \right] \\ & \quad - I_{sd2} \times \left[ {\exp \left( {\frac{{V_{L} + R_{s} \times I_{L} }}{{n_{2} \times k \times T}} \times q} \right) - 1} \right] - I_{sd3} \times \left[ {\exp \left( {\frac{{V_{L} + R_{s} \times I_{L} }}{{n_{2} \times k \times T}} \times q} \right) - 1} \right] \\ \end{aligned}$$

where $n_{1} , \, n_{2} , \, n_{3}$ respectively represent the ideality factors or ideality coefficients of the three diodes.

In the TDM, there are nine unknown parameters to be determined. These parameters include: $I_{ph} , \, I_{sd1} , \, I_{sd2} , \, I_{d3} , \, R_{s} , \, R_{sh} , \, n_{1} , \, n_{2} , \, n_{3}$.

### PV module model

PV is a mathematical model used to describe the behavior of photovoltaic cells, typically composed of a photocurrent source and a series resistor, aiming to simulate the photovoltaic cell’s current characteristics accurately.

**Fig. 4:**
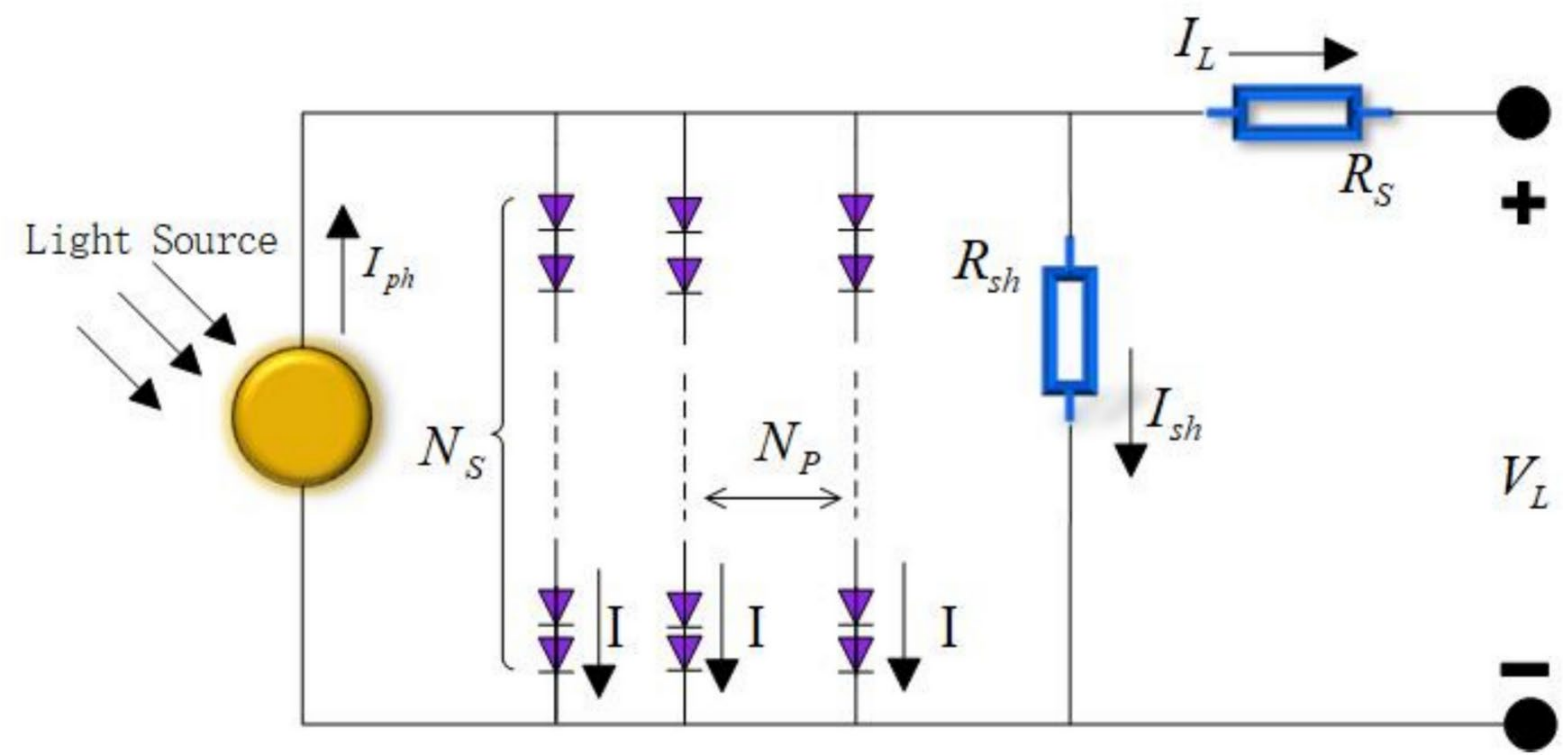
Equivalent circuit of PV.

The equivalent circuit diagram of PV is shown in Fig. 4.

Similarly, Eqs. (9) and (10) can also be derived accordingly.

9 $$I_{L} = I_{ph} N_{p} - I_{sd} \times N_{p} \times \left[ {\exp \left( {\frac{{\frac{{V_{L} }}{{N_{s} }} + N_{s} \times R_{s} \times \frac{{V_{L} }}{{N_{s} }}}}{{n_{1} \times k \times N_{s} \times T}} \times q} \right) - 1} \right] - \frac{{\frac{{V_{L} }}{{N_{s} }} + N_{s} \times R_{s} \times \frac{{I_{L} }}{{N_{p} }}}}{{\frac{{R_{sh} \times N_{s} }}{{N_{p} }}}}$$

10 $$\begin{gathered} I_{L} = I_{ph} \times N_{p} - I_{sd1} \times N_{p} \times \left[ {\exp \left( {\frac{{\frac{{V_{L} }}{{N_{s} }} + N_{s} \times R_{s} \times \frac{{V_{L} }}{{N_{s} }}}}{{n_{1} \times k \times N_{s} \times T}} \times q} \right) - 1} \right] \\ - I_{sd2} \times N_{p} \times \left[ {\exp \left( {\frac{{\frac{{V_{L} }}{{N_{s} }} + N_{s} \times R_{s} \times \frac{{V_{L} }}{{N_{s} }}}}{{n_{2} \times k \times N_{s} \times T}} \times q} \right) - 1} \right] - \frac{{\frac{{V_{L} }}{{N_{s} }} + N_{s} \times R_{s} \times \frac{{I_{L} }}{{N_{p} }}}}{{\frac{{R_{sh} \times N_{s} }}{{N_{p} }}}} \\ \end{gathered}$$

where $N_{s}$ represents the number of solar cells in series, $N_{P}$ represents the number of solar cells in parallel.

On PV, several unknown parameters need to be determined. These parameters include: $I_{ph}$, $I_{sd}$, $R_{sh}$, $n$.

### Objective function

In photovoltaic problems, the problem description can be represented by Eqs. (11)–(13).

It is vital to assess the deviation between actual and estimated values. The selection of Root Mean Square Error (RMSE) as an evaluation metric is justified by its sensitivity to outliers, interpretability of positive and negative errors^80^, and differentiability. The expression for RMSE is shown in Eq. (14).

11 $$f\left( {V_{L} ,I_{L} ,X} \right) = I_{ph} - I_{L} - I_{sd} \times \left[ {\exp \left( {\frac{{q \times \left( {V_{L} + R_{s} \times I_{L} } \right)}}{n \times k \times T}} \right) - 1} \right] - \frac{{V_{L} + R_{s} \times I_{L} }}{{R_{sh} }}$$

12 $$f\left( {V_{L} ,I_{L} ,X} \right) = I_{ph} - I_{L} - \frac{{V_{L} + R_{s} \times I_{L} }}{{R_{sh} }} - \sum\limits_{i = 1}^{2} {I_{sd,i} \times \left[ {\exp \left( {\frac{{q \times \left( {V_{L} + R_{s} \times I_{L} } \right)}}{{n_{i} \times k \times T}}} \right) - 1} \right]}$$

13 $$f\left( {V_{L} ,I_{L} ,X} \right) = I_{ph} - I_{L} - \frac{{V_{L} + R_{s} \times I_{L} }}{{R_{sh} }} - \sum\limits_{i = 1}^{3} {I_{sd,i} \times \left[ {\exp \left( {\frac{{q \times \left( {V_{L} + R_{s} \times I_{L} } \right)}}{{n_{i} \times k \times T}}} \right) - 1} \right]}$$

where $X$ represents the vector of parameters to be optimized.

14 $$RMSE\left( {X_{i} } \right) = \sqrt {\frac{1}{N}\sum\limits_{i = 1}^{N} {f^{2} \left( {V_{L} ,I_{L} ,X_{i} } \right)} }$$

where $N$ represents the number of samples, which refers to the quantity of data points.

The smaller the RMSE, the closer the predicted values are to the actual values. Therefore, the objective function to be minimized is shown in Eq. (15).

15 $$Minimize \, RMSE\left( {X_{i} } \right)$$

## The proposed DNMRIME algorithm

This section will introduce the original RIME, the NMs and the newly proposed dynamic multi-dimensional random mechanism. Furthermore, we will discuss the proposed improved algorithm, DNMRIME.

### The RIME algorithm

The principle of RIME is quite straightforward: rime is classified into soft and hard types based on its morphology. RIME has two main stages: soft-rime search strategy and Hard-rime puncture mechanism. A positive greedy absorption mechanism is also consistently utilized throughout RIME^78^.

#### Initialize rime particles

Initially, rime particles acquire their initial position according to Eq. (16).

16 $$R_{ij} = LB + r_{1} \times \left( {UB - LB} \right)$$

where $r_{1}$ is a random number in the range $\left[ {0,1} \right]$.

#### Soft-rime search strategy

When rime is in the soft state, a soft margin strategy is adopted, and the state of rime particles is updated using Eq. (17).

17 $$R_{{{\text{ij}}}}^{{{\text{new}}}} = R_{best,j} + r_{2} \times \cos \theta \times \beta \times \left( {h \times \left( {UB - LB} \right) + LB} \right),r_{3} < E,j = 1,2, \ldots ,dim$$

where $R_{ij}^{new}$ represents the new position of the updated rime particle, while $i$ and $j$ indicate the $j$-th dimension of the $i$-th rime particle, respectively, $R_{ij}^{best}$ represents the $j$-th dimension of the best rime agent within the RIME population. The parameter $r_{2}$ and $r_{3}$ is a random number in the range $\left[ { - 1,1} \right]$. $UB$ and $LB$ respectively represent the upper bound and lower bound of the search space.

18 $$\theta = \pi \times \frac{FEs}{{10 \times MaxFEs}}$$

The parameter $\theta$ is determined by Eq. (18), and as the iteration count rises, it grows larger. Consequently, the parameter $\cos \theta$ diminishes as the iteration count increases.

19 $$\beta = 1 - \left[ {\frac{w \times FEs}{{MaxFEs}}} \right]/w$$

The environmental factor $\beta$, varying with iterations as a random number in the range $\left[ {0,1} \right]$, is calculated using Eq. (19).

20 $$E = \sqrt {\left( {\frac{FEs}{{MaxFEs}}} \right)}$$

The parameter value $E$ is related to the current iteration count and the maximum iteration count in the range $\left[ {0,1} \right]$, as indicated in Eq. (20). Parameters $E$ and $r_{1}$ jointly determine, with a certain probability, whether a rime particle adopts the soft margin search strategy or the soft rime strategy.

#### Hard-rime puncture mechanism

When a rime particle is in a state of hard rime, Eq. (21) will be employed to update the position of the current fog rime particle.

21 $$R_{ij}^{new} = R_{best,j} ,r_{4} < F^{normr} \left( {S_{i} } \right)$$

where $R_{ij}^{new}$ represents the updated position of rime particles, $i$ and $j$ respectively denote the $j$-th dimension of the $i$-th RIME agent. The parameter $r_{4}$ is a random number in the range $\left[ {0,1} \right]$. $F^{normr} \left( {S_{i} } \right)$ represents the normalized fitness value of the $i$-th RIME agent.

### Nelder–Mead simplex

J. Nelder and R. Mead proposed the NMs in 1965^81^, a method capable of locally searching for minimum values in multi-dimensional space.

The NMs explore the solution space with a basic simplex, adapting its shape and position to find optimal values locally. According to objective function values, its shape is altered through operations like reflection, expansion, contraction, and shrinkage. The method is efficient and often used for unconstrained optimization, making it simple yet effective. The basic operations of the simplex in DNMRIME are as follows.

Step 1 Construction of the initial simplex. Firstly, a set of initial solutions is selected as the vertices of the simplex, with each point representing a parameter vector, which in this context corresponds to the RIME agent. Since the current dimensional space is $D$-dimensional, $D + 1$ vertices are chosen to construct an initial simplex of $D + 1$ dimensions.
Step 2 Sorting. Based on the fitness values of these $D + 1$ vertices, they are sorted in descending order of fitness and numbered accordingly, resulting in a sequence as shown in Eq. (22).
  22 $$f\left( {X_{1} } \right) \le f\left( {X_{2} } \right) \le ... \le f\left( {X_{D} } \right) \le f\left( {X_{D + 1} } \right)$$
Step 3 Calculating the centroid. The worst vertex $X_{D + 1}$ is removed, and the centroid (average position) of the remaining vertices is calculated to determine a new trial point $X_{C}$, as shown in Eq. (23).
  23 $$X_{C} = \frac{{\sum\nolimits_{i = 1}^{D} {X_{i} } }}{D}$$
Step 4 Reflection operation. Obtain the reflection point $X_{R}$ according to Eq. (24).
  24 $$X_{R} = X_{C} + \alpha \times \left( {X_{C} - X_{D + 1} } \right)$$
  where $\alpha$ is the reflection coefficient.
Step 5 Reflection point expansion operation. The expansion point $X_{E}$ is obtained according to Eq. (25).
  25 $$X_{E} = X_{C} + \beta \times \left( {X_{R} - X_{C} } \right)$$
  where $\beta$ is the expansion coefficient. If $f\left( {X_{E} } \right) \le f\left( {X_{R} } \right)$, then $X_{E}$ is used instead of $X_{D + 1}$ to construct a new simplex. Otherwise, if $f\left( {X_{E} } \right) > f\left( {X_{R} } \right)$, $f\left( {X_{R} } \right)$ replaces $X_{D + 1}$ to construct a new simplex.
Step 6 Compression operation of the reflected point. If $f\left( {X_{R} } \right) \le f\left( {X_{D + 1} } \right)$, according to Eq. (26), the compression point $X_{CR}$ is obtained. If $f\left( {X_{CR} } \right) \le f\left( {X_{D + 1} } \right)$, then $X_{CM}$ is used instead of $X_{D + 1}$ to construct a new simplex. Otherwise, utilizing Eq. (27), calculate the new point $X_{CD}$. If $f\left( {X_{CM} } \right) \le f\left( {X_{D + 1} } \right)$, then $X_{CD}$ is used instead of $X_{D + 1}$ to construct a new simplex.
  26 $$X_{CR} = X_{C} + \gamma \times \left( {X_{R} - X_{C} } \right)$$
  27 $$X_{CD} = X_{C} + \gamma \times \left( {X_{D + 1} - X_{C} } \right)$$
  where $\gamma$ is the compression coefficient.
Step 7 Overall contraction operation. If $f\left( {X_{CM} } \right) > f\left( {X_{D + 1} } \right)$, then the simplex is compressed, and the operation in **Eq. **(28) is performed.
  28 $$X_{i} = X_{1} + \omega \times \left( {X_{i} - X_{1} } \right),i = 2,3,...,D,D + 1$$

In this paper, the current RIME agent performs the NMs operation only when $r_{3} < NMsPRO$. Additionally, the simplex operation is executed on $R_{{{\text{best}}}}$ at the end of each iteration. The random number $r{}_{5}$ in the range $\left[ {0,1} \right]$ and $NMsPRO$ represents the probability of performing the NMs operation. In this paper, we set the specific value of $NMsPRO$ to 0.1.

**Fig. 5:**
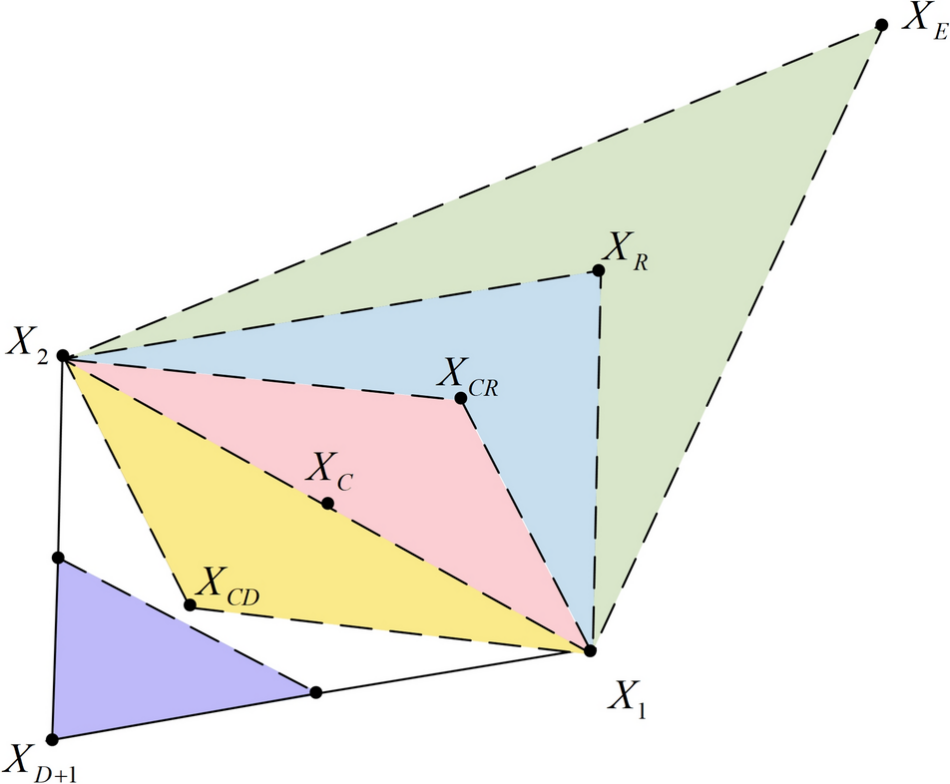
The visualization of NMs.

The NMs can be represented by Fig. 5.

### Dynamic multi-dimensional random mechanism

This section will elaborate on the proposed dynamic multi-dimensional random mechanism (DMRM).

In the original RIME, the update of the rime particle heavily relies on $R_{best,j}$. As shown in Eqs. (17) and (18), the variation of $\cos \theta$ is periodic, fluctuating between − 1 and 1, rather than changing randomly. During the search process, this periodic behavior causes the rime particle to become overly concentrated in certain local regions, affecting RIME’s performance in complex problems. Although $r_{1}$ introduces small disturbances, which somewhat increases the randomness of the search, it still makes it difficult for the RIME particles to effectively cover the area around $R_{best}$, hindering the particles from escaping local optima.

To overcome this issue, we introduce DMRM into the original RIME framework. By using uncertain disturbances and non-periodic randomness, DMRM breaks the original periodic constraint, making the particle search trajectory more random and diversified. As a result, the particles can escape local optima and explore the solution space more evenly. This is particularly beneficial in regions near $R_{best}$, where better coverage and search capability can be achieved, significantly improving the algorithm’s global optimization performance and convergence speed.

**Fig. 6:**
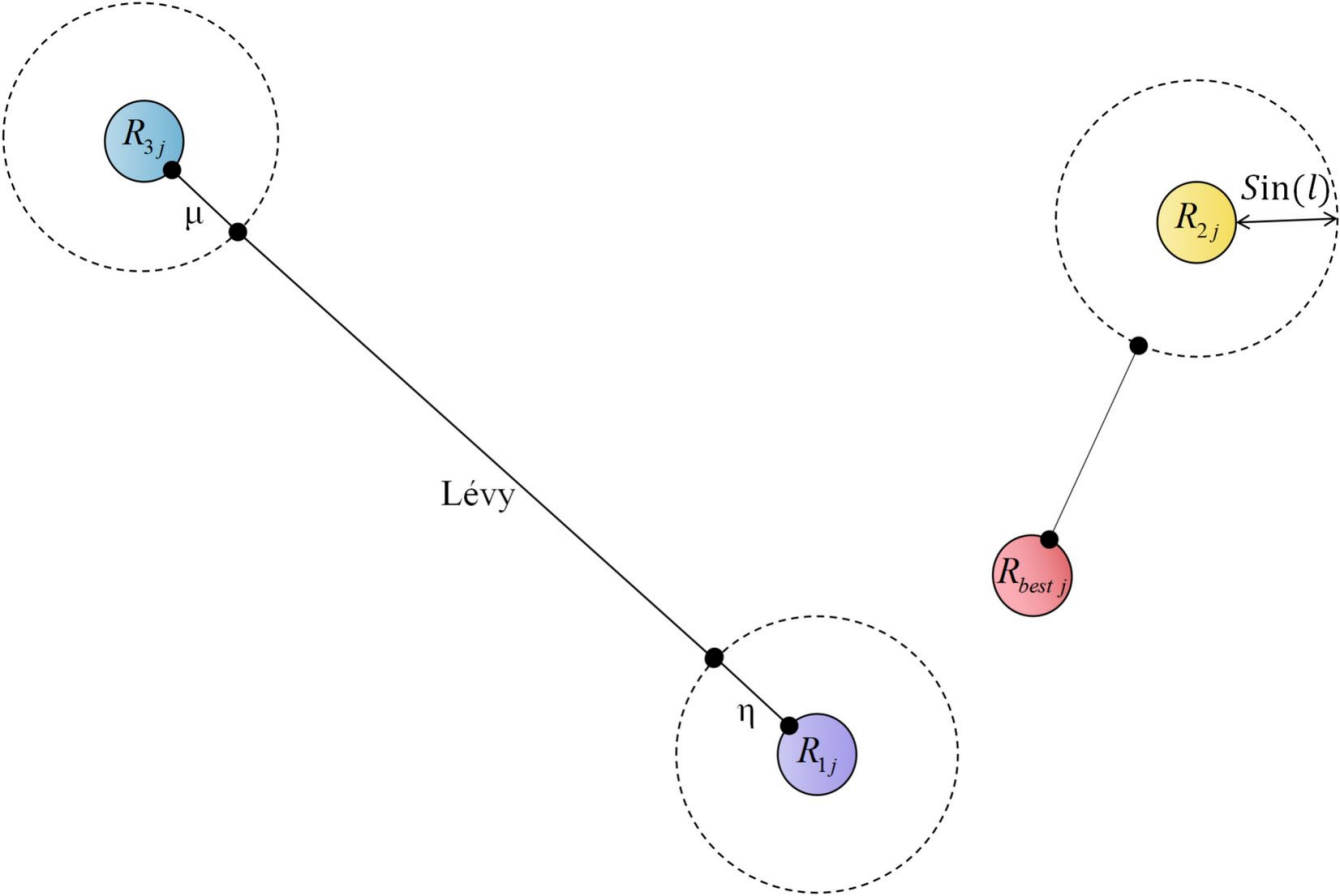
Structure of DMRM.

**Fig. 7:**
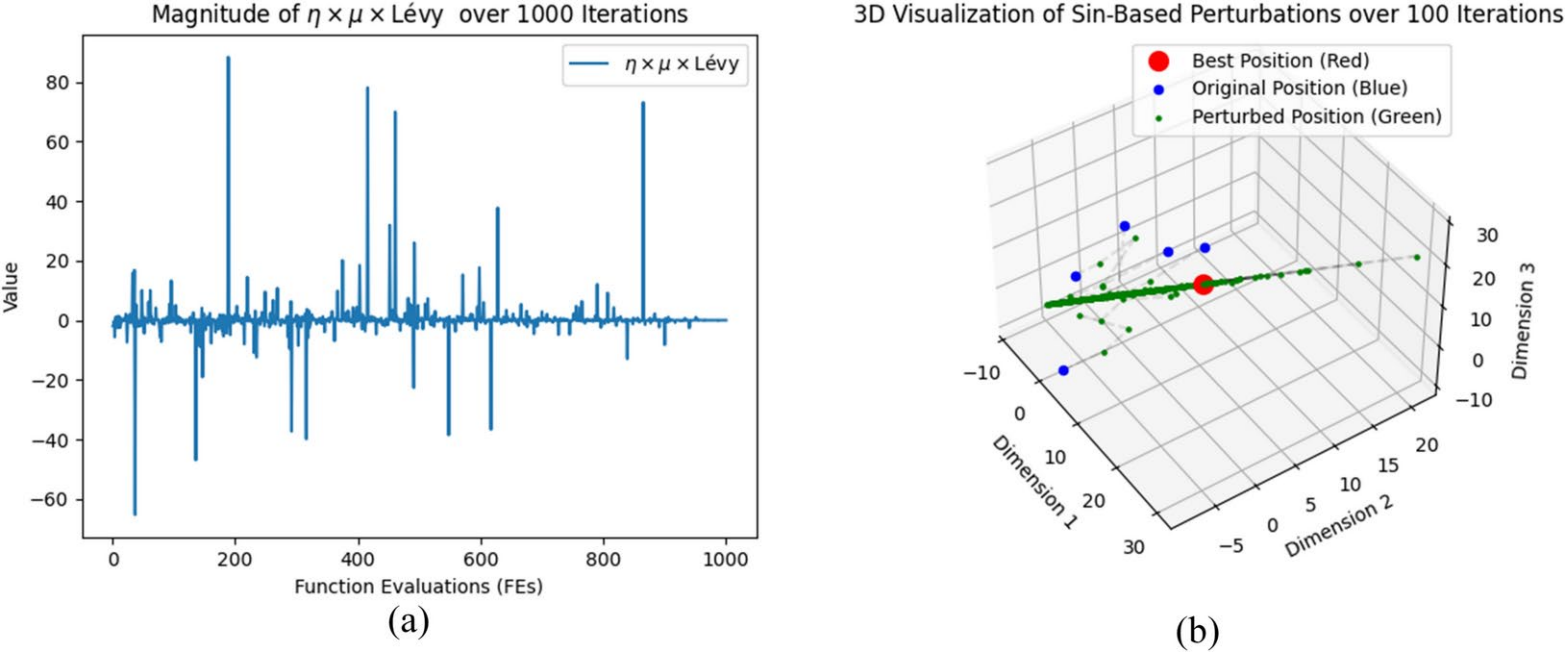
Variation of key variables across iterations.

Therefore, we reinforce the particle update strategy using Eq. (29), introducing a more uncertain disturbance mechanism to increase the search space’s diversity, thereby enhancing RIME’s global search ability. At the same time, Eq. (35) increases the search capability of RIME particles around $R_{best}$. While the values $\sin \left( l \right)$ also range from -1 to 1, due to the randomness introduced by Eq. (36), the values $\sin \left( l \right)$ do not exhibit a fixed periodicity. Figure 6 illustrates the concept of DMRM. Figure 7 illustrates the trend of important variables over time during different iterations.

29 $$R_{ij}^{new} = \mu \times L\mathop e\limits^{\prime } vy \times \left( {R_{ij} + \eta } \right),j \in J$$

30 $$\eta = \left( {UB - LB} \right) \times 0.1 \times \left( {2 \times r_{4} - 1} \right)$$

31 $$\mu = 1 - \frac{FEs}{{MaxFEs}}$$

32 $$J = \left\{ {j_{1} ,j_{2} , \ldots ,j_{k} } \right\},j_{i} \in \left\{ {1,2, \ldots ,dim} \right\},\forall i \in \left\{ {1,2, \ldots ,k} \right\}$$

33 $$k = \left\lfloor {\dim \times \varepsilon } \right\rfloor$$

34 $$\varepsilon = \lambda \times e^{ - \delta \cdot MaxFEs}$$

where $J$ represents a set of $k$ dimensions randomly selected from $\left\{ {1,2, \ldots ,dim} \right\}$. $k$ is the number of selected dimensions, generated by Eq. (33). $\varepsilon$ is the dimension scaling factor, which controls the range of dimension selection, as specified by Eq. (34). $\lambda$ is set to 0.1, representing the initial dimension selection range. $\delta$ is set to 0.05, representing the decay rate. *Lévy* represents a random number that follows a Lévy distribution. $\mu$ is a linearly decreasing factor that decreases from 1 to 0 as the $FEs$ increase, as shown in Eq. (31). $\eta$ is a dimension perturbation factor, calculated as Eq. (30). The parameter $r_{4}$ is a random number in the range $\left[ {0,1} \right]$.

35 $$R_{ij}^{new} = R_{ij} \times \left| {\sin \left( l \right)} \right| - \frac{9}{16} \times \sin \left( l \right) \times \left| {R_{ij}^{best} + R_{ij} } \right|,j = 1,2, \ldots ,\dim$$

36 $$l = 2 \times r_{6} \times \pi$$

where $l$ is a random angle that controls the value of the sine function, derived from Eq. (36). $r_{6}$ is a random number in the range $\left[ {0,1} \right]$.

### The proposed DNMRIME algorithm based on RIME

Based on this, we propose an enhanced RIME that combines the DMRM with the NMs, referred to as DNMRIME. The DNMRIME inherits the fundamental framework of the RIME while boasting improved global and local exploration capabilities. In the DNMRIME algorithm, DMRM is utilized to increase fitness values and escape local optima. At the same time, the NMs are employed for local search to further enhance the accuracy of solutions and the local search capability of the algorithm. By combining these two mechanisms, DNMRIME achieves a good balance between global and local exploration while effectively overcoming the challenge of local optima.

The positive greedy absorption mechanism, a commonly used strategy in optimization algorithms^82,83^, was adopted in the original RIME^78^. Therefore, we maintain its application in updating the population throughout the entire algorithm, safeguarding the superiority of our solutions.

Specifically, the design logic of DNMRIME is to dynamically adjust the neighborhood range of the search space during the iterative optimization process, integrating the greedy absorption DMRM, and NMs simplex to solve optimization problems efficiently.

**Fig. 8:**
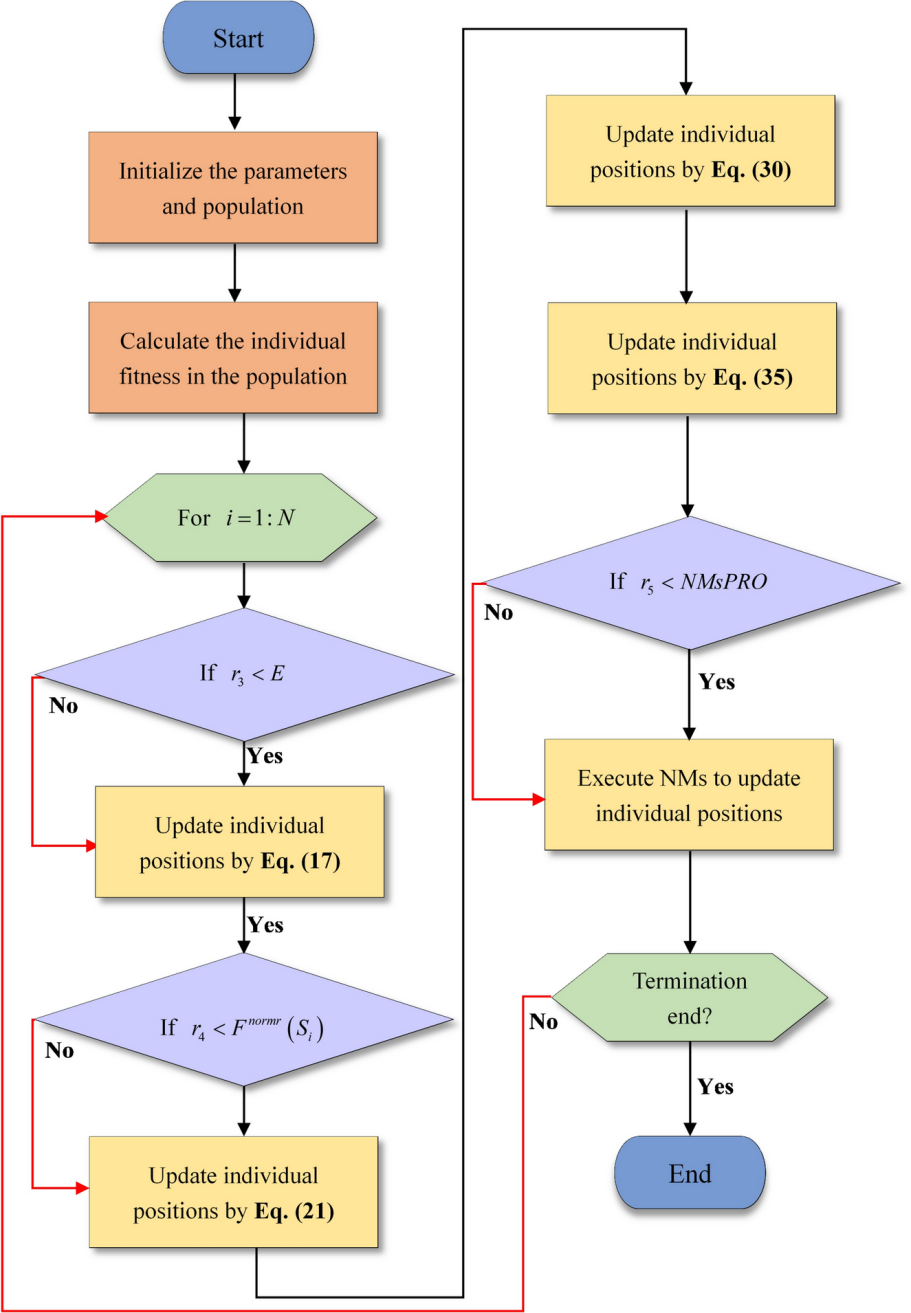
The flowchart of DNMRIME.

To vividly illustrate DNMRIME, the flowchart is depicted in Fig. 8, while the pseudocode is summarized in Algorithm 1.

**Algorithm 1:**
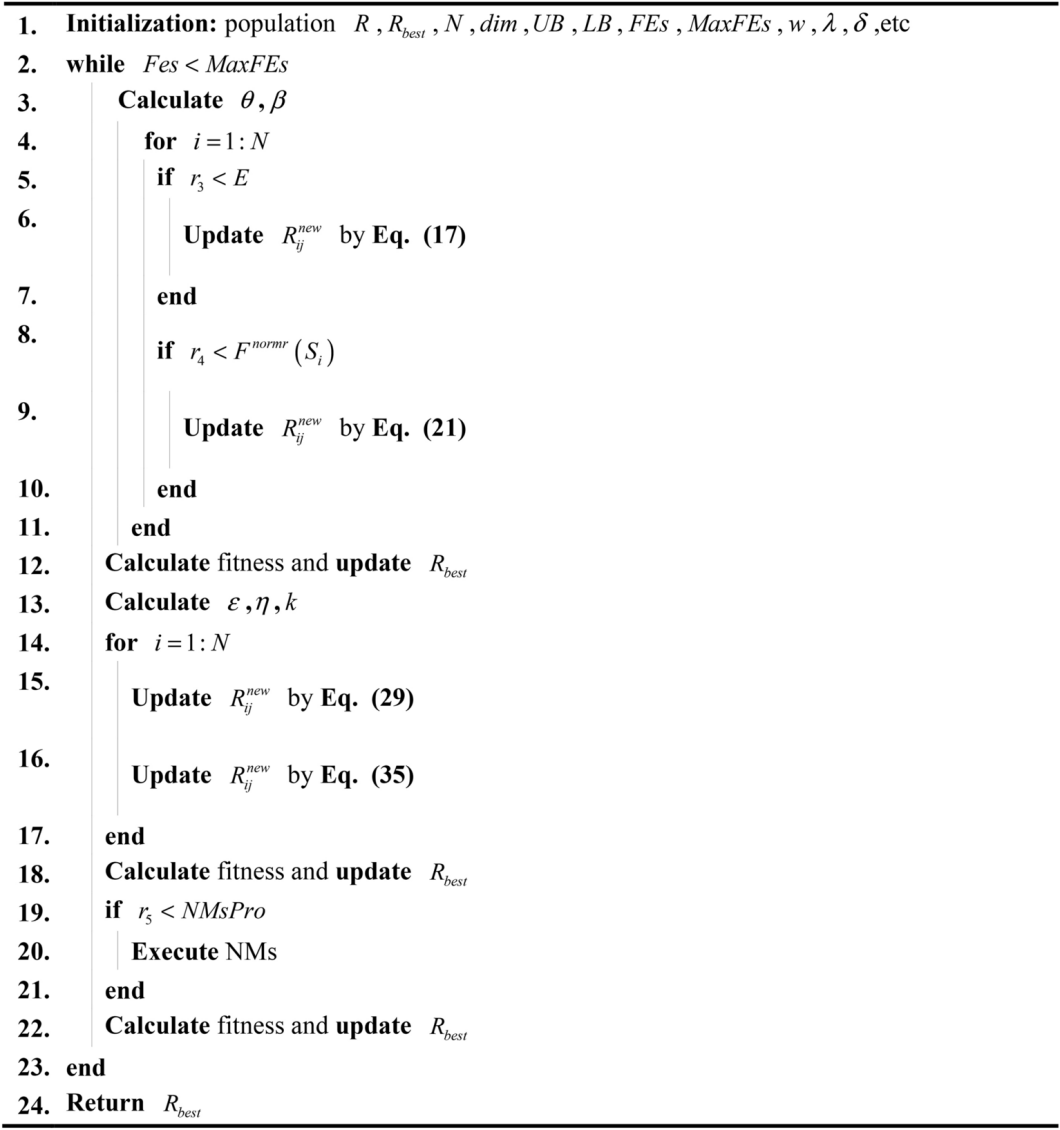
Pseudo-code of DNMRIME.

## Experimental results

In this section, we present a comprehensive overview of the experimental results.

Firstly, a qualitative analysis of DNMRIME is conducted. Secondly, an ablation study is performed to examine the specific contributions of various mechanisms to the performance of DNMRIME. Thirdly, we assess and compare the performance of DNMRIME with other well-known MAs, benchmarking against CEC 2017. Fourthly, we contrast DNMRIME with algorithms renowned for superior performance in solar models, evaluating and comparing them across SDM, DDM, TDM, and PV. Lastly, we validate the effectiveness of DNMRIME using actual data provided by suppliers in different environments, including KC200GT, ST40, and SM55.

The experimental equipment configuration adopted in this study is shown in Table 2. All experiments in this paper are based on the following configuration.

**Table 2:** Experimental equipment configuration.

Configuration item	Details
Processor	Intel® Core™ i5-12500
Clock speed	3.00 GHz
RAM capacity	16.0 GB
Operating system	Windows 11
MATLAB version	MATLAB 2018

To conduct a detailed analysis of the experimental results, we will utilize the following metrics to evaluate the performance of DNMRIME:

1. 2D Search History: This metric shows how the population converges towards the global optimum across different iterations (used in “Qualitative analysis of DNMRIME on CEC 2017”).
2. The first dimension of trajectory: It reflects the variation in the first dimension of the population during the iterations. Initially, with a good algorithm, there are significant fluctuations, which gradually stabilize and converge to the global optimum (used in “Qualitative analysis of DNMRIME on CEC 2017”).
3. Diversity: This metric evaluates the diversity of the population members during the search process, as is shown in Eqs. (37) and (38) (used in “Qualitative analysis of DNMRIME on CEC 2017”).
  37 $$Div_{j} = \frac{1}{N}\sum\limits_{i = 1}^{n} {\left| {meadian\left( {x^{j} } \right) - x_{i}^{j} } \right|}$$
  38 $$Div = \frac{1}{dim}\sum\limits_{j = 1}^{dim} {Div_{j} }$$
  where $Div$ represents the diversity of all population members in the algorithm. $Div_{\max }$ represents the maximum diversity value in the population members. $Div_{j}$ represents the diversity of the $j$-th dimension in the population.
4. Exploration and exploitation: These metrics assess the algorithm’s exploration–exploitation balance, as defined in Eqs. (39) and (40) (used in “Qualitative analysis of DNMRIME on CEC 2017”).
  39 $$Exploration\left( \% \right) = \frac{Div}{{Div_{\max } }} \times 100\%$$
  40 $$Exploitation\left( \% \right) = \frac{{\left| {Div - Div_{\max } } \right|}}{{Div_{\max } }} \times 100\%$$
  where $Exploration$ denotes the exploration percentage of the algorithm, while $Exploitation$ denotes its exploitation percentage.
5. Average (Mean) and Standard deviation (Stdv, Std): The mean reflects the algorithm’s average performance, while the Std measures the performance stability. Outstanding experimental results will be highlighted in bold (used in “DNMRIME for ablation study on CEC 2017” and “DNMRIME compared with well-known algorithms”).
6. Wilcoxon signed-rank test (WSRT): We employ the non-parametric Wilcoxon signed-rank test^84^ to quantify the significance of algorithm performance improvements. In this test, we set the significance level to 0.05 and use "+/=/−" symbols to indicate whether DNMRIME performs better than, equal to, or worse than other MAs (used in “DNMRIME for ablation study on CEC 2017” and “DNMRIME compared with well-known algorithms”).
7. Root mean square error (RMSE): RMSE measures the discrepancy between an algorithm’s predicted values and actual results, with a lower value indicating greater prediction accuracy.
8. Convergence curves: Convergence curves visually depict the optimization progress of an algorithm, enabling a clear comprehension of its convergence speed and stability throughout the process (used in “Experimental results of DNMRIME on SDM”–“Experimental results of DNMRIME on PV”).
9. P–V and I–V: The photovoltaic parameters extracted by the algorithm can be assessed using the I–V and P–V characteristics (used in “Experimental results of DNMRIME on SDM”–“Experimental results of DNMRIME on PV”, “Results of different irradiation and constant temperature”–“Results of different temperature and constant irradiation”).
10. I-IAE, I-RE, P-IAE, P-RE error characteristics: We will utilize metrics I-IAE, I-RE, P-IAE, and P-RE to assess the performance of DNMRIME in extracting photovoltaic parameters. These metrics respectively represent the absolute and relative errors for current (I-IAE, I-RE) and power (P-IAE, P-RE). The equations for calculating IAE and RE are shown in Eqs. (41) and (42), respectively (used in Sections “Experimental results of DNMRIME on SDM”–“Experimental results of DNMRIME on PV”).
  41 $$IAE = \sum\limits_{i = 1}^{n} {\left| {X_{{{\text{actual}}}} - X_{predict} } \right|}$$
  where $X_{{{\text{actual}}}}$ represents the actual data, $X_{predict}$ represents the predicted data from the photovoltaic simulation, and $n$ is the number of data points.
  42 $$RE = \frac{{\sum\nolimits_{i = 1}^{n} {X_{{{\text{actual}}}} - X_{predict} } }}{{\sum\nolimits_{i = 1}^{n} {X_{predict} } }} \times 100\%$$
  where $X_{{{\text{actual}}}}$ represents the actual data, $X_{predict}$ represents the predicted data from the photovoltaic simulation, and $n$ is the number of data points.
11. CPU Cost Time: This metric measures the computational time required by the algorithm, reflecting its efficiency in terms of resource usage (used in “CPU time cost assessment”).

### Results of DNMRIME on CEC 2017

CEC 2017 is an internationally recognized benchmark set. All the benchmark functions used in “Results of DNMRIME on CEC 2017” are sourced from CEC 2017, consisting of 30 functions across 4 different types. The value range for these functions is $\left[ { - 100, \, 100} \right]$, as shown in Table 3, assessing the optimization performance of DNMRIME. These 30 functions are used as objective functions, with DNMRIME aiming to minimize their values for optimization.

**Table 3:** The summary of CEC 2017.

Function	Type	Purpose	Search range
F1–F3	Unimodal functions	Evaluate local exploitation ability	$\left[ { - 100,100} \right]^{D}$, where $D$ is the dimension
F4–F10	Multimodal functions	Assess global exploration ability	
F11–F20	Hybrid functions	Evaluate robustness of algorithm	
F21–F30	Composition functions	Assess problem-solving ability	

To ensure the comparability and fairness of the experimental results, uniform settings were applied to all algorithm parameters in “Qualitative analysis of DNMRIME on CEC 2017”–“DNMRIME compared with well-known algorithms”, as shown in Table 4.

**Table 4:** Unified parameter settings.

Section			
4.1.1		4.1.2, 4.1.3	
Parameter	Value	Parameter	Value
$dim$	$30$	$dim$	$30$
$N$	$10$	$N$	$30$
$MaxFEs$	500	$MaxFEs$	300,000
–	–	Runs	$30$

#### Qualitative analysis of DNMRIME on CEC 2017

To better demonstrate the performance of DNMRIME, the qualitative analysis will be conducted from two aspects: the historical search patterns of the algorithm and the balance between exploitation and exploration. Furthermore, on CEC 2017, we conducted an experiment utilizing F1, F4, F7, F25, and F28 to assess the effectiveness of DNMRIME.

**Fig. 9:**
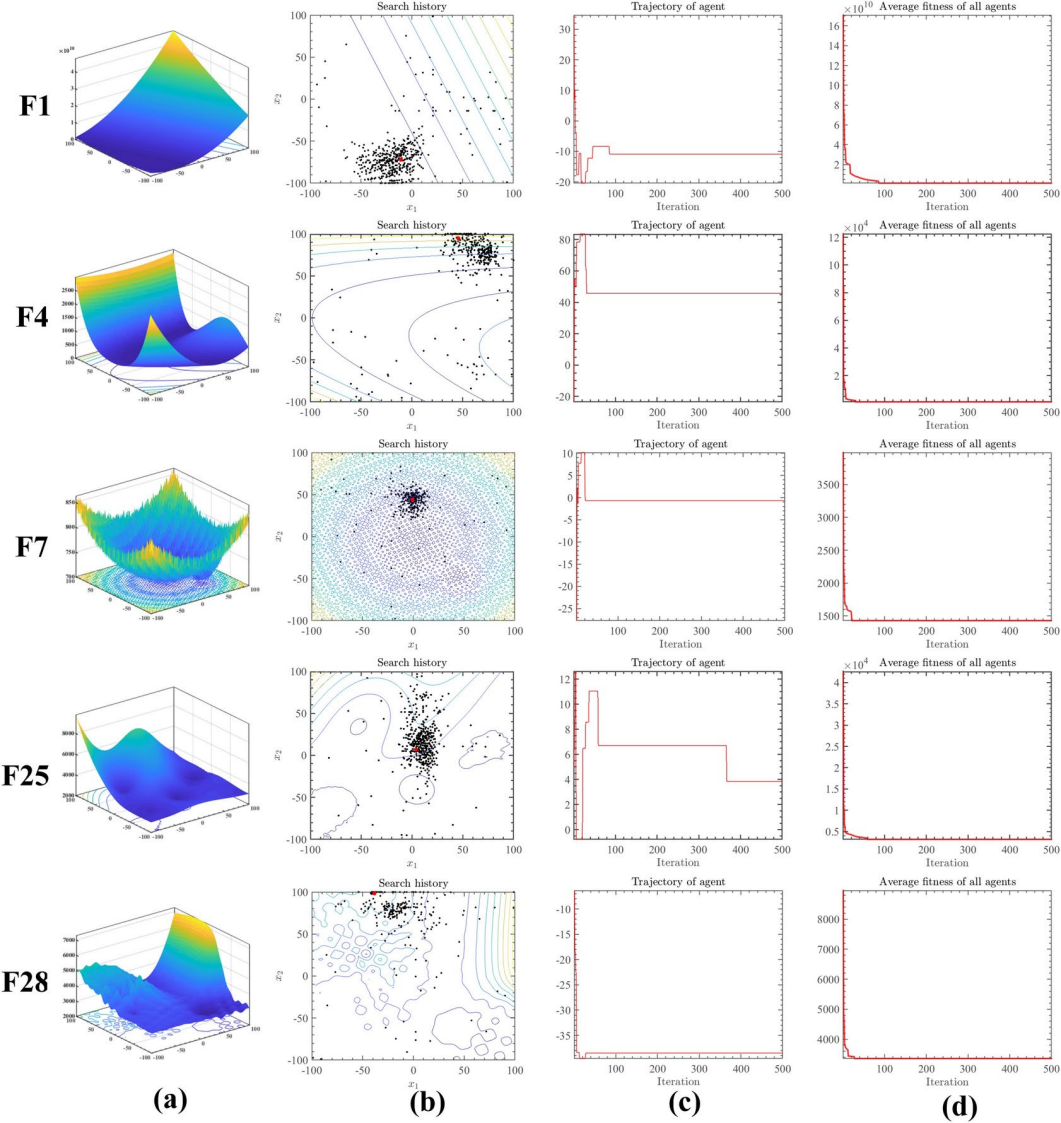
(**a**) Function diagram, (**b**) 2D search history of DNMRIME, (**c**) the first dimension of trajectory in DNMRIME, (**d**) average fitness of the DNMRIME.

Figure 9 presents one of the qualitative analysis results of DNMRIME. Specifically, Fig. 9a in the second column showcases the three-dimensional image of the corresponding function, with the red dot indicating the global optimal solution obtained by DNMRIME. Figure 9b in the second column depicts the search history of DNMRIME in a two-dimensional distribution. Figure 9c in the third column illustrates the changing pattern of the search positions of the RIME agents in the first dimension. Finally, Fig. 9d in the third column displays the average fitness of DNMRIME.

The experimental results show that DNMRIME exhibits a wide population distribution, effectively covering the solution space. This indicates its strong global exploration capability. Moreover, DNMRIME can closely reach the global optimum with fewer populations. During the initial stage, DNMRIME undergoes sharp changes in the first dimension, indicating its focus on exploration. During the later iterations, the stability of the first dimension reflects the convergence of DNMRIME towards the optimal solution.

**Fig. 10:**
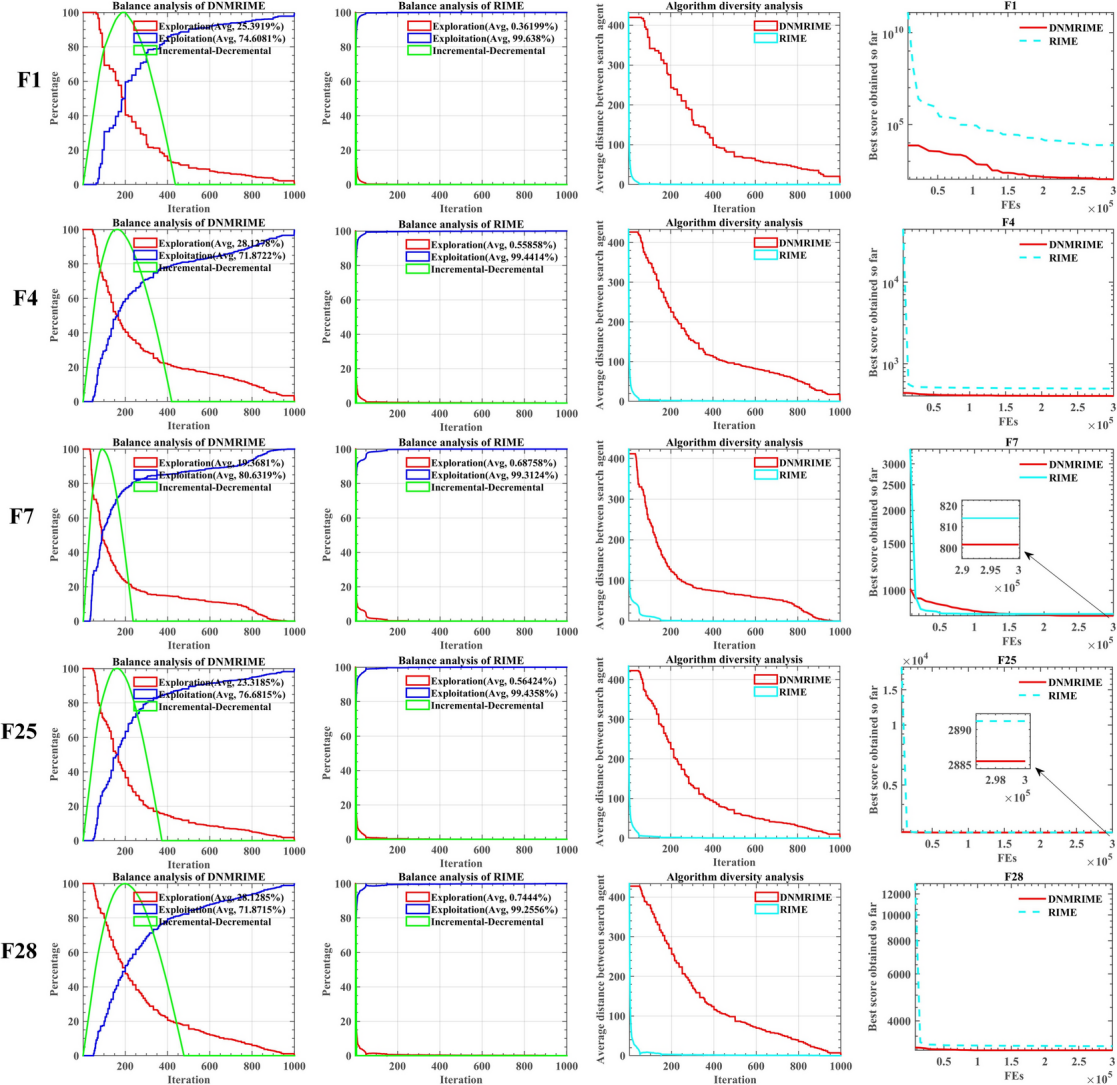
(**a**) Balance analysis of the DNMRIME, (**b**) balance analysis of the RIME, (**c**) diversity analysis, (**d**) convergence curve.

Similarly, we compared DNMRIME and RIME using five distinct functions to analyze balance and diversity. Figure 10a shows balance analysis results for DNMRIME, while Fig. 10b shows those for RIME. Figure 10c displays the diversity experiment results of DNMRIME and RIME, while Fig. 10d shows convergence curves of fitness.

As a result, DNMRIME exhibits a significant enhancement in exploration capabilities compared to the original RIME. Furthermore, the diversity exhibited by DNMRIME is richer than RIME’s, leading to superior performance in the convergence curves of DNMRIME’s fitness. F1 and F7 demonstrate the capacity of DNMRIME to overcome local optima, while F4, F25, and F28 showcase its rapid convergence speed. Compared to RIME, DNMRIME boasts superior optimization capabilities and a faster convergence speed. However, it must be acknowledged that, in the later stages of the diversity experiment, the convergence effect of DNMRIME may not be as satisfactory as RIME. This could be attributed to the fact that, while maintaining diversity, DNMRIME continues to explore new search directions.

#### DNMRIME for ablation study on CEC 2017

An ablation study is a crucial research tool that involves removing or substituting specific components in a system or model to observe their effects on performance. Similar to controlling variables, it deepens our understanding of each component’s role and supports optimization.

In this section, we will separately test the effects of the two mechanisms of DNMRIME on the CEC 2017 function set to verify the superiority of DNMRIME over DRIME and NMRIME.

**Fig. 11:**
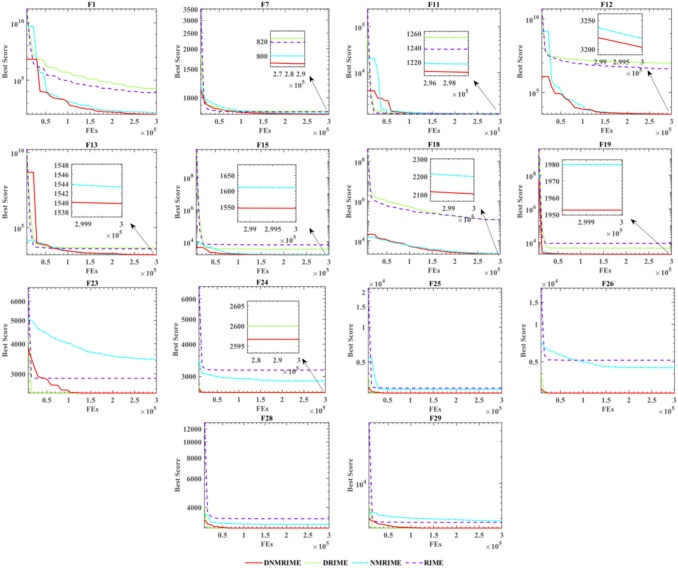
Convergence curve of DNMRIME for ablation study on CEC 2017.

The ablation experimental results on CEC 2017 are shown in Fig. 11. Appendix Table 1 shows the results of the ablation study. Table 5 shows the WSRT comparison results o. It demonstrated the best performance among the 14 functions and tied for first place in F25, F26, F28, F29 with DRIME. Through the ablation experiments conducted on CEC 2017, we initially verified the potential effectiveness of integrating the two mechanisms into DNMRIME. The experimental results suggest that RIME incorporating the two mechanisms may better solve complex optimization problems than algorithms adopting only one mechanism.

**Table 5:** WSRT comparison of DNMRIME for ablation study.

Algorithm	+/=/−	Mean	Rank
DNMRIME	–	2.03	1
DRIME	11/9/10	2.48	2
NMRIME	18/12/0	2.79	4
RIME	18/2/10	2.70	3

#### DNMRIME compared with well-known algorithms

In this section, we will compare DNMRIME with several original and advanced algorithms that have shown outstanding performance on the CEC 2017. These algorithms include RIME^78^, DE^24^, PSO^23^, WOA^26^, HHO^28^, WSO^85^, AHA^86^, LSHADE^87^, LSHADE_cnEpSi^88^, CLPSO^89^, ALCPSO^90^, SCADE^91^, GAEFA_HK^92^, and iAEFA^93^. The specific algorithm settings used in this study are detailed in Table 6.

**Table 6:** Parameter setting of comparison algorithm with DNMRIME.

Algorithm	Parameters setting
DNMRIME	$W = 5;NMs\Pr o = 0.1;\delta { = 0}{\text{.05; }}\lambda { = 0}{\text{.1}}$
RIME	$W = 5$
DE	$pCR = 0.2;beta \in \left[ {0.2,0.8} \right]$
PSO	$V_{\max } = 6;noP = N;w_{\max } = 0.9;w_{\min } = 0.2;c_{1} = 2;c_{2} = 2$
WOA	$a_{1} = \left[ {0,{ 2}} \right];a_{2} = \left[ { - 2, \, - 1} \right];b = 1$
HHO	$beta = 1.5;c = 2 * \left( {1 - \frac{FEs}{{MaxFEs}}} \right)$
WSO	$\rho_{r} = 0.5$
AHA	$Migration \, coefficient = 2N$
LSHADE	$arc\_rate = 0.4;memory\_sf = memory\_cr = 0.5;p_{best} = 0.11;memory\_size = 5$
LSHADE_cnEpSi	$freq\_init = ps = 0.5;pb = 0.4$
CLPSO	$c \, = \, 1.49445$
ALCPSO	$w = 0.4;c_{1} = c_{2} = 2;lifespan = 60;T = 2$
SCADE	$min{ = 0}{\text{.2; }}a{ = 2; }CR{ = }max{ = 0}{\text{.8}}$
GAEFA_HK	$k_{0} = 500;\alpha = 6;T = 300$
iAEFA	$k_{0} = 500;\alpha \in \left[ {3,40} \right]$

**Fig. 12:**
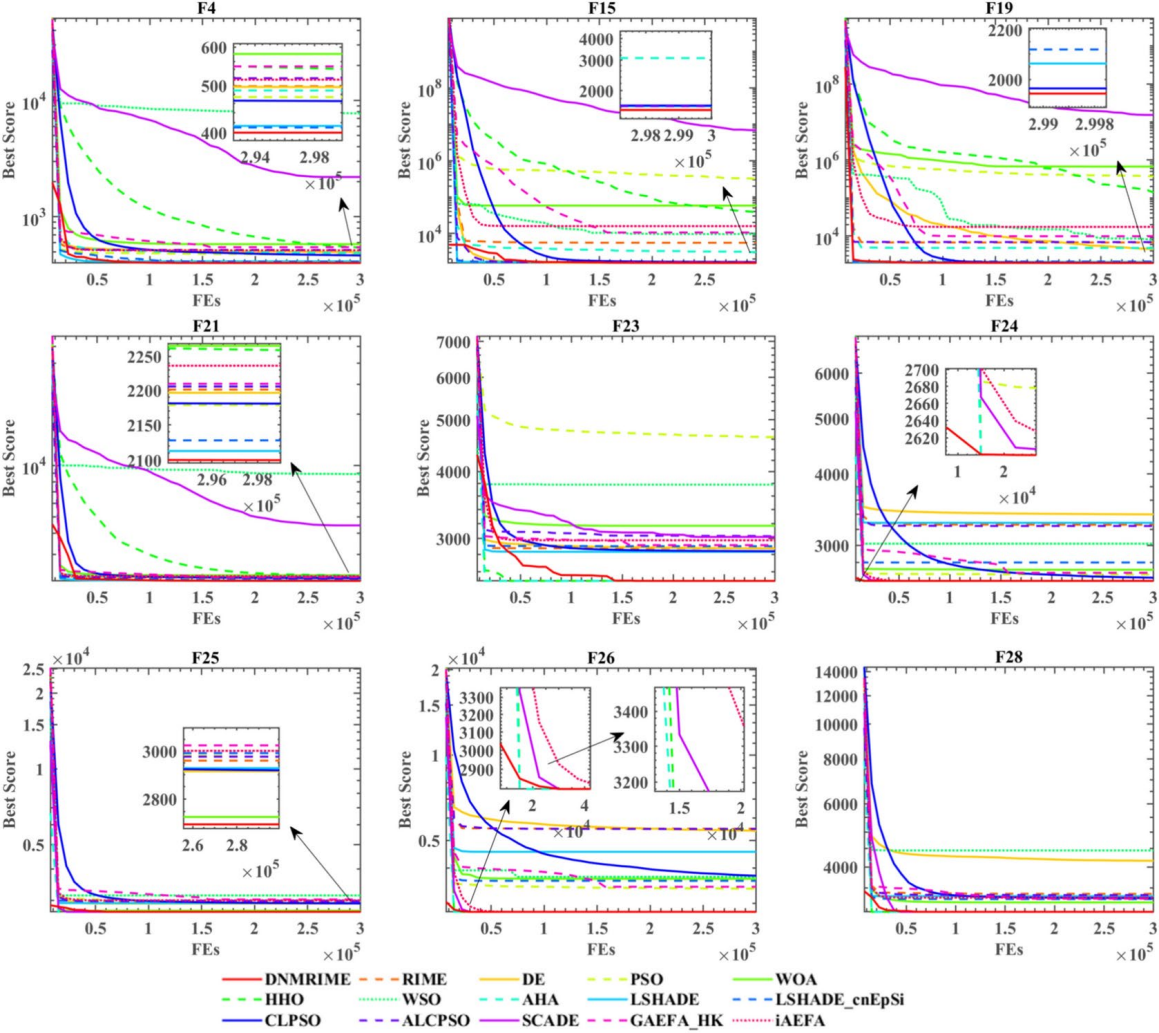
Convergence comparison of DNMRIME with well-known algorithms on CEC 2017.

As is showed in Fig. 12, which presents the convergence curves of each algorithm, it can be observed that on CEC 2017, DNMRIME converges faster on F9 and F28. Most algorithms can find relatively good global optimum solutions quickly for the multimodal, hybrid, and composition functions such as F4, F15, F19, F21, F25, and F28, but DNMRIME demonstrates superior convergence accuracy. Appendix Tables 2–5 present the specific comparison results of DNMRIME with well-known MAs. As shown in Appendix Table 2, DNMRIME ranks third in terms of fitness mean on the single-modal function F1, performing worse than LSHADE and LSHADE_cnEpSi.

Table 7 shows that on CEC 2017, DNMRIME ranks first, outperforming the second-ranked LSHADE in 14 functions, being equal to LSHADE in 3 functions, and performing worse in 13 functions.

**Table 7:** WSRT comparison of DNMRIME with well-known algorithms.

Algorithm	+/=/−	Mean	Rank
DNMRIME	–	4.486	1
RIME	17/5/8	7.162	7
DE	17/4/9	6.864	6
PSO	30/0/0	10.902	12
WOA	27/3/0	11.401	13
HHO	22/4/4	8.867	11
WSO	30/0/0	12.097	15
AHA	17/7/6	5.929	5
LSHADE	14/3/13	4.494	2
LSHADE_cnEpSi	16/2/12	5.268	3
CLPSO	17/2/11	5.381	4
ALCPSO	22/3/5	8.168	8
SCADE	24/5/1	11.627	14
GAEFA_HK	22/1/7	8.510	9
iAEFA	23/0/7	8.843	10

### Experimental results of DNMRIME on solar models

In this section, we will conduct photovoltaic simulation experiments on DNMRIME.

Fairly, Table 8 shows the boundaries for unknown parameters. To validate the accuracy and efficiency of DNMRIME, we will compare it not only with the original RIME^78^ but also with other outstanding algorithms on solar models, specifically CCNMHHO^94^, GOFPANM^95^, WOA^26^, BSA^96^, TLBO^30^.

**Table 8:** Upper and lower bounds of unknown parameters.

Parameter	SDM/DDM/TDM		PV/KC200GT/ST40/SM55	
	Lowest bound	Highest bound	Lowest bound	Highest bound
$I_{ph} (A)$	0	1	0	2
$I_{sd} (\mu A)$, $I_{sd1} (\mu A)$, $I_{sd2} (\mu A)$, $I_{sd3} (\mu A)$	0	1	0	50
$n$, $n_{1}$, $n_{2}$, $n_{3}$	1	2	1	50
$R_{s}$ (Ω)	0	0.5	0	2
$R_{sh}$ (Ω)	0	100	0	2000

#### Experimental results of DNMRIME on SDM

**Fig. 13:**
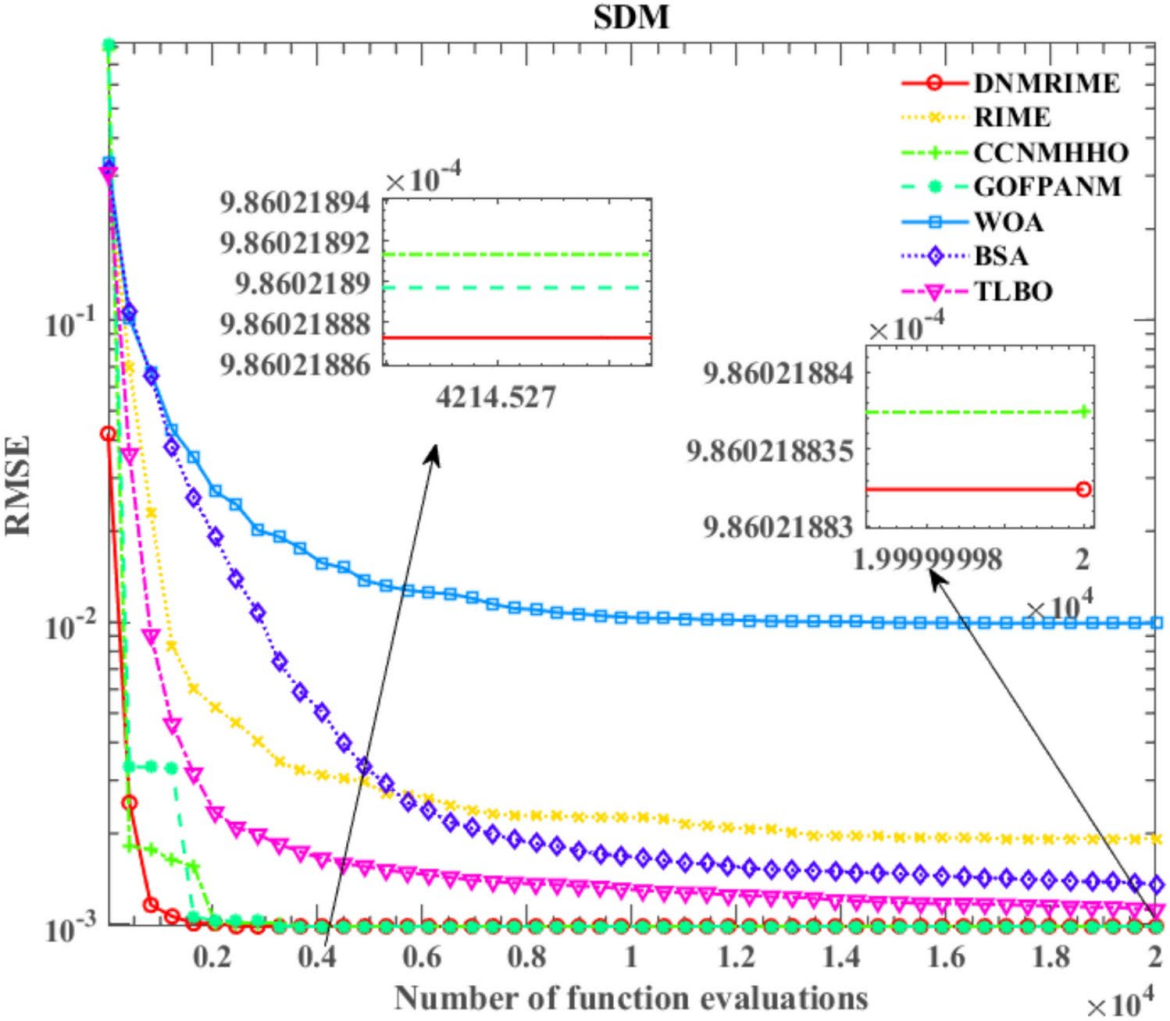
Convergence comparison of algorithms in the SDM.

**Fig. 14:**
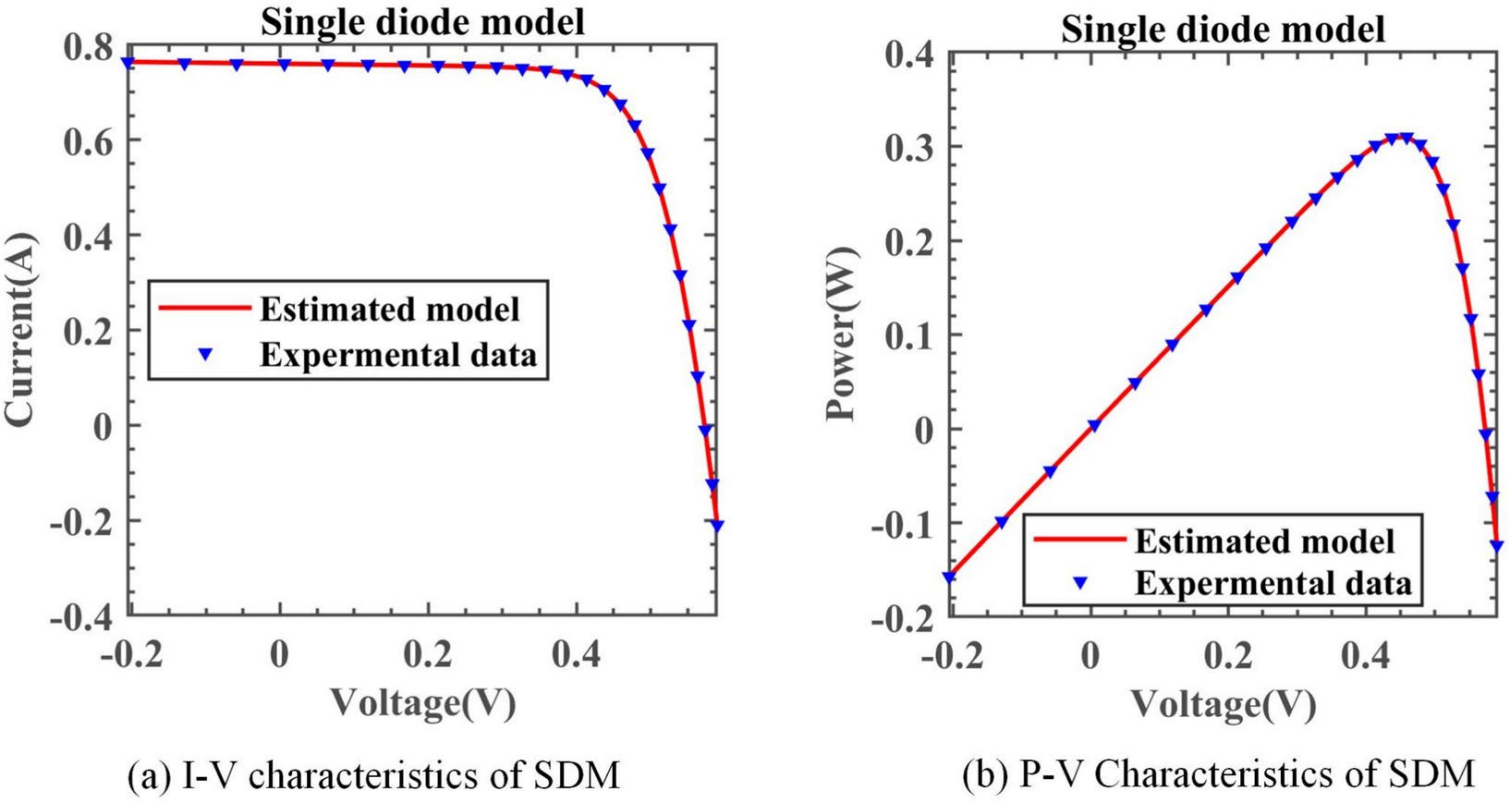
I–V and P–V characteristic curve of DNMRIME on SDM.

**Fig. 15:**
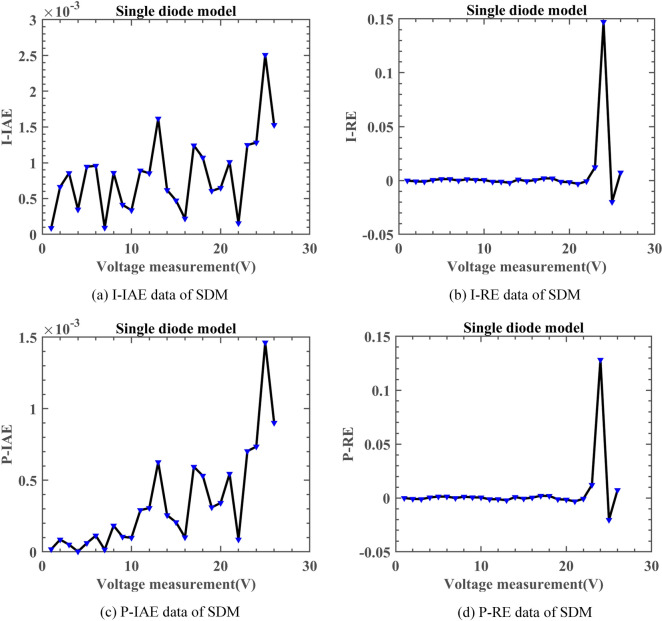
Error metrics of DNMRIME on SDM.

Table 9 summarizes the results of parameter identification on SDM. In Fig. 13, the solid red line represents DNMRIME, while the curves of different colors represent various comparative algorithms. The RMSE convergence curves in this section and the following three subsections are based on the mean RMSE values. Additionally, we calculated the RMSE values of algorithms on SDM and included them in Table 10. Figure 14 illustrates the performance of the SDM at different voltages: Fig. 14a,b depict the I–V and P–V characteristic curves, respectively. Figure 15 presents the various error metrics of DNMRIME on SDM.

**Table 9:** Parameters estimation results of DNMRIME with other algorithms on SDM.

Item	DNMRIME	RIME	CCNMHHO	GOFPANM	WOA	BSA	TLBO
${\text{I}}_{{{\text{ph}}}} {\text{(A)}}$	0.76077547	0.76100100	0.76077556	0.76077553	0.76131261	0.76061651	0.76073870
${\text{I}}_{{{\text{sd}}}}$ (μA)	0.32302698	0.33391613	0.32302564	0.32302080	0.75370353	0.33799831	0.32722285
${\text{R}}_{{\text{s}}}$ (Ω)	3.63770053E − 02	3.62476584E − 02	3.63769972E − 02	3.63770928E − 02	3.26375670E − 02	3.61848079E − 02	3.63234160E − 02
${\text{R}}_{{{\text{sh}}}}$ (Ω)	53.71901421	52.02512650	53.71832947	53.71851963	75.34789413	56.00141430	54.53637226
$n$	1.48118553	1.48456871	1.48118511	1.48118359	1.57179273	1.48573978	1.48247484
$RMSE$	**9.8602E − 04**	1.0018E − 03	**9.8602E − 04**	**9.8602E − 04**	1.9863E − 03	9.9419E − 04	9.8686E − 04
Compare		+	=	=	+	+	+

Significant values are in bold.

**Table 10:** RMSE values of DNMRIME and other algorithms on SDM.

Item	$Max$	$Min$	$Mean$	$Std$
DNMRIME	9.86021889E − 04	9.86021880E − 04	**9.8602188324E − 04**	**2.00339E − 12**
RIME	7.31804270E − 03	1.00179682E − 03	1.9226893260E − 03	0.001164931
CCNMHHO	9.86021893E − 04	9.86021880E − 04	9.8602188374E − 04	3.17257E − 12
GOFPANM	9.86021903E − 04	9.86021878E − 04	9.8602188970E − 04	5.75592E − 12
WOA	4.41134266E − 02	1.98632656E − 03	9.9302641932E − 03	0.009776725
BSA	1.88397447E − 03	9.94189070E − 04	1.3604541357E − 03	0.000224506
TLBO	1.39656941E − 03	9.86856871E − 04	1.1167603686E − 03	0.000133876

Significant values are in bold.

From the perspective of convergence speed, DNMRIME demonstrates significant superiority. By observing the convergence curves, DNMRIME exhibits stronger optimization capability, achieving lower RMSE values in the early iterations than RIME. The convergence curve of DNMRIME rapidly descends, indicating its ability to reach lower RMSE values with fewer function evaluations. In contrast, the convergence speed of RIME, WOA, BSA and TLBO is relatively slower, and their RMSE values are far higher than those of DNMRIME. GOFPANM and CCNMHHO, known for their strong performance in photovoltaic parameter estimation, require more iterations to match the RMSE of DNMRIME. In the end, DNMRIME achieved the lowest RMSE value compared to other MAs. The experimental data of DNMRIME exhibit clear peaks consistent with the actual data. The difference between the maximum and minimum RMSE of DNMRIME is small, resulting in its lowest Std.

On SDM, the performance of DNMRIME surpasses that of the other compared algorithms.

#### Experimental results of DNMRIME on DDM

**Fig. 16:**
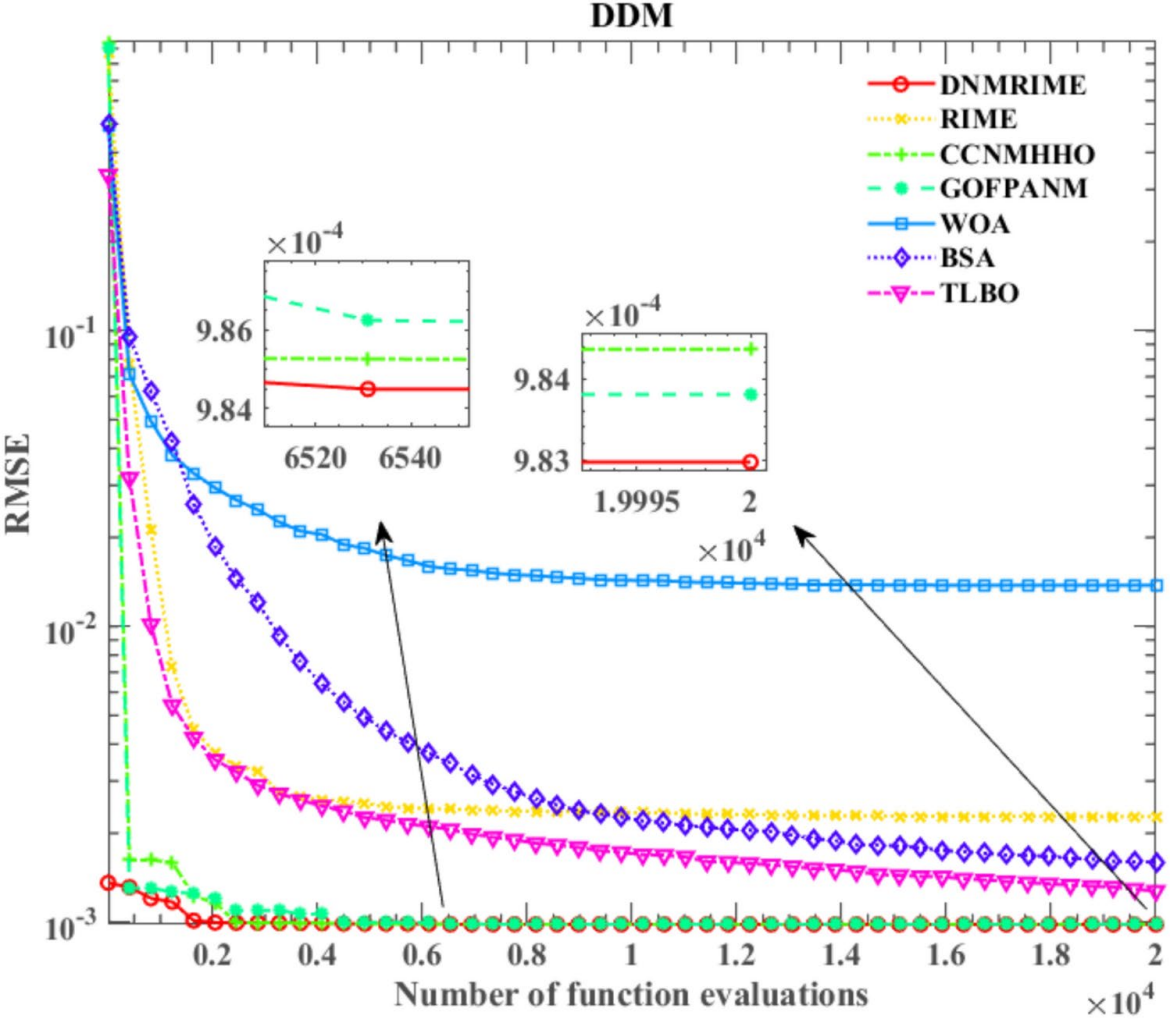
Convergence comparison of algorithms on DDM.

**Fig. 17:**
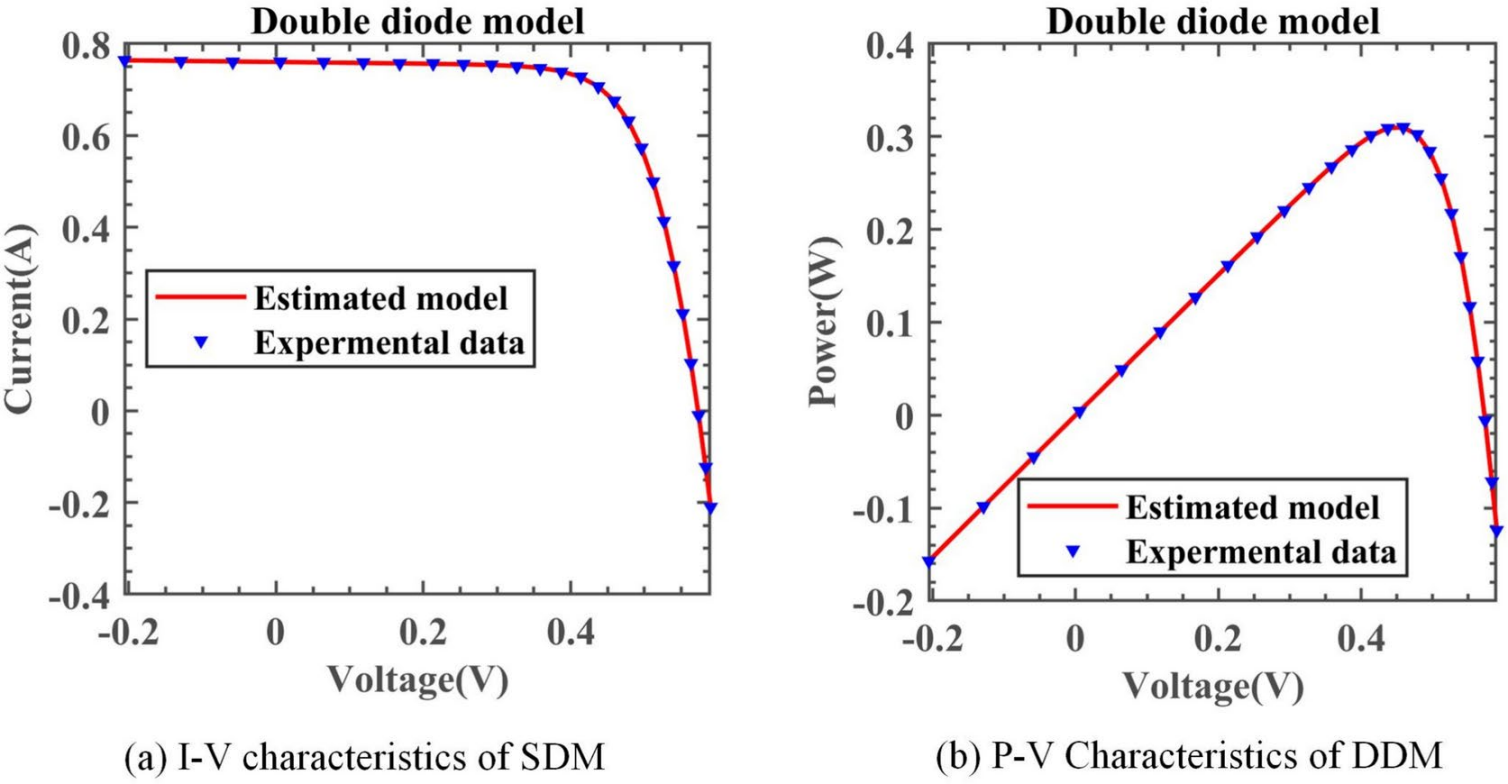
I–V and P–V characteristic curve of DNMRIME on DDM.

**Fig. 18:**
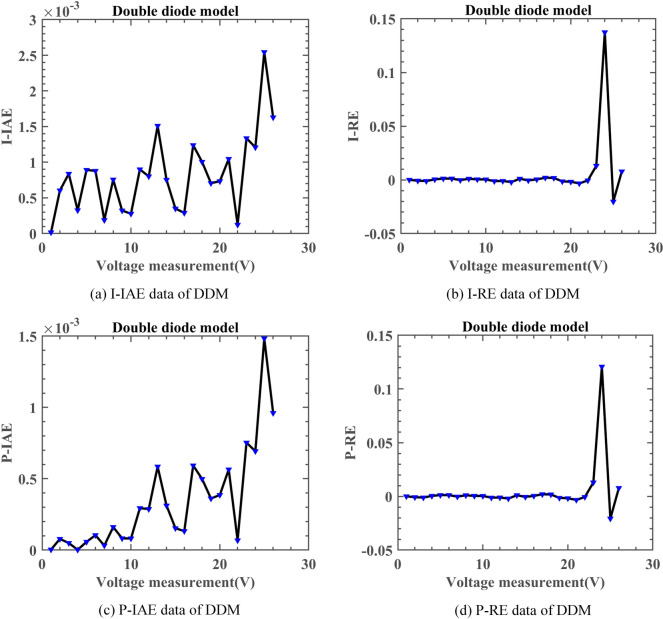
Error metrics of DNMRIME on DDM.

On DDM, Fig. 16 shows a convergence comparison of algorithms. Fig. 17 displays the I–V characteristic curves on DDM, while Fig. 18 illustrates the error indices of DNMRIME. Finally, Table 11 showcases the specific results on DDM, while Table 12 summarizes RMSE values.

**Table 11:** Parameters estimation results of DNMRIME with other algorithms on DDM.

Item	DNMRIME	RIME	CCNMHHO	GOFPANM	WOA	BSA	TLBO
${\text{I}}_{{{\text{ph}}}} {\text{(A)}}$	0.76078109	0.76073873	0.76078108	0.76078108	0.76162458	0.76066445	0.76075395
${\text{I}}_{{{\text{sd1}}}}$ (μA)	0.22594990	0.29448975	0.22597498	0.74936976	0.27741243	0.06720720	0.33225000
${\text{I}}_{{{\text{sd2}}}}$ (μA)	0.74956832	0.40255762	0.74934374	0.22597193	0.00000000	0.33941143	0.01077375
${\text{R}}_{{\text{s}}}$ (Ω)	3.67405249E − 02	3.59974861E − 02	3.67404235E − 02	3.67404378E − 02	3.67486284E − 02	3.59629945E − 02	3.62445709E − 02
${\text{R}}_{{{\text{sh}}}}$ (Ω)	55.48599213	58.20789437	55.48546717	55.48548112	41.34803895	58.14882331	55.01099801
$n_{1}$	1.45100785	1.47602357	1.45101705	2.00000000	1.46625977	1.77321765	1.48426220
$n_{2}$	1.99999999	1.93009347	2.00000000	1.45101592	1.62436839	1.48802160	1.79196736
$RMSE$	**9.8248E − 04**	1.0243E − 03	**9.8248E − 04**	**9.8248E − 04**	1.2118E − 03	1.0205E − 03	9.9147E − 04
Compare		+	=	=	+	+	+

Significant values are in bold.

**Table 12:** RMSE values of DNMRIME and other algorithms on DDM.

Item	$Max$	$Min$	$Mean$	$Std$
DNMRIME	9.86023449E − 04	9.82484852E − 04	**9.8296993325E − 04**	**9.21878E − 07**
RIME	4.04136804E − 03	1.02425801E − 03	2.2761976172E − 03	0.000909176
CCNMHHO	9.89130510E − 04	9.82484852E − 04	9.8436773877E − 04	2.1277E − 06
GOFPANM	9.86171797E − 04	9.82484852E − 04	9.8380696019E − 04	1.73767E − 06
WOA	4.74010845E − 02	1.21177304E − 03	1.3785341065E − 02	0.014313079
BSA	2.51079424E − 03	1.02047716E − 03	1.5942391080E − 03	0.000448579
TLBO	1.64218785E − 03	9.91470979E − 04	1.2747608685E − 03	0.000204125

Significant values are in bold.

Initially, DNMRIME, GOFPANM, and CCNMHHO performed well on DDM. DNMRIME quickly achieves lower RMSE values, reaching about 9.8296993325E − 04 at the final iteration. Furthermore, DNMRIME achieved the lowest RMSE after the iteration, indicating its optimal fitting effect in parameter estimation. DNMRIME demonstrated even greater accuracy in model fitting and has the lowest Std.

On DDM, DNMRIME exhibited the best performance.

#### Experimental results of DNMRIME on TDM

**Fig. 19:**
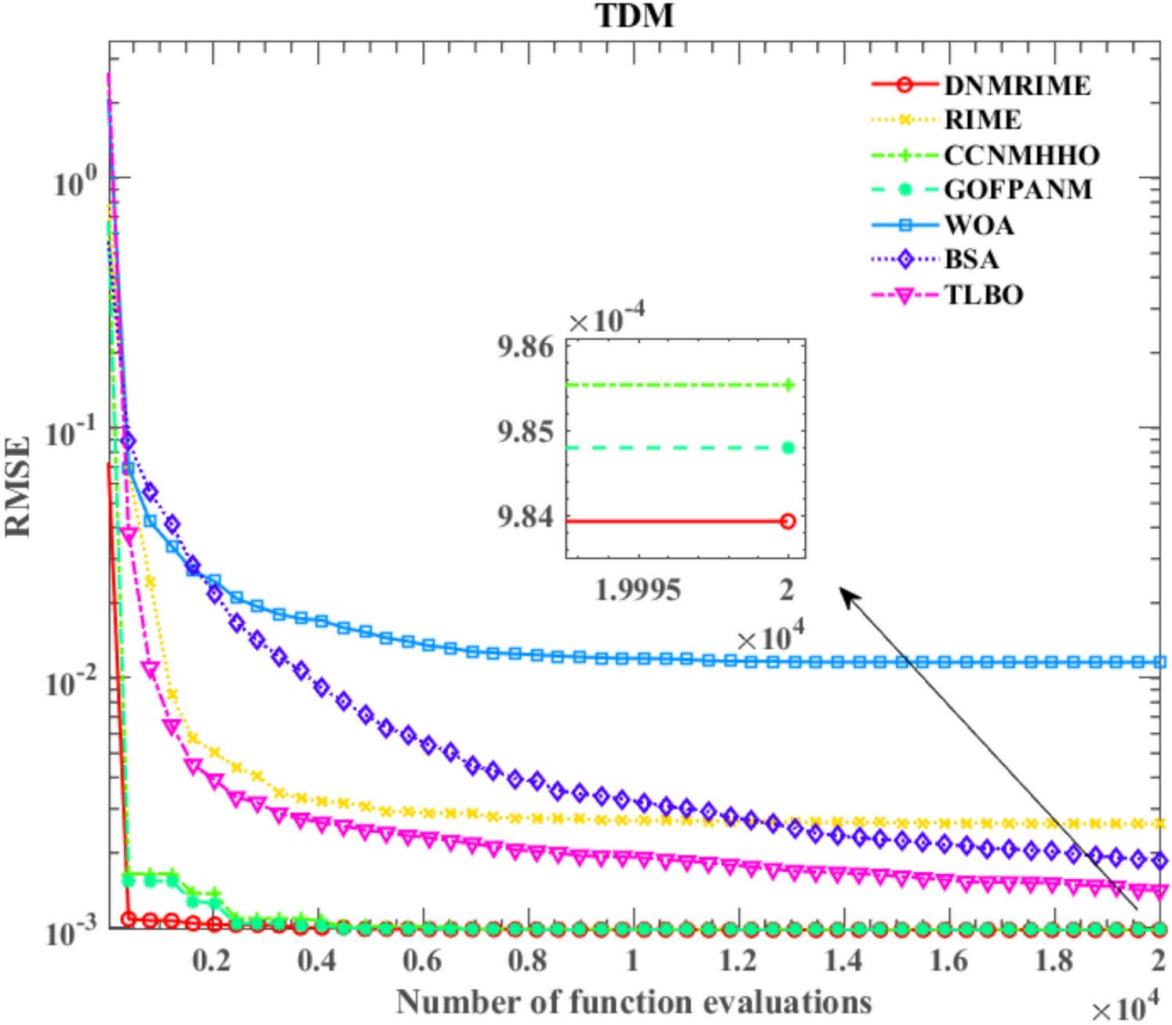
Convergence comparison of algorithms on TDM.

**Fig. 20:**
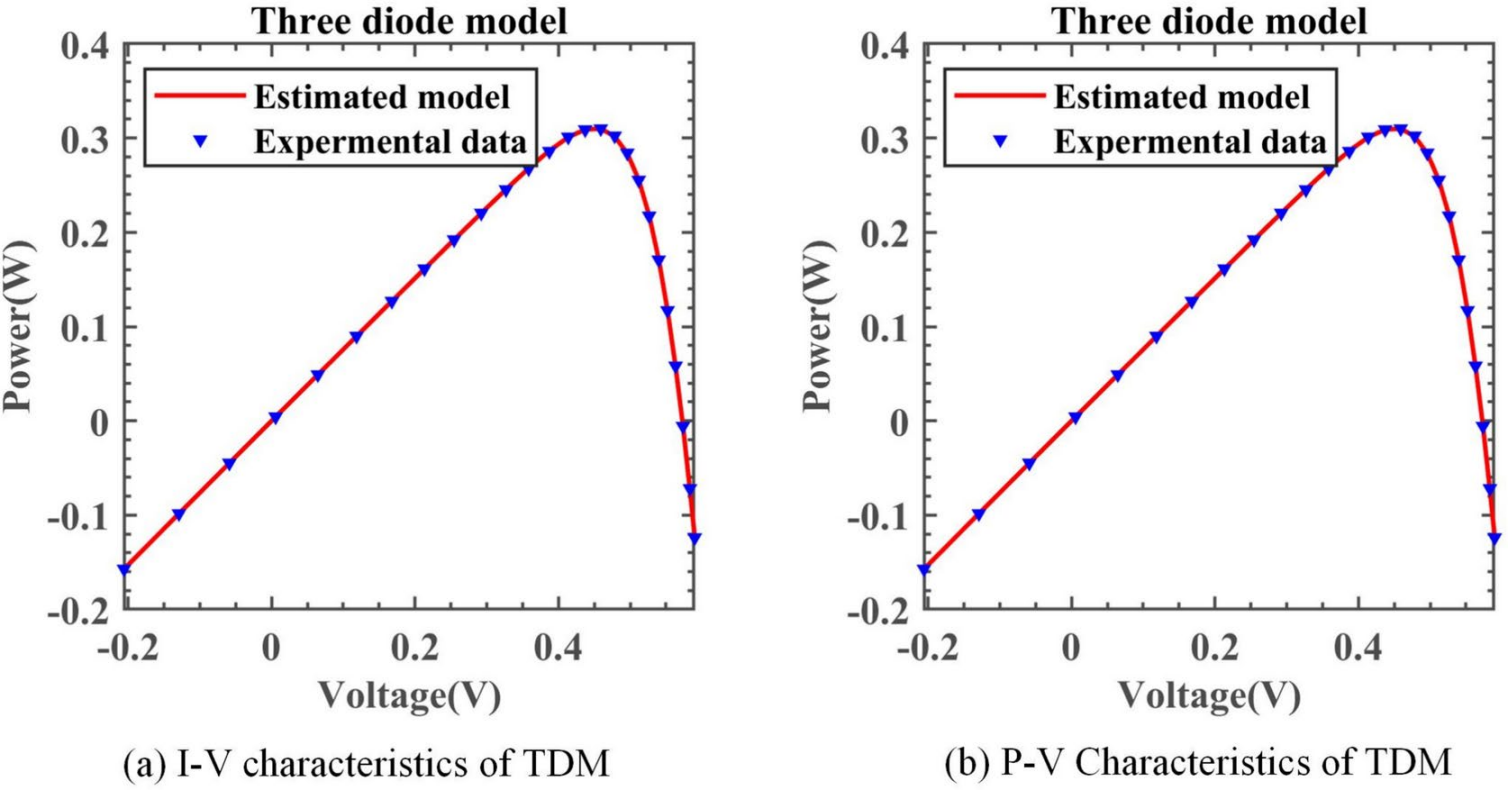
I–V and P–V characteristic curve of DNMRIME on TDM.

**Fig. 21:**
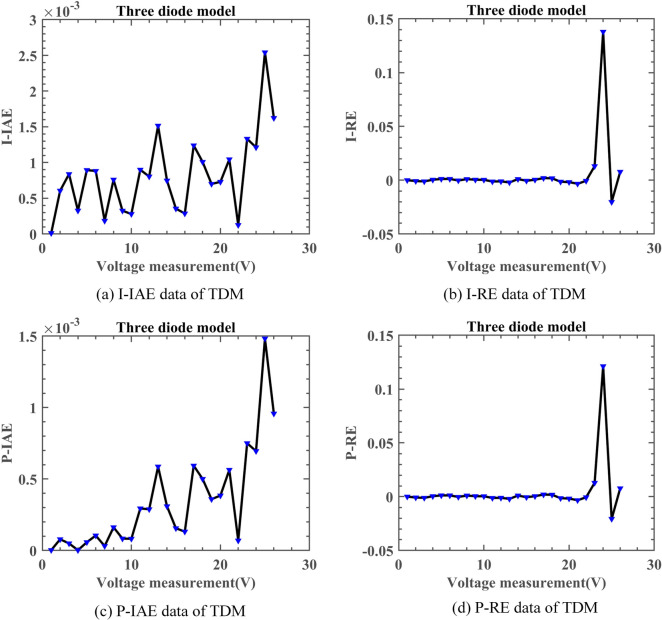
Error metrics of DNMRIME on TDM.

On TDM, Table 13 specifically describes the parameter extraction results of DNMRIME. Table 14 lists the RMSE values of each algorithm on TDM. Figure 19 shows the RMSE convergence curve of DNMRIME with other algorithms. Figure 20 shows two characteristic plots of DNMRIME on TDM, while Fig. 21 illustrates the error metrics.

**Table 13:** Parameters estimation results of DNMRIME with other algorithms on TDM.

Item	DNMRIME	RIME	CCNMHHO	GOFPANM	WOA	BSA	TLBO
${\text{I}}_{{{\text{ph}}}} {\text{(A)}}$	0.76078076	0.76149281	0.76078099	0.76078108	0.76122240	0.76042051	0.76082358
${\text{I}}_{{{\text{sd1}}}}$ (μA)	0.30526000	1.00000000	0.22695100	0.74709600	0.00550409	0.32258700	0.25695700
${\text{I}}_{{{\text{sd2}}}}$ (μA)	0.41339700	0.11393800	0.41507600	0.22597500	0.17917600	0.19564800	0.37826800
${\text{I}}_{{{\text{sd3}}}}$ (μA)	0.22961500	0.12208600	0.32599800	0.00224813	0.31226000	1.00000000	0.19676300
${\text{R}}_{{\text{s}}}$ (Ω)	3.67232450E − 02	3.82026620E − 02	3.67358170E − 02	3.67404280E − 02	3.50951330E − 02	3.57651690E − 02	3.68190870E − 02
${\text{R}}_{{{\text{sh}}}}$ (Ω)	55.41319592	45.88512600	55.46584733	55.48543589	55.80176047	73.20061637	54.77327294
$n_{1}$	1.99999963	1.93746201	1.45137729	2.00000000	1.65995724	1.89182840	1.84260742
$n_{2}$	1.99999947	1.39291748	1.99999999	1.45101692	1.65825935	1.44426571	1.92840553
$n_{3}$	1.45235392	2.00000000	2.00000000	2.00000000	1.49065251	1.97153659	1.44102726
$RMSE$	**9.8249E − 04**	1.1762E − 03	**9.8249E − 04**	**9.8249E − 04**	1.2041E − 03	1.2674E − 03	9.8488E − 04
Compare		+	=	=	=	+	+

Significant values are in bold.

**Table 14:** RMSE values of DNMRIME and other algorithms on TDM.

Item	$Max$	$Min$	$Mean$	$Std$
DNMRIME	9.88860941E − 04	9.82491890E − 04	**9.8393451046E − 04**	**1.7548E − 06**
RIME	3.88425922E − 03	1.17620112E − 03	2.6150797805E − 03	0.000863494
CCNMHHO	9.90074006E − 04	9.82485364E − 04	9.8553348389E − 04	2.27248E − 06
GOFPANM	9.92906638E − 04	9.82484852E − 04	9.8479539229E − 04	2.31492E − 06
WOA	4.18144832E − 02	1.20412332E − 03	1.1567324386E − 02	0.011292231
BSA	2.57322058E − 03	1.26739939E − 03	1.8679958308E − 03	0.000340022
TLBO	2.51122298E − 03	9.84879354E − 04	1.4130817952E − 03	0.000410508

Significant values are in bold.

Compared to RIME, WOA, BSA, and TLBO, DNMRIME demonstrates faster convergence speed in the curves. As for the mean RMSE value, DNMRIME reaches a value of 9.86E − 4, while CNMHHO and GOFPANM achieve approximately 9.85E − 4 and 9.86E − 4, respectively. DNMRIME achieves a lower final RMSE value than other algorithms on TDM. DNMRIME, CCNMHHO, and GOFPANM exhibit similar RMSE values around 9.8249E − 04. However, DNMRIME stands out with a much lower Std of 1.7548E − 06 compared to these two algorithms. With minimal Std, DNMRIME shows consistent RMSE fluctuations and a faster convergence rate. On TDM, DNMRIME demonstrates high accuracy in prediction.

On TDM, the performance of DNMRIME remains superior.

#### Experimental results of DNMRIME on PV

**Fig. 22:**
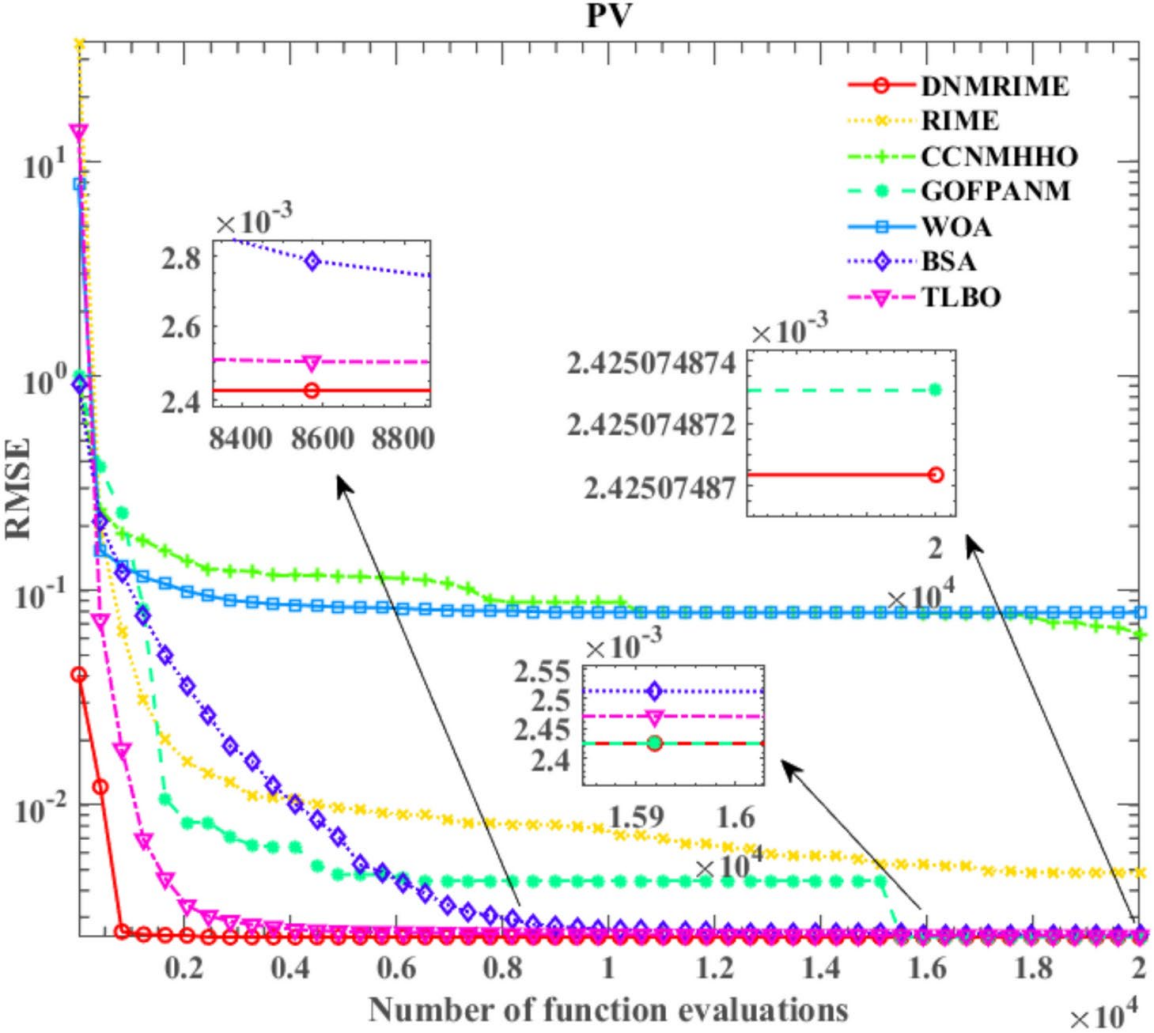
Convergence comparison of algorithms on PV.

**Fig. 23:**
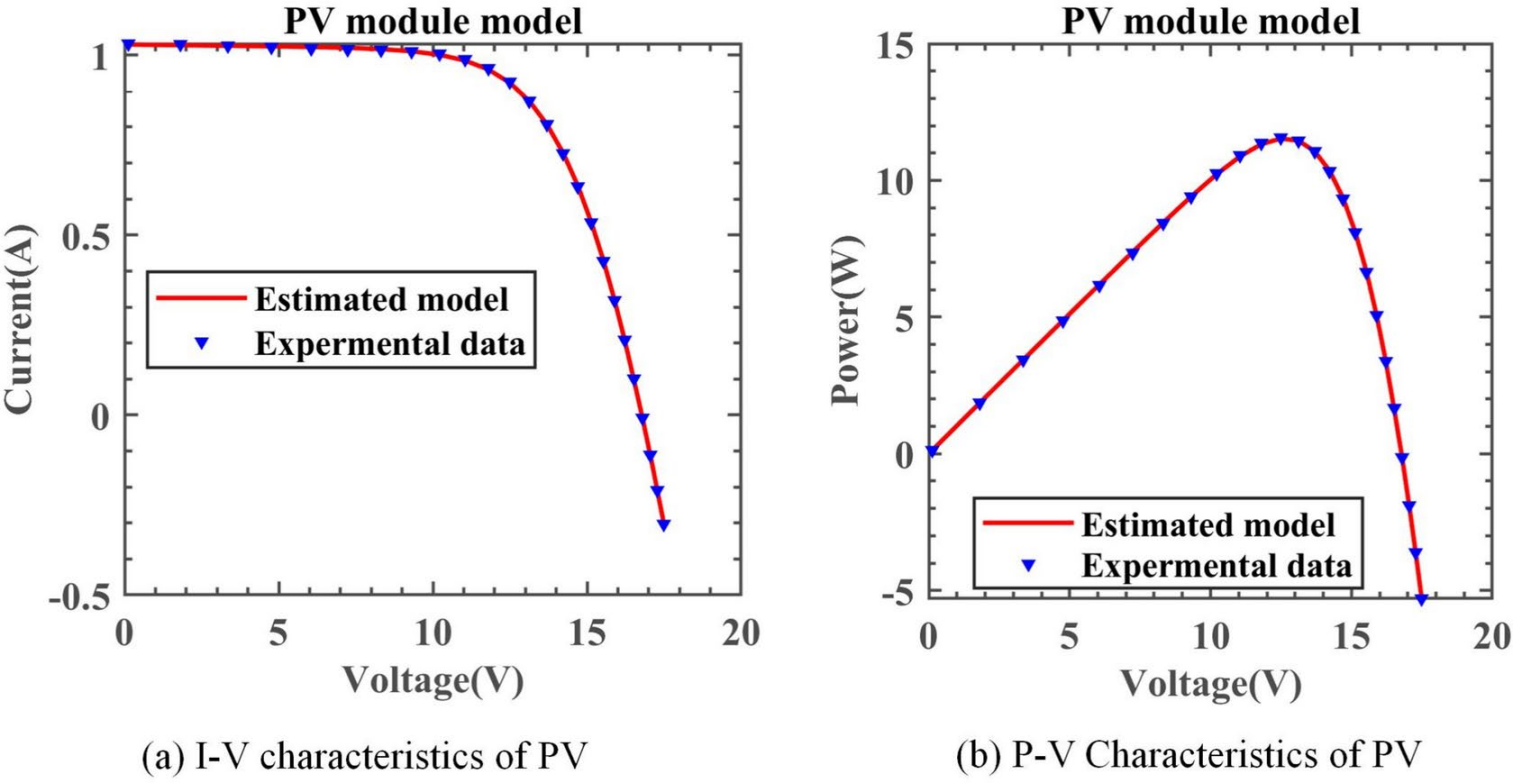
I–V and P–V characteristic curve of DNMRIME on PV.

**Fig. 24:**
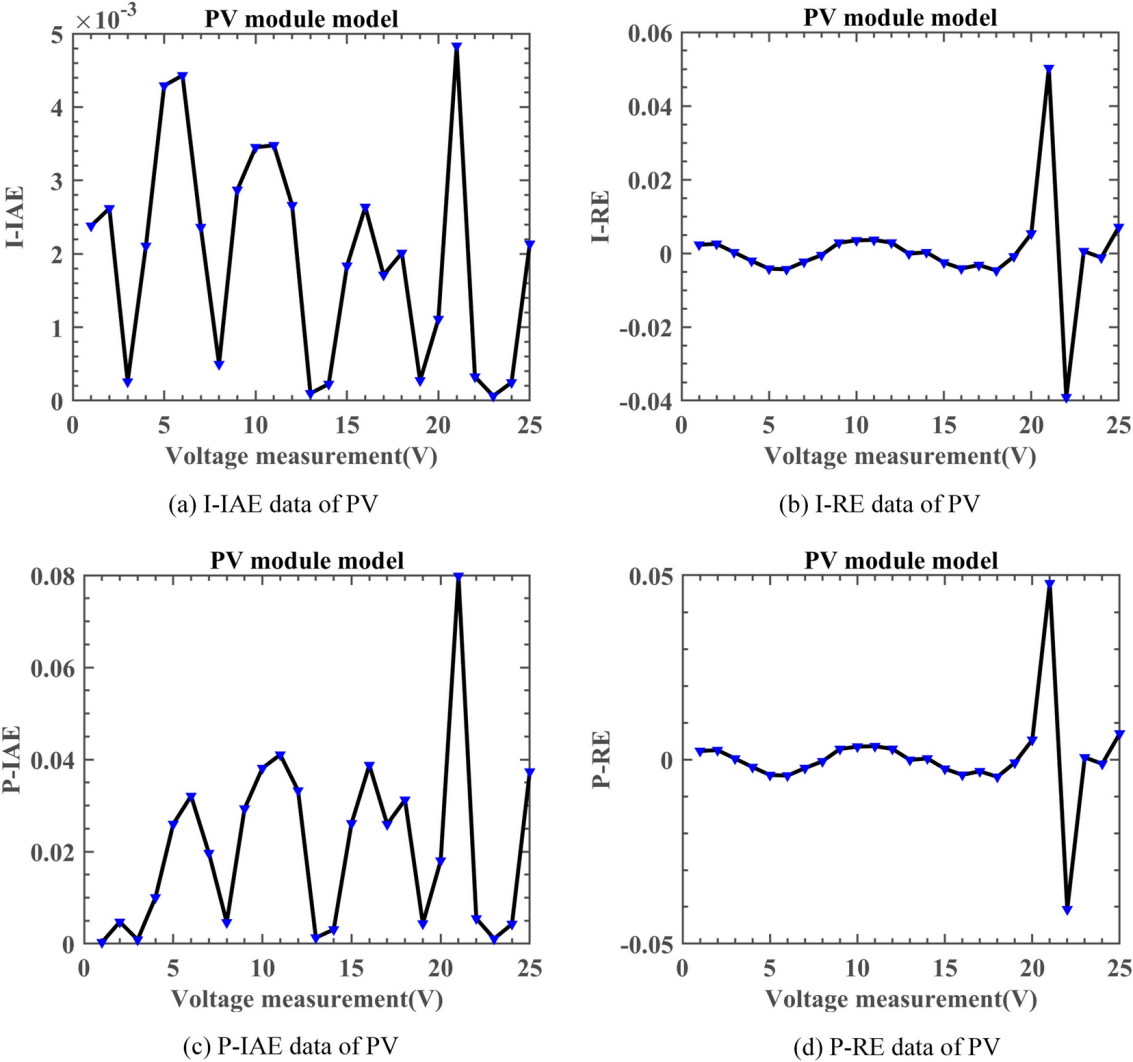
Error metrics of DNMRIME on PV.

On PV, Fig. 22 represents convergence curves, Fig. 23 depicts the I–V and P–V characteristic curves, Fig. 24 illustrates the error indicators. Moreover, Table 15 provides detailed results in parameter extraction, and Table 16 presents specific RMSE values.

**Table 15:** Parameters estimation results of DNMRIME with other algorithms on PV.

Item	DNMRIME	RIME	CCNMHHO	GOFPANM	WOA	BSA	TLBO
${\text{I}}_{{{\text{ph}}}} {\text{(A)}}$	1.03051417	1.03099664	1.03051439	1.03051430	1.02814553	1.03041899	1.03050649
${\text{I}}_{{{\text{sd}}}}$ (μA)	3.48233355	3.77739282	3.48223418	3.48226314	4.91771885	3.50804713	3.48500640
${\text{R}}_{{\text{s}}}$ (Ω)	1.20126916	1.19170008	1.20127166	1.20127100	1.16682214	1.20105866	1.20122084
${\text{R}}_{{{\text{sh}}}}$ (Ω)	982.00874023	978.25303671	981.96841662	981.98231868	1761.53244332	1000.49066433	984.45627076
$n$	48.64291200	48.95893121	48.64280381	48.64283511	49.99740397	48.67019614	48.64570564
$RMSE$	**2.4251E − 03**	2.4505E − 03	**2.4251E − 03**	**2.4251E − 03**	2.6146E − 03	2.4261E − 03	**2.4251E − 03**
Compare		+	=	=	=	+	=

Significant values are in bold.

**Table 16:** RMSE values of DNMRIME and other algorithms on PV.

Item	$Max$	$Min$	$Mean$	$Std$
DNMRIME	2.42507487E − 03	2.42507487E − 03	**2.4250748704E − 03**	**9.0046E − 13**
RIME	3.47130941E − 02	2.45048337E − 03	4.8501667168E − 03	0.007009457
CCNMHHO	4.84314324E − 01	2.42507487E − 03	6.2666049656E − 02	0.120533779
GOFPANM	2.42507488E − 03	2.42507487E − 03	2.4250748731E − 03	3.00095E − 12
WOA	2.75415742E − 01	2.61455796E − 03	7.9040153129E − 02	0.120023474
BSA	2.54370834E − 03	2.42611050E − 03	2.4855574418E − 03	3.09512E − 05
TLBO	2.72775948E − 03	2.42509970E − 03	2.4577300984E − 03	5.75831E − 05

Significant values are in bold.

Based on the experimental results, although CCNMHHO performed well on SDM, DDM, and TDM, it showed unsatisfactory performance on PV and performed worse than DNMRIME and RIME. On PV, TLBO outperforms the previous three models in convergence, yet its speed lags behind DNMRIME. Simultaneously, GOFPANM initially exhibits a slow convergence speed, even reaching a lower RMSE value only in the later evaluation stages. During the entire evaluation process, DNMRIME consistently maintains the lowest RMSE value. The final RMSE of DNMRIME is 2.42507487E − 03. While maintaining the lowest Std, DNMRIME boasts a remarkably low Std of 9.0046E − 13, significantly surpassing the Std of its competitors. Moreover, DNMRIME’s predictions are highly consistent with the actual data.

On PV, DNMRIME ranks first in performance.

#### CPU time cost assessment

**Fig. 25:**
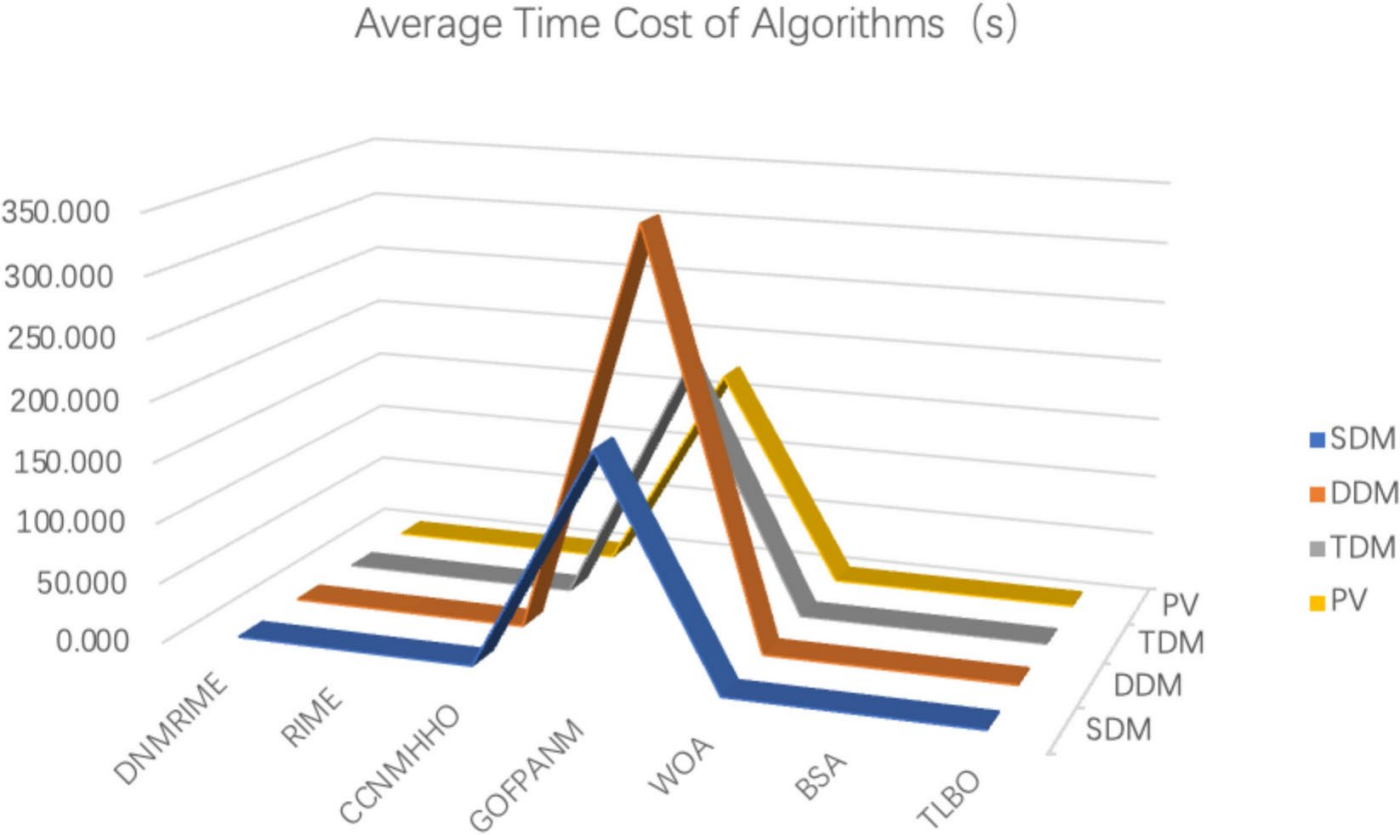
Average CPU expenditure time for each algorithm.

The average CPU expenditure time of the algorithms is one of the key metrics for evaluating their performance. As shown in Fig. 25 and Table 17, we can visually observe the differences in average time expenditure among different algorithms. Notably, the CPU time expenditure of the GOFOPANM is particularly significant, especially during the parameter extraction of the TDM, where it exceeds 400 s.

**Table 17:** The time cost of algorithm in photovoltaic model. (The unit of this table is seconds).

Item	SDM	DDM	TDM	PV
DNMRIME	0.951	1.192	0.540	0.766
RIME	0.840	1.184	0.369	0.666
CCNMHHO	0.967	1.216	0.425	0.748
GOFPANM	185.427	341.267	199.221	172.905
WOA	0.788	0.927	0.406	0.727
BSA	0.874	0.991	0.404	0.769
TLBO	0.967	1.034	0.472	0.818

It is worth noting that the average CPU expenditure time of DNMRIME is not the shortest. Although its time overhead may not be as compact as other algorithms except for GOFOPANM, it demonstrates excellent accuracy and stability in photovoltaic model parameter extraction, completing tasks with relatively low CPU expenditure time under various model conditions.

### Experimental results for manufacturer solar cell models

In this section of the paper, we conduct simulation experiments on solar panels of three different models from manufacturers: KC200GT, ST40, and SM55. The simulation experiments systematically explore their operational efficiency and output power characteristics under varying light conditions, temperature environments, and load requirements.

#### Results of different irradiation and constant temperature

**Fig. 26:**
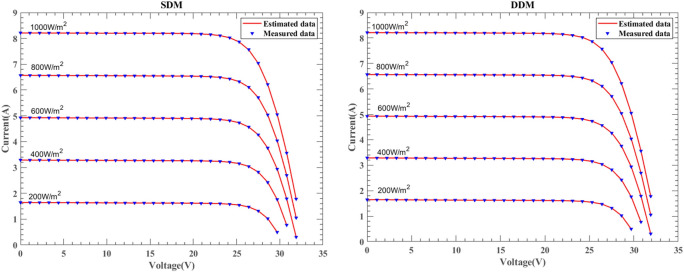
The I–V characteristic curves of KC200GT under with different irradiance.

**Fig. 27:**
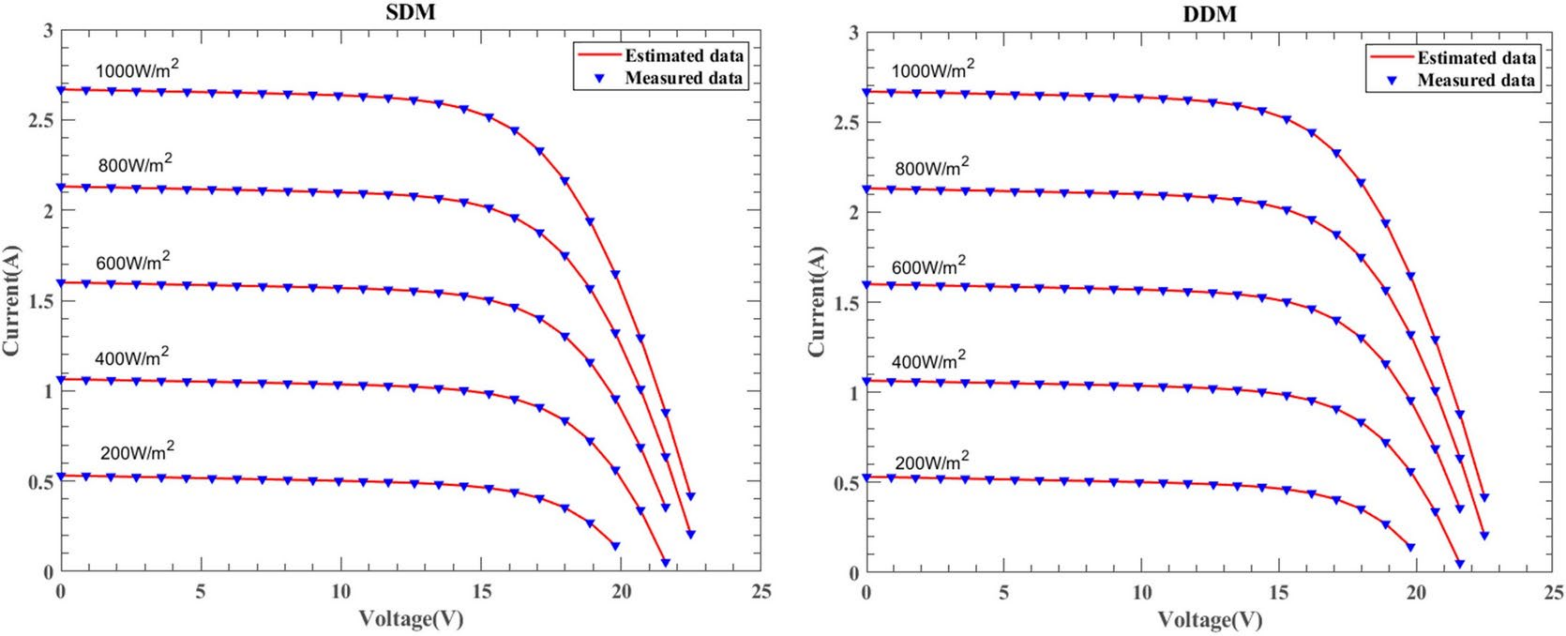
The I–V characteristic curves of ST40 under different irradiance.

**Fig. 28:**
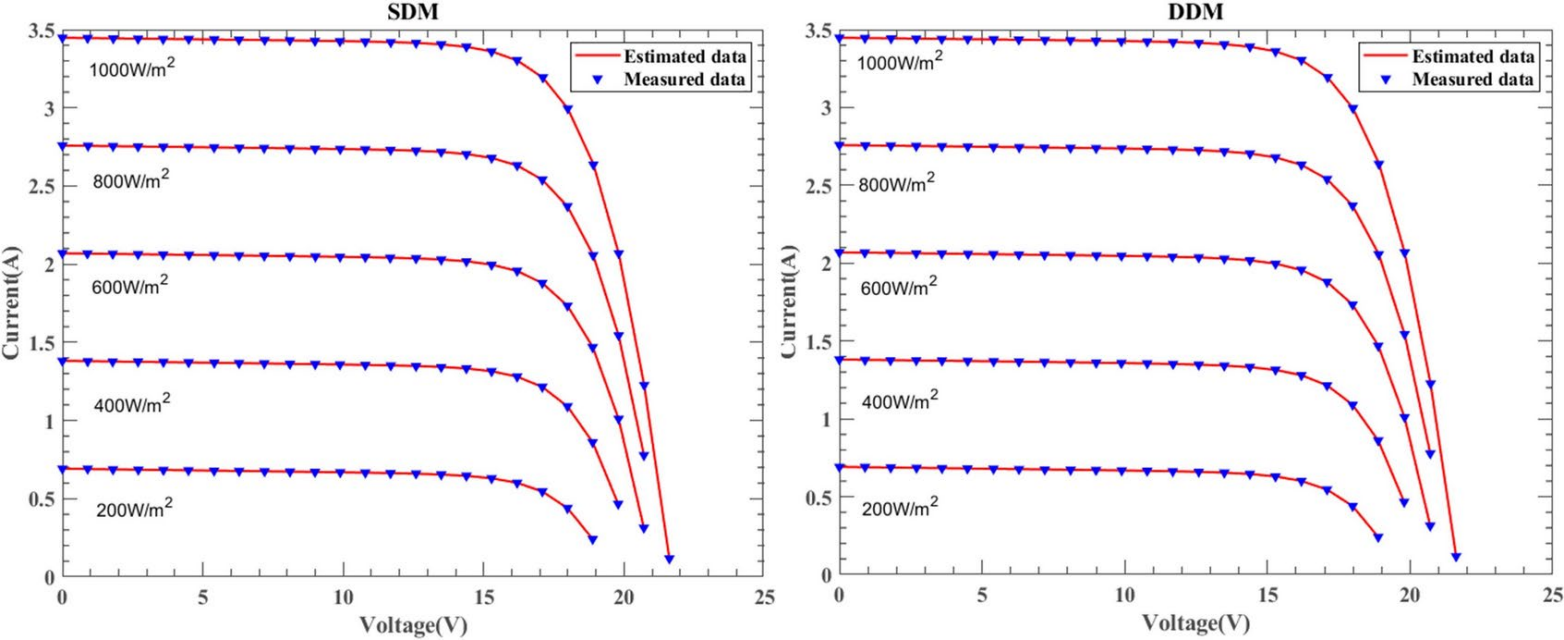
The I–V characteristic curves of SM55 under different irradiance.

This section presents the simulation results of SDM and DDM solar cells under the KC200GT, ST40, and SM55 models. Figures 26, 27 and 28 illustrate the I–V characteristics experiment results under constant temperature conditions of 25 ℃ for irradiance levels of $1000W/{\text{m}}^{2}$, $800W/{\text{m}}^{2}$, $600W/{\text{m}}^{2}$, $400W/{\text{m}}^{2}$, and $200W/{\text{m}}^{2}$, respectively. The consistency between the experimental results and real data is significant, validating the accuracy of DNMRIME in predicting solar cell performance.

#### Results of different temperature and constant irradiation

**Fig. 29:**
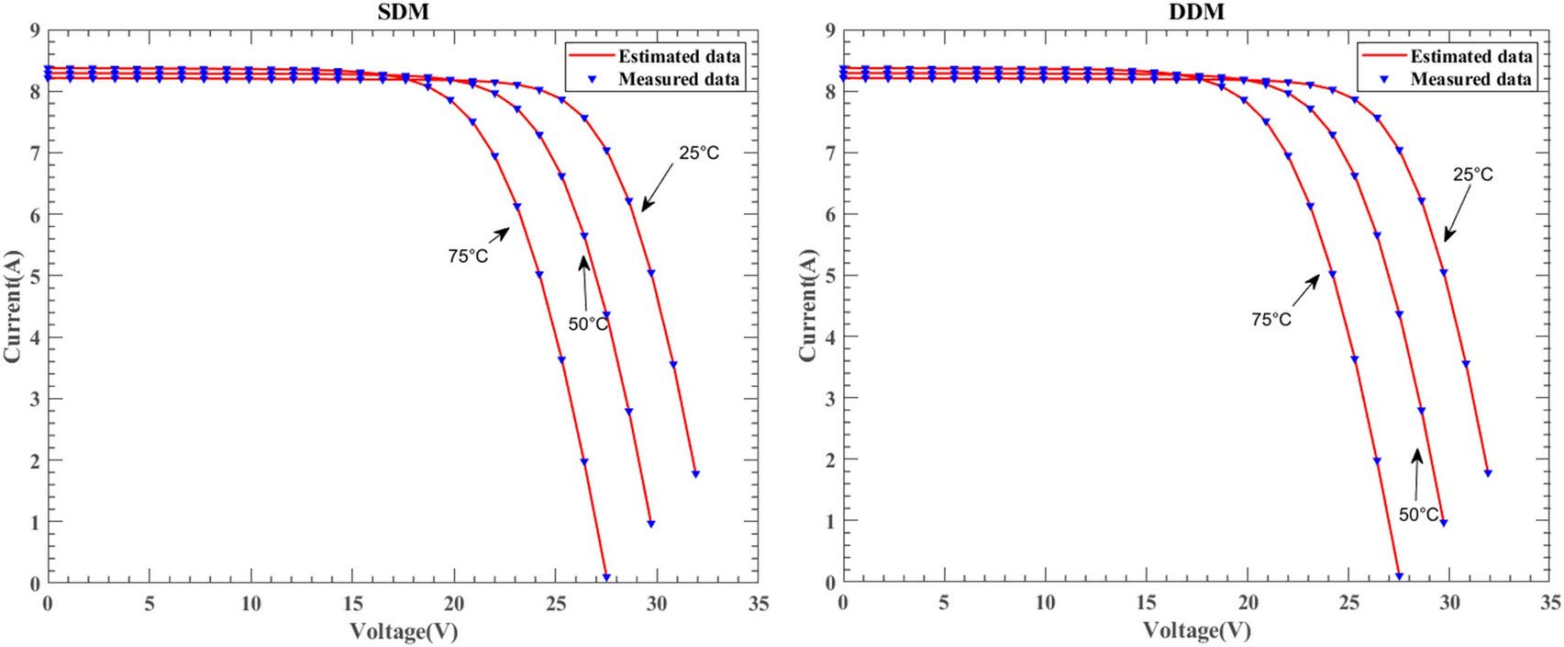
The I–V characteristic curves of KC200GT under different temperature.

**Fig. 30:**
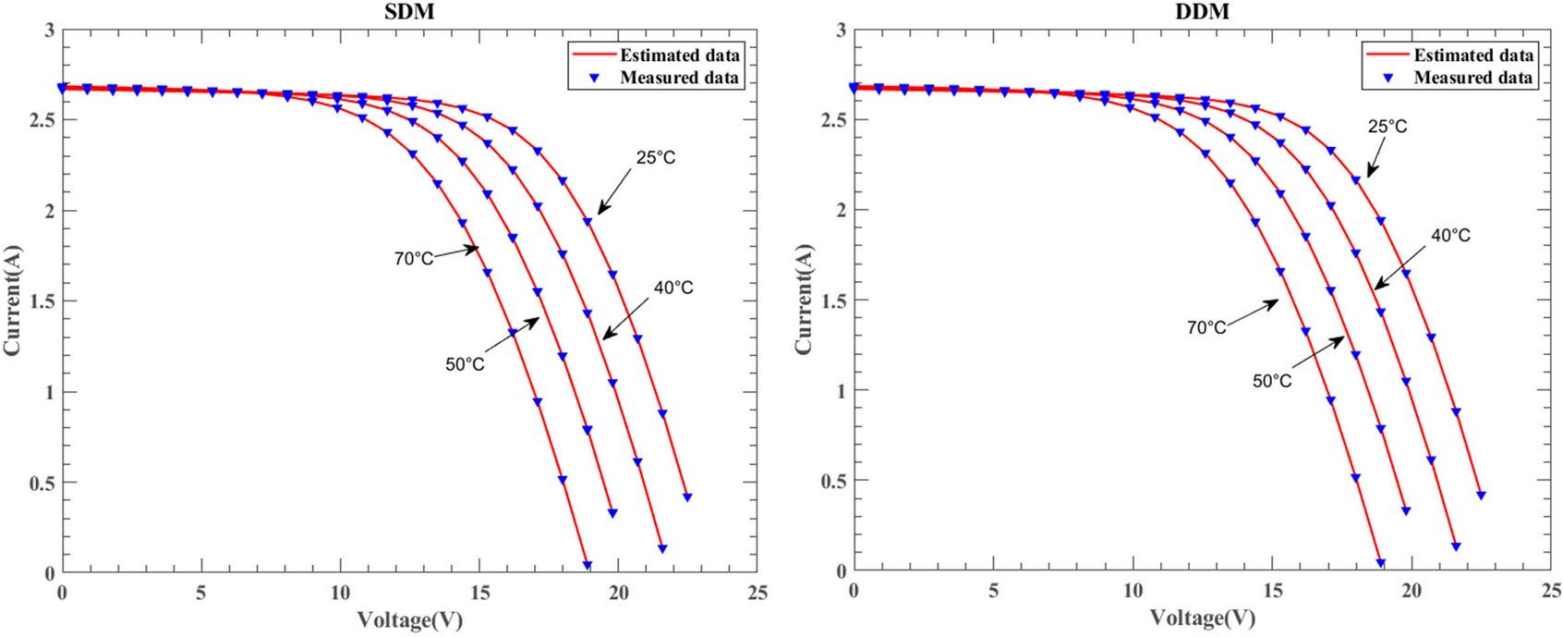
The I–V characteristic curves of ST40 under different temperature.

**Fig. 31:**
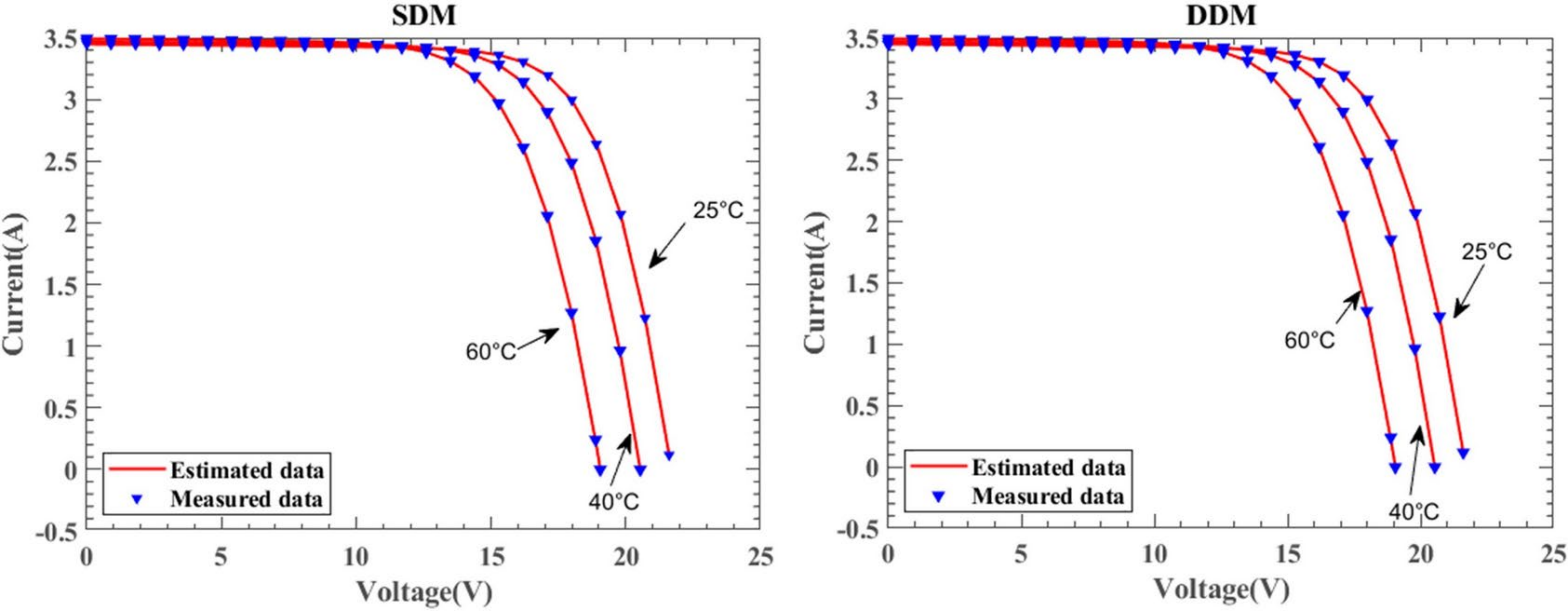
The I–V characteristic curves of SM55 under different temperature.

Figure 29 illustrates the I–V characteristic curves of SDM and DDM solar cells under the KC200GT model, obtained from simulation experiments conducted at constant irradiance conditions of 25 °C, 50 °C, and 75 °C temperatures. Fig. 30 depicts the I–V characteristic curves of SDM and DDM solar cells under the ST40 model, where experiments were conducted at a constant irradiance of $1000W/{\text{m}}^{2}$ and temperatures of 25 °C, 40 °C, 50 °C, and 75 °C. Additionally, Fig. 31 displays the I-V characteristic curves of SDM and DDM solar cells under the SM55 model, with experiments conducted at constant irradiance conditions and temperatures of 25 °C, 40 °C, and 60 °C. The actual data is highly consistent with the data estimated by DNMRIME. These experiments validate the effectiveness of DNMRIME.

## Discussion on the results

In our research, we have observed the remarkable performance of a novel RIME algorithm in extracting photovoltaic parameters. This paper introduces a new dynamic multi-dimensional random mechanism, a dynamic random search strategy enhancing convergence accuracy across multiple dimensions. To achieve a more accurate extraction of photovoltaic model parameters, we have integrated this mechanism with the NMs and enhanced RIME, resulting in the development of the DNMRIME.

As for the CEC 2017 evaluation, we found that the DNMRIME significantly outperforms other algorithms regarding convergence speed. The exploration capability of DNMRIME increased by approximately 25.02991% in F1, 27.56922% in F4, 18.68052% in F7, 22.75426% in F25, and 27.3841% in F28 compared to RIME. Therefore, DNMRIME has more population diversity to find better solutions than RIME. Compared with well-known MAs, the results of the WSRT show that DNMRIME ranks first and has demonstrated excellent performance on hybrid and composite functions.

As for a series of simulation experiments in photovoltaic parameter extraction, we have validated the superiority of DNMRIME in this field. In the cases of SDM, DDM, TDM, and PV, DNMRIME has the lowest Std, with values of 2.00339E − 12, 9.21878E − 07, 1.7548E − 06, and 9.0046E − 13, respectively. Meanwhile, compared to CCNMHHO and GOFPANM, DNMRIME demonstrates faster convergence speed. This indicates that DNMRIME possesses efficiency, stability, and robustness. DNMRIME also consistently displays its advantages under various environmental conditions. The extracted parameters exhibit high degrees of consistency and accuracy compared to the actual parameters.

These results show the significance and practical utility of DNMRIME in photovoltaic parameter extraction.

## Conclusions and future directions

This work proposes an improved version of RIME, named DNMRIME, by including the dynamic multi-dimensional random mechanism (DMRM) in conjunction with the Nelder–Mead simplex (NMs). Through random non-periodic convergence, DMRM increases RIME’s convergence accuracy. NMs speed up convergence, allowing DNMRIME to avoid local optima and outperform it on hybrid and composite functions. A qualitative analysis and ablation test were carried out on CEC 2017 in order to assess DNMRIME’s efficiency. In order to confirm its efficacy, it compared to 14 other well-known MAs, including several advanced techniques. DNMRIME was used for obtaining parameters for SDM, DDM, TDM, and PV as well. In conclusion, this study significantly proved the performance of DNMRIME for parameter identification in solar models. Not only does the algorithm exhibit better performance in terms of accuracy and convergence speed, but it also demonstrates robustness across different types of photovoltaic models.

Looking ahead, several potential research directions emerge. Firstly, further exploring the interaction between the DMRM and the NMs may lead to more sophisticated hybrid optimization algorithms tailored to specific problems. Secondly, in photovoltaic parameter extraction, we have noticed the Lambert $W$ function^97^ as a promising method for improved accuracy and plan to apply DNMRIME to it in the future. Lastly, applying the improved algorithm to other domains, such as machine learning or signal processing, may reveal its broader utility and potential.

The further development and application prospects of DNMRIME remain promising and warrant further exploration.

## Supplementary Information

Supplementary Information.

## Data Availability

Data is provided within the manuscript or supplementary information files.

## Abbreviations

RIME: Rime optimization algorithm
NMs: Nelder–Mead simplex
SDM: Single diode model
DDM: Double diode model
TDM: Three diode model
PV: Photovoltaic
$MaxFEs$: Maximum number of iterations
$N$: Population size or problem scale
SM55: Mono-crystalline PV module
ST40: Thin-film PV module
KC200GT: Multicrystal photovoltaic module
$$I_{L}$$: Output current
$I_{ph}$: Photo-generated current
$$I_{d} ,I_{d1} ,I_{d2} ,I_{d3}$$: Diode current
$I_{sh}$: Shunt resistor current
$I_{sd} ,I_{sd1} ,I_{sd2} ,I_{sd3}$: Reverse saturated current
$$dim$$: Problem dimension
$$R_{S}$$: Series resistor
$$R_{sh}$$: Parallel resistor
*n*, *n*_1_, *n*_2_, *n*_3_: Diode ideality factor
WSRT: Wilcoxon signed-rank test
$q$: The charge of the electron
*k*: Boltzmann constant
T: Kelvin temperature
RMSE: Root mean square error
$FEs$: The number of the current iteration
$R_{best,j}$: The $j$-th dimension of the best rime
$$UB$$: Upper boundary
$$LB$$: Lower boundary
$N_{P}$: Number of parallel solar cells
$N_{S}$: Number of series solar cells
$R_{ij}^{new}$: The new position of the particle
$\cos \theta$: The Particle movement direction
$\beta$: The environmental factor
$r_{i}$: The $i$-th random number in $\left[ {0,1} \right]$
$F^{normr} (S_{i} )$: The normalized value

## Declarations

### Declaration of AI and AI-assisted technologies in the writing process

During the preparation of this work, the author(s) used ChatGPT to improve and proofread the English of the paper. After using this tool/service, the author(s) reviewed and edited the content as needed and take(s) full responsibility for the content of the pblication.
