# Supplementary material for: RIME optimization with dynamic multi-dimensional random mechanism and Nelder–Mead simplex for photovoltaic parameter estimation
Source: Sci Rep. 2025 Jul 1;15:20951. doi: 10.1038/s41598-025-99105-6 (PMC12219663; doi:10.1038/s41598-025-99105-6)
Supplement: Supplementary file 1 — Supplementary Information. [file 41598_2025_99105_MOESM1_ESM.docx]

# Appendix

**Appendix Table 1**. Results of DNMRIME for ablation study on CEC 2017.

|  | **F1** |  |  | **F2** |  |  | **F3** |  |
| --- | --- | --- | --- | --- | --- | --- | --- | --- |
|  | Average | Stdv |  | Average | Stdv |  | Average | Stdv |
| DNMRIME | **1.0269E+02** | 3.4778E+00 |  | 6.1545E+06 | 2.1312E+07 |  | 3.6667E+02 | 9.3415E+01 |
| DRIME | 1.8820E+04 | 6.7804E+03 |  | 3.0201E+06 | 9.2660E+06 |  | 3.2283E+02 | 8.5286E+00 |
| NMRIME | 1.3323E+02 | 1.3778E+02 |  | 2.0319E+08 | 4.2132E+08 |  | 3.7733E+02 | 1.8894E+02 |
| RIME | 8.3821E+03 | 1.1518E+04 |  | **1.4761E+04** | 3.9138E+04 |  | **3.0140E+02** | 5.0629E-01 |
|  | **F4** |  |  | **F5** |  |  | **F6** |  |
|  | Average | Stdv |  | Average | Stdv |  | Average | Stdv |
| DNMRIME | 4.0015E+02 | 7.2879E-01 |  | 6.0590E+02 | 1.9104E+01 |  | 6.3269E+02 | 4.1300E+00 |
| DRIME | 4.9781E+02 | 3.5381E+01 |  | 5.9806E+02 | 1.9039E+01 |  | 6.0104E+02 | 1.2468E+00 |
| NMRIME | **4.0000E+02** | 5.3995E-03 |  | 6.4982E+02 | 3.7028E+01 |  | 6.4019E+02 | 8.8275E+00 |
| RIME | 4.9605E+02 | 4.2836E+01 |  | **5.8735E+02** | 2.6988E+01 |  | **6.0032E+02** | 3.0033E-01 |
|  | **F7** |  |  | **F8** |  |  | **F9** |  |
|  | Average | Stdv |  | Average | Stdv |  | Average | Stdv |
| DNMRIME | **7.8962E+02** | 1.5617E+01 |  | 9.3112E+02 | 2.6603E+01 |  | 3.8718E+03 | 6.0236E+02 |
| DRIME | 8.2442E+02 | 1.7065E+01 |  | 8.9653E+02 | 2.6248E+01 |  | 2.9444E+03 | 1.4239E+03 |
| NMRIME | 8.0012E+02 | 2.2767E+01 |  | 9.6522E+02 | 3.2212E+01 |  | 6.4788E+03 | 1.5106E+03 |
| RIME | 8.1877E+02 | 2.0351E+01 |  | **8.7445E+02** | 1.8358E+01 |  | **1.3690E+03** | 6.3996E+02 |
|  | **F10** |  |  | **F11** |  |  | **F12** |  |
|  | Average | Stdv |  | Average | Stdv |  | Average | Stdv |
| DNMRIME | 3.6189E+03 | 3.8169E+02 |  | **1.2063E+03** | 3.7854E+01 |  | **3.2032E+03** | 6.7221E+02 |
| DRIME | 3.7260E+03 | 6.2469E+02 |  | 1.2543E+03 | 4.4880E+01 |  | 9.9873E+06 | 4.8530E+06 |
| NMRIME | 3.7863E+03 | 4.4557E+02 |  | 1.2175E+03 | 4.5395E+01 |  | 3.2182E+03 | 5.4706E+02 |
| RIME | **3.3537E+03** | 4.7733E+02 |  | 1.2381E+03 | 5.4721E+01 |  | 4.1166E+06 | 2.9649E+06 |
|  | **F13** |  |  | **F14** |  |  | **F15** |  |
|  | Average | Stdv |  | Average | Stdv |  | Average | Stdv |
| DNMRIME | **1.5398E+03** | **2.1497E+02** |  | 1.5185E+03 | 4.1261E+01 |  | **1.5465E+03** | 1.5710E+01 |
| DRIME | 4.3695E+03 | 2.8375E+03 |  | 1.6473E+03 | 9.5439E+01 |  | 3.5018E+03 | 1.9454E+03 |
| NMRIME | 1.5433E+03 | 1.6542E+02 |  | 1.5245E+03 | 4.6695E+01 |  | 1.6104E+03 | 3.0460E+01 |
| RIME | 3.8470E+03 | 2.6293E+03 |  | **1.5119E+03** | 3.7786E+01 |  | 6.4664E+03 | 5.4593E+03 |
|  | **F16** |  |  | **F17** |  |  | **F18** |  |
|  | Average | Stdv |  | Average | Stdv |  | Average | Stdv |
| DNMRIME | 2.2395E+03 | 2.2432E+02 |  | 2.1110E+03 | 1.5547E+02 |  | **2.1047E+03** | 2.1216E+02 |
| DRIME | **2.2021E+03** | 2.3504E+02 |  | 1.9804E+03 | 1.1867E+02 |  | 1.1542E+05 | 6.5261E+04 |
| NMRIME | 2.2776E+03 | 2.0502E+02 |  | 2.1353E+03 | 1.3176E+02 |  | 2.2001E+03 | 2.8774E+02 |
| RIME | 2.3480E+03 | 2.4011E+02 |  | **1.9770E+03** | 1.1169E+02 |  | 1.1715E+05 | 8.3789E+04 |
|  | **F19** |  |  | **F20** |  |  | **F21** |  |
|  | Average | Stdv |  | Average | Stdv |  | Average | Stdv |
| DNMRIME | **1.9528E+03** | 2.5440E+01 |  | 2.5049E+03 | 1.3913E+02 |  | 2.1003E+03 | 1.0876E+00 |
| DRIME | 4.5307E+03 | 3.2129E+03 |  | 2.3374E+03 | 1.4901E+02 |  | 2.2048E+03 | 3.0033E+01 |
| NMRIME | 1.9799E+03 | 2.8422E+01 |  | 2.4692E+03 | 1.5964E+02 |  | **2.1000E+03** | 1.4849E-01 |
| RIME | 8.6879E+03 | 5.8206E+03 |  | **2.2943E+03** | 1.2502E+02 |  | 2.1895E+03 | 3.3944E+01 |
|  | **F22** |  |  | **F23** |  |  | **F24** |  |
|  | Average | Stdv |  | Average | Stdv |  | Average | Stdv |
| DNMRIME | 2.3215E+03 | 2.2594E+01 |  | **2.5000E+03** | 2.0195E-07 |  | **2.5967E+03** | 1.8257E+01 |
| DRIME | 2.3157E+03 | 2.1050E+01 |  | **2.5000E+03** | 0.0000E+00 |  | 2.6000E+03 | 0.0000E+00 |
| NMRIME | 2.3733E+03 | 4.3494E+01 |  | 3.4460E+03 | 3.3475E+02 |  | 2.8777E+03 | 3.8160E+02 |
| RIME | **2.2749E+03** | 1.8617E+01 |  | 2.8786E+03 | 2.5614E+01 |  | 3.1724E+03 | 3.4999E+02 |
|  | **F25** |  |  | **F26** |  |  | **F27** |  |
|  | Average | Stdv |  | Average | Stdv |  | Average | Stdv |
| DNMRIME | **2.7000E+03** | 4.3058E-13 |  | **2.8000E+03** | 2.7389E-12 |  | 2.9100E+03 | 5.4774E+01 |
| DRIME | **2.7000E+03** | 0.0000E+00 |  | **2.8000E+03** | 0.0000E+00 |  | **2.9000E+03** | 0.0000E+00 |
| NMRIME | 2.8980E+03 | 1.0281E+00 |  | 4.4801E+03 | 1.6458E+03 |  | 4.0021E+03 | 1.4660E+02 |
| RIME | 2.9763E+03 | 6.0929E+01 |  | 5.1439E+03 | 9.6797E+02 |  | 3.5879E+03 | 1.2150E+02 |
|  | **F28** |  |  | **F29** |  |  | **F30** |  |
|  | Average | Stdv |  | Average | Stdv |  | Average | Stdv |
| DNMRIME | **3.0000E+03** | 1.2831E-08 |  | **3.1000E+03** | 2.1414E-08 |  | 3.2619E+03 | 2.3587E+02 |
| DRIME | **3.0000E+03** | 0.0000E+00 |  | **3.1000E+03** | 0.0000E+00 |  | **3.2000E+03** | 0.0000E+00 |
| NMRIME | 3.1488E+03 | 3.7288E+01 |  | 3.7233E+03 | 1.5453E+02 |  | 5.3616E+03 | 2.3437E+03 |
| RIME | 3.4160E+03 | 4.8362E+02 |  | 3.5966E+03 | 1.7056E+02 |  | 3.3365E+04 | 1.6955E+04 |

**Appendix Table 2**. Results of DNMRIME and well-known MAs on CEC 2017 (F1-F9).

|  | **F1** |  |  | **F2­­** |  |  | **F3** |  |
| --- | --- | --- | --- | --- | --- | --- | --- | --- |
|  | Average | Stdv |  | Average | Stdv |  | Average | Stdv |
| DNMRIME | 1.0416E+02 | 9.7673E+00 |  | 4.1645E+06 | 1.2232E+07 |  | 4.0464E+02 | 2.4863E+02 |
| RIME | 7.2166E+03 | 3.8092E+03 |  | **1.5281E+04** | 1.8172E+04 |  | 3.0151E+02 | 6.0161E-01 |
| DE | 2.6371E+02 | 5.8304E+02 |  | 4.0634E+25 | 1.5609E+26 |  | 2.1688E+04 | 5.2699E+03 |
| PSO | 1.4229E+08 | 1.6860E+07 |  | 2.4835E+12 | 3.1193E+12 |  | 6.2006E+02 | 4.0789E+01 |
| WOA | 5.2430E+06 | 7.2599E+06 |  | 6.9128E+28 | 3.4308E+29 |  | 1.2513E+04 | 3.5143E+03 |
| HHO | 1.2938E+07 | 2.7678E+06 |  | 1.4645E+12 | 4.1392E+12 |  | 2.5551E+03 | 1.0737E+03 |
| WSO | 6.7898E+10 | 2.0572E+10 |  | 1.4524E+40 | 7.0883E+40 |  | 8.2453E+04 | 2.7738E+04 |
| AHA | 2.5358E+03 | 2.9174E+03 |  | 1.3965E+23 | 7.6481E+23 |  | 3.1294E+02 | 2.5189E+01 |
| LSHADE | **1.0000E+02** | 2.4755E-14 |  | 3.1443E+14 | 1.4260E+15 |  | 1.3114E+04 | 2.6250E+04 |
| LSHADE_cnEpSi | **1.0000E+02** | 1.8562E-09 |  | 3.4450E+20 | 1.6495E+21 |  | **3.0000E+02** | 5.0681E-03 |
| CLPSO | 1.2578E+02 | 6.5108E+01 |  | 3.7080E+13 | 1.0163E+14 |  | 1.0091E+04 | 2.4766E+03 |
| ALCPSO | 1.4585E+03 | 2.3735E+03 |  | 5.0521E+22 | 2.6375E+23 |  | 2.3990E+04 | 3.9237E+03 |
| SCADE | 2.9076E+10 | 5.7851E+09 |  | 1.3425E+35 | 1.8402E+35 |  | 6.2184E+04 | 8.3371E+03 |
| GAEFA_HK | 1.9963E+08 | 7.2660E+07 |  | 1.1055E+21 | 3.3854E+21 |  | 1.2391E+04 | 4.0116E+03 |
| iAEFA | 1.4994E+03 | 1.6991E+03 |  | 7.9873E+25 | 2.9123E+26 |  | 8.2075E+04 | 1.3302E+04 |
|  | **F4** |  |  | **F5** |  |  | **F6** |  |
|  | Average | Stdv |  | Average | Stdv |  | Average | Stdv |
| DNMRIME | **4.0027E+02** | 1.0115E+00 |  | 6.0891E+02 | 2.2118E+01 |  | 6.3236E+02 | 5.6428E+00 |
| RIME | 4.9862E+02 | 4.0185E+01 |  | 5.7775E+02 | 2.0158E+01 |  | 6.0030E+02 | 2.1910E-01 |
| DE | 4.9656E+02 | 2.3058E+01 |  | 6.0992E+02 | 8.0476E+00 |  | **6.0000E+02** | 0.0000E+00 |
| PSO | 4.7359E+02 | 3.9048E+01 |  | 6.9657E+02 | 2.8144E+01 |  | 6.3848E+02 | 1.1811E+01 |
| WOA | 5.8095E+02 | 4.2943E+01 |  | 7.1427E+02 | 5.9784E+01 |  | 6.6058E+02 | 1.0934E+01 |
| HHO | 5.4090E+02 | 5.0789E+01 |  | 6.6686E+02 | 2.0220E+01 |  | 6.5129E+02 | 3.7883E+00 |
| WSO | 7.7420E+03 | 5.0747E+03 |  | 7.7908E+02 | 4.7656E+01 |  | 6.6556E+02 | 8.9716E+00 |
| AHA | 4.8856E+02 | 3.6318E+01 |  | 6.3903E+02 | 2.8841E+01 |  | 6.0824E+02 | 8.7605E+00 |
| LSHADE | 4.1319E+02 | 2.6794E+01 |  | **5.3184E+02** | 5.6040E+00 |  | 6.0028E+02 | 3.2484E-01 |
| LSHADE_cnEpSi | 4.1015E+02 | 3.1133E+01 |  | 5.3575E+02 | 7.9773E+00 |  | 6.0178E+02 | 1.8090E+00 |
| CLPSO | 4.6471E+02 | 2.0874E+01 |  | 5.5494E+02 | 8.0741E+00 |  | **6.0000E+02** | 8.4444E-14 |
| ALCPSO | 5.1783E+02 | 4.4829E+01 |  | 6.1031E+02 | 3.7756E+01 |  | 6.0421E+02 | 6.1163E+00 |
| SCADE | 2.1999E+03 | 3.8739E+02 |  | 7.8533E+02 | 2.0946E+01 |  | 6.5735E+02 | 6.1959E+00 |
| GAEFA_HK | 5.4761E+02 | 7.8599E+00 |  | 6.1557E+02 | 1.3811E+01 |  | 6.0585E+02 | 1.1257E+00 |
| iAEFA | 5.1432E+02 | 2.6612E+01 |  | 5.3632E+02 | 1.0112E+01 |  | 6.0034E+02 | 5.5972E-01 |
|  | **F7** |  |  | **F8** |  |  | **F9** |  |
|  | Average | Stdv |  | Average | Stdv |  | Average | Stdv |
| DNMRIME | 7.8720E+02 | 1.7629E+01 |  | 9.2659E+02 | 2.2627E+01 |  | 4.1235E+03 | 4.6430E+02 |
| RIME | 8.0957E+02 | 1.6984E+01 |  | 8.8448E+02 | 2.5467E+01 |  | 1.4976E+03 | 6.3683E+02 |
| DE | 8.4343E+02 | 1.0587E+01 |  | 9.1199E+02 | 7.1979E+00 |  | **9.0000E+02** | 2.9856E-14 |
| PSO | 9.2070E+02 | 1.6855E+01 |  | 1.0504E+03 | 2.8169E+01 |  | 6.3034E+03 | 2.2608E+03 |
| WOA | 1.3197E+03 | 1.2374E+02 |  | 1.0888E+03 | 5.6602E+01 |  | 8.4096E+03 | 2.8465E+03 |
| HHO | 1.3056E+03 | 7.2430E+01 |  | 1.0912E+03 | 4.0879E+01 |  | 7.7973E+03 | 8.1346E+02 |
| WSO | 1.4813E+03 | 1.2000E+02 |  | 1.1434E+03 | 6.4378E+01 |  | 7.1208E+03 | 1.9861E+03 |
| AHA | 1.0348E+03 | 1.0984E+02 |  | 9.5938E+02 | 4.1588E+01 |  | 4.5568E+03 | 8.5639E+02 |
| LSHADE | 7.7852E+02 | 1.5446E+01 |  | **8.3187E+02** | 7.8812E+00 |  | 1.0111E+03 | 1.0402E+02 |
| LSHADE_cnEpSi | 7.9150E+02 | 1.7175E+01 |  | 8.4526E+02 | 1.4089E+01 |  | 1.2652E+03 | 3.5974E+02 |
| CLPSO | 7.8465E+02 | 8.1303E+00 |  | 8.4677E+02 | 6.8379E+00 |  | 9.2371E+02 | 2.0244E+01 |
| ALCPSO | 8.5922E+02 | 3.5360E+01 |  | 9.0188E+02 | 3.0679E+01 |  | 1.9768E+03 | 7.0328E+02 |
| SCADE | 1.2803E+03 | 4.3674E+01 |  | 1.1161E+03 | 2.4602E+01 |  | 1.0278E+04 | 1.2952E+03 |
| GAEFA_HK | 8.9844E+02 | 1.8655E+01 |  | 9.1305E+02 | 1.8914E+01 |  | 1.0120E+03 | 4.8228E+01 |
| iAEFA | **7.5294E+02** | 5.2581E+00 |  | 8.4212E+02 | 1.1354E+01 |  | 9.0733E+02 | 3.8896E+01 |

**Appendix Table 3**. Results of DNMRIME and well-known MAs on CEC 2017 (F10-F18).

|  | **F10** |  |  | **F11** |  |  | **F12** |  |
| --- | --- | --- | --- | --- | --- | --- | --- | --- |
|  | Average | Stdv |  | Average | Stdv |  | Average | Stdv |
| DNMRIME | 3.6609E+03 | 5.4253E+02 |  | 1.2074E+03 | 3.4538E+01 |  | 3.4955E+03 | 1.0747E+03 |
| RIME | 3.4485E+03 | 6.2746E+02 |  | 1.2306E+03 | 6.5825E+01 |  | 4.3574E+06 | 3.0369E+06 |
| DE | 5.6186E+03 | 3.2863E+02 |  | 1.1514E+03 | 9.0629E+00 |  | 2.4508E+04 | 7.4877E+04 |
| PSO | 5.5779E+03 | 4.3979E+02 |  | 1.3700E+03 | 4.3715E+01 |  | 7.8806E+07 | 3.3240E+07 |
| WOA | 5.7848E+03 | 9.4841E+02 |  | 1.5163E+03 | 1.1571E+02 |  | 1.4126E+08 | 6.5228E+07 |
| HHO | 4.8386E+03 | 6.8536E+02 |  | 1.3896E+03 | 9.3546E+01 |  | 6.1925E+07 | 4.5477E+07 |
| WSO | 6.7626E+03 | 7.6117E+02 |  | 4.6233E+03 | 3.7076E+03 |  | 3.0139E+09 | 4.3378E+09 |
| AHA | 3.7130E+03 | 5.1662E+02 |  | 1.1976E+03 | 3.1262E+01 |  | 4.4072E+04 | 3.3112E+04 |
| LSHADE | 2.7262E+03 | 3.2465E+02 |  | 1.2918E+03 | 7.9702E+01 |  | 2.7941E+03 | 4.9789E+02 |
| LSHADE_cnEpSi | **2.6061E+03** | 2.6923E+02 |  | 1.3296E+03 | 9.8438E+01 |  | **2.7649E+03** | 5.0007E+02 |
| CLPSO | 2.8829E+03 | 1.9074E+02 |  | **1.1478E+03** | 1.3485E+01 |  | 6.7760E+05 | 1.0838E+06 |
| ALCPSO | 4.0547E+03 | 8.5850E+02 |  | 1.2497E+03 | 5.5720E+01 |  | 7.5188E+03 | 4.8336E+03 |
| SCADE | 7.4266E+03 | 3.4210E+02 |  | 6.7717E+03 | 1.5806E+03 |  | 2.5320E+09 | 7.0872E+08 |
| GAEFA_HK | 4.7602E+03 | 6.6538E+02 |  | 1.5116E+03 | 1.1853E+02 |  | 1.1591E+07 | 6.9403E+06 |
| iAEFA | 3.3180E+03 | 6.0971E+02 |  | 1.4375E+04 | 4.2534E+03 |  | 3.4906E+07 | 1.7475E+07 |
|  | **F13** |  |  | **F14** |  |  | **F15** |  |
|  | Average | Stdv |  | Average | Stdv |  | Average | Stdv |
| DNMRIME | 1.4956E+03 | 1.1960E+02 |  | 1.5145E+03 | 3.1370E+01 |  | **1.5447E+03** | 1.8455E+01 |
| RIME | 3.0455E+03 | 1.9175E+03 |  | 1.5117E+03 | 3.7677E+01 |  | 5.5450E+03 | 4.1695E+03 |
| DE | **1.3690E+03** | 6.7048E+01 |  | **1.4630E+03** | 8.8759E+00 |  | 1.5477E+03 | 1.1218E+01 |
| PSO | 2.5648E+06 | 6.5876E+05 |  | 2.1739E+04 | 1.6449E+04 |  | 3.1542E+05 | 1.1081E+05 |
| WOA | 9.6001E+04 | 8.8193E+04 |  | 2.5273E+05 | 1.6150E+05 |  | 5.8117E+04 | 5.2112E+04 |
| HHO | 2.1837E+05 | 1.1510E+05 |  | 3.0031E+04 | 3.0220E+04 |  | 3.7624E+04 | 1.9703E+04 |
| WSO | 1.4668E+07 | 6.9323E+07 |  | 2.2298E+05 | 7.6490E+05 |  | 9.6201E+03 | 1.3576E+04 |
| AHA | 4.5924E+03 | 3.1659E+03 |  | 1.6815E+03 | 2.7664E+02 |  | 3.0811E+03 | 3.1492E+03 |
| LSHADE | 1.4778E+03 | 1.6617E+02 |  | 1.6227E+03 | 1.7162E+02 |  | 1.6279E+03 | 4.9198E+01 |
| LSHADE_cnEpSi | 3.4931E+03 | 9.8259E+02 |  | 1.7782E+03 | 1.5273E+02 |  | 1.6265E+03 | 7.5297E+01 |
| CLPSO | 1.3975E+03 | 5.9845E+01 |  | 3.6554E+03 | 3.8650E+03 |  | 1.6388E+03 | 1.3408E+02 |
| ALCPSO | 1.7369E+03 | 8.1341E+02 |  | 1.5378E+03 | 7.7595E+01 |  | 1.6272E+03 | 4.6415E+01 |
| SCADE | 1.7245E+08 | 6.1449E+07 |  | 4.5116E+05 | 3.8710E+05 |  | 6.8939E+06 | 3.9245E+06 |
| GAEFA_HK | 1.9749E+04 | 2.3312E+04 |  | 1.7764E+03 | 3.9424E+02 |  | 1.0584E+04 | 7.1645E+03 |
| iAEFA | 6.3747E+04 | 4.4142E+04 |  | 8.4707E+04 | 4.1225E+04 |  | 1.5857E+04 | 9.6372E+03 |
|  | **F16** |  |  | **F17** |  |  | **F18** |  |
|  | Average | Stdv |  | Average | Stdv |  | Average | Stdv |
| DNMRIME | 2.2463E+03 | 2.2972E+02 |  | 2.0199E+03 | 1.7305E+02 |  | 2.0450E+03 | 1.0595E+02 |
| RIME | 2.3214E+03 | 2.6120E+02 |  | 2.0287E+03 | 1.0055E+02 |  | 1.0014E+05 | 6.3445E+04 |
| DE | 1.9792E+03 | 1.6657E+02 |  | 1.9632E+03 | 4.0941E+01 |  | 3.1869E+05 | 1.4185E+05 |
| PSO | 2.6634E+03 | 2.6702E+02 |  | 2.3784E+03 | 2.2277E+02 |  | 7.4672E+04 | 3.5794E+04 |
| WOA | 3.2076E+03 | 4.3315E+02 |  | 2.5775E+03 | 2.6931E+02 |  | 5.2752E+06 | 4.1685E+06 |
| HHO | 3.0506E+03 | 3.8852E+02 |  | 2.5771E+03 | 2.8593E+02 |  | 1.1957E+06 | 1.2830E+06 |
| WSO | 3.1740E+03 | 5.0220E+02 |  | 2.7135E+03 | 3.0601E+02 |  | 2.0587E+05 | 7.5221E+05 |
| AHA | 2.4759E+03 | 2.6859E+02 |  | 2.1197E+03 | 2.2804E+02 |  | 3.1075E+04 | 2.7677E+04 |
| LSHADE | 2.0558E+03 | 1.4211E+02 |  | **1.8388E+03** | 7.9952E+01 |  | **1.9757E+03** | 8.7572E+01 |
| LSHADE_cnEpSi | 1.9927E+03 | 1.6423E+02 |  | 1.8753E+03 | 7.6441E+01 |  | 2.0358E+03 | 8.5955E+01 |
| CLPSO | 2.0441E+03 | 1.3228E+02 |  | 1.9085E+03 | 5.1473E+01 |  | 1.4828E+05 | 1.1873E+05 |
| ALCPSO | 2.4234E+03 | 2.3388E+02 |  | 2.1317E+03 | 1.8951E+02 |  | 2.7340E+05 | 2.2464E+05 |
| SCADE | 3.5188E+03 | 2.1373E+02 |  | 2.6325E+03 | 1.8868E+02 |  | 2.1217E+06 | 1.2458E+06 |
| GAEFA_HK | **1.8631E+03** | 1.5049E+02 |  | 1.9150E+03 | 5.9777E+01 |  | 1.0414E+05 | 4.8048E+04 |
| iAEFA | 2.4875E+03 | 3.0986E+02 |  | 2.3582E+03 | 3.2972E+02 |  | 1.4724E+06 | 6.2284E+05 |

**Appendix Table 4**. Results of DNMRIME and well-known MAs on CEC 2017 (F19-F27).

|  | **F19** |  | |  | **F20** | |  | |  | **F21** | |  |
| --- | --- | --- | --- | --- | --- | --- | --- | --- | --- | --- | --- | --- |
|  | Average | Stdv | |  | Average | | Stdv | |  | Average | | Stdv |
| DNMRIME | **1.9469E+03** | 1.8487E+01 |  | 2.5101E+03 | | 1.0610E+02 | |  | **2.1002E+03** | | 8.2648E-01 | |
| RIME | 6.7589E+03 | 5.3943E+03 |  | 2.2757E+03 | | 1.0748E+02 | |  | 2.2010E+03 | | 3.4900E+01 | |
| DE | 4.5302E+03 | 1.8269E+03 |  | 2.2167E+03 | | 5.7427E+01 | |  | 2.1962E+03 | | 2.2354E+01 | |
| PSO | 3.6915E+05 | 2.0484E+05 |  | 2.6285E+03 | | 1.5592E+02 | |  | 2.1785E+03 | | 3.1210E+01 | |
| WOA | 6.7054E+05 | 8.2174E+05 |  | 2.7199E+03 | | 1.8642E+02 | |  | 2.2658E+03 | | 3.5011E+01 | |
| HHO | 1.3964E+05 | 1.1951E+05 |  | 2.7913E+03 | | 1.8829E+02 | |  | 2.2588E+03 | | 3.1151E+01 | |
| WSO | 7.8285E+03 | 6.8252E+03 |  | 2.7467E+03 | | 2.0731E+02 | |  | 8.8967E+03 | | 3.6181E+03 | |
| AHA | 4.8299E+03 | 4.6647E+03 |  | 2.4574E+03 | | 1.7915E+02 | |  | 2.2057E+03 | | 3.0955E+01 | |
| LSHADE | 2.0623E+03 | 8.8816E+01 |  | 2.1867E+03 | | 9.4017E+01 | |  | 2.1130E+03 | | 3.0224E+01 | |
| LSHADE_cnEpSi | 2.1187E+03 | 9.3997E+01 |  | 2.2010E+03 | | 9.3032E+01 | |  | 2.1280E+03 | | 3.8140E+01 | |
| CLPSO | 1.9662E+03 | 4.9148E+01 |  | 2.2122E+03 | | 6.7757E+01 | |  | 2.1805E+03 | | 1.6697E+01 | |
| ALCPSO | 6.9066E+03 | 5.3297E+03 |  | 2.3704E+03 | | 1.4516E+02 | |  | 2.2053E+03 | | 3.9759E+01 | |
| SCADE | 1.5325E+07 | 1.2227E+07 |  | 2.8775E+03 | | 9.1478E+01 | |  | 4.4501E+03 | | 5.0521E+02 | |
| GAEFA_HK | 9.8371E+03 | 7.6085E+03 |  | **2.1826E+03** | | 4.6312E+01 | |  | 2.2095E+03 | | 7.0895E+00 | |
| iAEFA | 1.7333E+04 | 2.0994E+04 |  | 2.8098E+03 | | 2.1540E+02 | |  | 2.2361E+03 | | 1.8378E+01 | |
|  | **F22** |  | |  | **F23** | |  | |  | **F24** | |  |
|  | Average | Stdv | |  | Average | | Stdv | |  | Average | | Stdv |
| DNMRIME | 2.3290E+03 | 2.4800E+01 | |  | **2.5000E+03** | | 1.6796E-07 | |  | **2.6000E+03** | | 0.0000E+00 |
| RIME | 2.2848E+03 | 2.4095E+01 | |  | 2.8758E+03 | | 1.9881E+01 | |  | 3.2596E+03 | | 2.8350E+02 |
| DE | 2.3084E+03 | 9.5571E+00 | |  | 2.8729E+03 | | 1.0298E+01 | |  | 3.3990E+03 | | 7.6157E+00 |
| PSO | 2.4298E+03 | 3.2434E+01 | |  | 4.6390E+03 | | 5.2286E+02 | |  | 2.6676E+03 | | 3.2680E+00 |
| WOA | 2.4405E+03 | 4.6062E+01 | |  | 3.1679E+03 | | 1.2429E+02 | |  | 2.7196E+03 | | 3.6451E+02 |
| HHO | 2.4158E+03 | 1.8697E+01 | |  | **2.5000E+03** | | 0.0000E+00 | |  | **2.6000E+03** | | 0.0000E+00 |
| WSO | 2.5388E+03 | 3.7741E+01 | |  | 3.7821E+03 | | 2.8717E+02 | |  | 3.0199E+03 | | 8.1779E+02 |
| AHA | 2.3810E+03 | 3.3458E+01 | |  | **2.5000E+03** | | 0.0000E+00 | |  | **2.6000E+03** | | 0.0000E+00 |
| LSHADE | **2.2364E+03** | **1.2043E+01** | |  | 2.8328E+03 | | 2.1023E+01 | |  | 3.2852E+03 | | 2.7373E+02 |
| LSHADE_cnEpSi | 2.2452E+03 | 1.2083E+01 | |  | 2.9023E+03 | | 5.6840E+01 | |  | 2.7999E+03 | | 3.4069E+02 |
| CLPSO | 2.2528E+03 | 7.3357E+00 | |  | 2.8402E+03 | | 1.3804E+01 | |  | 2.6333E+03 | | 5.9014E+01 |
| ALCPSO | 2.3050E+03 | 4.0117E+01 | |  | 3.0305E+03 | | 1.0841E+02 | |  | 3.2433E+03 | | 3.9836E+02 |
| SCADE | 2.5132E+03 | 2.2246E+01 | |  | 3.0196E+03 | | 3.7531E+02 | |  | **2.6000E+03** | | 0.0000E+00 |
| GAEFA_HK | 2.3149E+03 | 1.3929E+01 | |  | 2.9157E+03 | | 2.9601E+01 | |  | 2.6861E+03 | | 1.8026E+01 |
| iAEFA | 2.2477E+03 | 1.1717E+01 | |  | 2.9776E+03 | | 2.6541E+02 | |  | 2.6000E+03 | | 6.8081E-13 |
|  | **F25** |  | |  | **F26** | |  | |  | **F27** | |  |
|  | Average | Stdv | |  | Average | | Stdv | |  | Average | | Stdv |
| DNMRIME | **2.7000E+03** | 2.4836E-12 | |  | **2.8000E+03** | | 1.8882E-13 | |  | 2.9445E+03 | | 1.9470E+02 |
| RIME | 2.9590E+03 | 4.9063E+01 | |  | 5.5004E+03 | | 2.8570E+02 | |  | 3.5915E+03 | | 6.8148E+01 |
| DE | 2.9141E+03 | 1.4352E+01 | |  | 5.3999E+03 | | 1.0503E+02 | |  | 3.4488E+03 | | 2.6533E+01 |
| PSO | 2.9735E+03 | 7.7985E+01 | |  | 3.3803E+03 | | 8.1319E+01 | |  | 4.9219E+03 | | 6.3036E+02 |
| WOA | 2.7288E+03 | 1.0963E+02 | |  | 3.6616E+03 | | 2.0073E+03 | |  | 3.9780E+03 | | 2.0925E+02 |
| HHO | **2.7000E+03** | 0.0000E+00 | |  | **2.8000E+03** | | 0.0000E+00 | |  | **2.9000E+03** | | 0.0000E+00 |
| WSO | 3.1378E+03 | 4.0329E+02 | |  | 3.7201E+03 | | 1.8603E+03 | |  | 4.2076E+03 | | 4.2363E+02 |
| AHA | **2.7000E+03** | 0.0000E+00 | |  | **2.8000E+03** | | 0.0000E+00 | |  | **2.9000E+03** | | 0.0000E+00 |
| LSHADE | 2.9269E+03 | 3.7351E+01 | |  | 4.5591E+03 | | 8.2967E+02 | |  | 3.5488E+03 | | 7.8180E+01 |
| LSHADE_cnEpSi | 2.9910E+03 | 5.9751E+01 | |  | 3.6025E+03 | | 1.0725E+03 | |  | 3.7247E+03 | | 1.3938E+02 |
| CLPSO | 2.9177E+03 | 1.8478E+01 | |  | 3.7532E+03 | | 6.9364E+02 | |  | 3.5065E+03 | | 2.7994E+01 |
| ALCPSO | 2.9756E+03 | 6.0425E+01 | |  | 5.4894E+03 | | 1.0899E+03 | |  | 3.8593E+03 | | 1.7176E+02 |
| SCADE | **2.7000E+03** | 0.0000E+00 | |  | **2.8000E+03** | | 0.0000E+00 | |  | 3.9062E+03 | | 4.0996E+02 |
| GAEFA_HK | 3.0243E+03 | 3.5553E+01 | |  | 3.4322E+03 | | 2.0996E+02 | |  | 3.4908E+03 | | 4.5164E+01 |
| iAEFA | 3.0008E+03 | 3.0703E+01 | |  | 2.8000E+03 | | 9.6282E-13 | |  | 3.9488E+03 | | 2.1789E+02 |

**Appendix Table 5**. Results of DNMRIME and well-known MAs on CEC 2017 (F28-F30).

|  | **F28** |  |  | **F29** |  |  | **F30** |  |
| --- | --- | --- | --- | --- | --- | --- | --- | --- |
|  | Average | Stdv |  | Average | Stdv |  | Average | Stdv |
| DNMRIME | **3.0000E+03** | 1.4553E-12 |  | 3.1000E+03 | 3.5394E-06 |  | 3.3157E+03 | 3.0063E+02 |
| RIME | 3.3662E+03 | 3.5298E+02 |  | 3.5564E+03 | 1.8588E+02 |  | 3.0108E+04 | 1.8136E+04 |
| DE | 4.1626E+03 | 8.2683E+02 |  | 3.4738E+03 | 8.0450E+01 |  | 6.3858E+04 | 2.2807E+04 |
| PSO | 3.2974E+03 | 3.6251E+01 |  | 3.9747E+03 | 2.0239E+02 |  | 2.4494E+06 | 1.2386E+06 |
| WOA | 3.1871E+03 | 4.2325E+02 |  | 4.2812E+03 | 4.6445E+02 |  | 2.3158E+06 | 2.6209E+06 |
| HHO | **3.0000E+03** | 0.0000E+00 |  | **3.1000E+03** | 0.0000E+00 |  | **3.2000E+03** | 0.0000E+00 |
| WSO | 4.4470E+03 | 1.6222E+03 |  | 3.5972E+03 | 6.7693E+02 |  | 1.4906E+07 | 4.1937E+07 |
| AHA | **3.0000E+03** | 0.0000E+00 |  | **3.1000E+03** | 0.0000E+00 |  | **3.2000E+03** | 0.0000E+00 |
| LSHADE | 3.3176E+03 | 3.5126E+02 |  | 3.3898E+03 | 1.0166E+02 |  | 4.1994E+03 | 2.7602E+02 |
| LSHADE_cnEpSi | 3.2560E+03 | 4.6194E+01 |  | 3.4336E+03 | 1.2857E+02 |  | 4.5031E+03 | 4.5140E+02 |
| CLPSO | 3.2766E+03 | 2.1780E+01 |  | 3.3744E+03 | 5.7598E+01 |  | 1.6107E+04 | 7.2907E+03 |
| ALCPSO | 3.3404E+03 | 3.5868E+02 |  | 3.7360E+03 | 1.8789E+02 |  | 4.9027E+04 | 5.1902E+04 |
| SCADE | **3.0000E+03** | 0.0000E+00 |  | **3.1000E+03** | 0.0000E+00 |  | 1.5758E+05 | 3.7647E+05 |
| GAEFA_HK | 3.2645E+03 | 1.0271E+01 |  | 3.4796E+03 | 6.2580E+01 |  | 2.6897E+05 | 1.4457E+05 |
| iAEFA | 3.2868E+03 | 1.1993E+01 |  | 3.6758E+03 | 1.6830E+02 |  | 8.1250E+05 | 3.2092E+05 |

**Appendix Table 6**. IAE values of DNMRIME on SDM.

| **Item** | **Measured data** | | | **Current simulated data** | | **Power simulated data** | |
| --- | --- | --- | --- | --- | --- | --- | --- |
|  | $\text{V(V)}$ | $\text{I(A)}$ | $P\left(W\right)$ | $\text{I}_{\text{S}}\text{(A)}$ | $\text{IAE}_{\text{I}}\text{(A)}$ | $\text{P}_{\text{S}}\text{(W)}$ | $\text{IAE}_{\text{P}}\text{(W)}$ |
| 1 | -0.2057 | 0.7640 | -0.15715480 | 0.764087610 | 0.000087610 | -0.157172821 | 1.802136397E-05 |
| 2 | -0.1291 | 0.7620 | -0.09837420 | 0.762663005 | 0.000663005 | -0.098459794 | 8.559397825E-05 |
| 3 | -0.0588 | 0.7605 | -0.04471740 | 0.761355238 | 0.000855238 | -0.044767688 | 5.028800207E-05 |
| 4 | 0.0057 | 0.7605 | 0.00433485 | 0.760153933 | 0.000346067 | 0.004332877 | 1.972582458E-06 |
| 5 | 0.0646 | 0.7600 | 0.04909600 | 0.759055161 | 0.000944839 | 0.049034963 | 6.103661809E-05 |
| 6 | 0.1185 | 0.7590 | 0.08994150 | 0.758042306 | 0.000957694 | 0.089828013 | 1.134867315E-04 |
| 7 | 0.1678 | 0.7570 | 0.12702460 | 0.757091623 | 0.000091623 | 0.127039974 | 1.537430411E-05 |
| 8 | 0.2132 | 0.7570 | 0.16139240 | 0.756141340 | 0.000858660 | 0.161209334 | 1.830662216E-04 |
| 9 | 0.2545 | 0.7555 | 0.19227475 | 0.755086853 | 0.000413147 | 0.192169604 | 1.051458378E-04 |
| 10 | 0.2924 | 0.7540 | 0.22046960 | 0.753663861 | 0.000336139 | 0.220371313 | 9.828705260E-05 |
| 11 | 0.3269 | 0.7505 | 0.24533845 | 0.751390947 | 0.000890947 | 0.245629701 | 2.912506843E-04 |
| 12 | 0.3585 | 0.7465 | 0.26762025 | 0.747353825 | 0.000853825 | 0.267926346 | 3.060962141E-04 |
| 13 | 0.3873 | 0.7385 | 0.28602105 | 0.740117182 | 0.001617182 | 0.286647385 | 6.263345823E-04 |
| 14 | 0.4137 | 0.7280 | 0.30117360 | 0.727382167 | 0.000617833 | 0.300918002 | 2.555976297E-04 |
| 15 | 0.4373 | 0.7065 | 0.30895245 | 0.706972574 | 0.000472574 | 0.309159107 | 2.066567042E-04 |
| 16 | 0.4590 | 0.6755 | 0.31005450 | 0.675280061 | 0.000219939 | 0.309953548 | 1.009518912E-04 |
| 17 | 0.4784 | 0.6320 | 0.30234880 | 0.630758183 | 0.001241817 | 0.301754715 | 5.940854809E-04 |
| 18 | 0.4960 | 0.5730 | 0.28420800 | 0.571928286 | 0.001071714 | 0.283676430 | 5.315701574E-04 |
| 19 | 0.5119 | 0.4990 | 0.25543810 | 0.499606979 | 0.000606979 | 0.255748813 | 3.107126362E-04 |
| 20 | 0.5265 | 0.4130 | 0.21744450 | 0.413648797 | 0.000648797 | 0.217786091 | 3.415914279E-04 |
| 21 | 0.5398 | 0.3165 | 0.17084670 | 0.317510157 | 0.001010157 | 0.171391983 | 5.452828708E-04 |
| 22 | 0.5521 | 0.2120 | 0.11704520 | 0.212155020 | 0.000155020 | 0.117130786 | 8.558640780E-05 |
| 23 | 0.5633 | 0.1035 | 0.05830155 | 0.102251404 | 0.001248596 | 0.057598216 | 7.033342632E-04 |
| 24 | 0.5736 | -0.0100 | -0.00573600 | -0.008717474 | 0.001282526 | -0.005000343 | 7.356567305E-04 |
| 25 | 0.5833 | -0.1230 | -0.07174590 | -0.125507397 | 0.002507397 | -0.073208464 | 1.462564429E-03 |
| 26 | 0.5900 | -0.2100 | -0.12390000 | -0.208472384 | 0.001527616 | -0.122998707 | 9.012932265E-04 |
| Sum |  |  |  |  | **0.021526941** |  | **8.730838028E-03** |

**Appendix Table 7**. IAE values of DNMRIME on DDM.

| **Item** | **Measured data** | | | **Current simulated data** | | **Power simulated data** | |
| --- | --- | --- | --- | --- | --- | --- | --- |
|  | $\text{V(V)}$ | $\text{I(A)}$ | $P\left(W\right)$ | $\text{I}_{\text{S}}\text{(A)}$ | $\text{IAE}_{\text{I}}\text{(A)}$ | $\text{P}_{\text{S}}\text{(W)}$ | $\text{IAE}_{\text{P}}\text{(W)}$ |
| 1 | -0.2057 | 0.7640 | -0.15715480 | 0.763983391 | 0.000016609 | -0.157151383 | 3.416548116E-06 |
| 2 | -0.1291 | 0.7620 | -0.09837420 | 0.762604088 | 0.000604088 | -0.098452188 | 7.798775483E-05 |
| 3 | -0.0588 | 0.7605 | -0.04471740 | 0.761337703 | 0.000837703 | -0.044766657 | 4.925691333E-05 |
| 4 | 0.0057 | 0.7605 | 0.00433485 | 0.760173803 | 0.000326197 | 0.004332991 | 1.859320558E-06 |
| 5 | 0.0646 | 0.7600 | 0.04909600 | 0.759107706 | 0.000892294 | 0.049038358 | 5.764222462E-05 |
| 6 | 0.1185 | 0.7590 | 0.08994150 | 0.758121453 | 0.000878547 | 0.089837392 | 1.041078153E-04 |
| 7 | 0.1678 | 0.7570 | 0.12702460 | 0.757188652 | 0.000188652 | 0.127056256 | 3.165580838E-05 |
| 8 | 0.2132 | 0.7570 | 0.16139240 | 0.756243646 | 0.000756354 | 0.161231145 | 1.612545832E-04 |
| 9 | 0.2545 | 0.7555 | 0.19227475 | 0.755177337 | 0.000322663 | 0.192192632 | 8.211781425E-05 |
| 10 | 0.2924 | 0.7540 | 0.22046960 | 0.753722378 | 0.000277622 | 0.220388423 | 8.117680186E-05 |
| 11 | 0.3269 | 0.7505 | 0.24533845 | 0.751399140 | 0.000899140 | 0.245632379 | 2.939290158E-04 |
| 12 | 0.3585 | 0.7465 | 0.26762025 | 0.747301427 | 0.000801427 | 0.267907562 | 2.873116056E-04 |
| 13 | 0.3873 | 0.7385 | 0.28602105 | 0.740010622 | 0.001510622 | 0.286606114 | 5.850640818E-04 |
| 14 | 0.4137 | 0.7280 | 0.30117360 | 0.727246899 | 0.000753101 | 0.300862042 | 3.115579609E-04 |
| 15 | 0.4373 | 0.7065 | 0.30895245 | 0.706850241 | 0.000350241 | 0.309105611 | 1.531605842E-04 |
| 16 | 0.4590 | 0.6755 | 0.31005450 | 0.675210497 | 0.000289503 | 0.309921618 | 1.328816497E-04 |
| 17 | 0.4784 | 0.6320 | 0.30234880 | 0.630760733 | 0.001239267 | 0.301755935 | 5.928651203E-04 |
| 18 | 0.4960 | 0.5730 | 0.28420800 | 0.571994732 | 0.001005268 | 0.283709387 | 4.986131621E-04 |
| 19 | 0.5119 | 0.4990 | 0.25543810 | 0.499706151 | 0.000706151 | 0.255799579 | 3.614788345E-04 |
| 20 | 0.5265 | 0.4130 | 0.21744450 | 0.413733695 | 0.000733695 | 0.217830790 | 3.862902510E-04 |
| 21 | 0.5398 | 0.3165 | 0.17084670 | 0.317546223 | 0.001046223 | 0.171411451 | 5.647513344E-04 |
| 22 | 0.5521 | 0.2120 | 0.11704520 | 0.212123002 | 0.000123002 | 0.117113109 | 6.790916947E-05 |
| 23 | 0.5633 | 0.1035 | 0.05830155 | 0.102163270 | 0.001336730 | 0.057548570 | 7.529802328E-04 |
| 24 | 0.5736 | -0.0100 | -0.00573600 | -0.008791756 | 0.001208244 | -0.005042951 | 6.930487615E-04 |
| 25 | 0.5833 | -0.1230 | -0.07174590 | -0.125543436 | 0.002543436 | -0.073229486 | 1.483586189E-03 |
| 26 | 0.5900 | -0.2100 | -0.12390000 | -0.208371561 | 0.001628439 | -0.122939221 | 9.607791518E-04 |
| Sum |  |  |  |  | **0.021275220** |  | **8.776682689E-03** |

**Appendix Table 8**. IAE values of DNMRIME on TDM.

| **Item** | **Measured data** | | | **Current simulated data** | | **Power simulated data** | |
| --- | --- | --- | --- | --- | --- | --- | --- |
|  | $\text{V(V)}$ | $\text{I(A)}$ | $P\left(W\right)$ | $\text{I}_{\text{S}}\text{(A)}$ | $\text{IAE}_{\text{I}}\text{(A)}$ | $\text{P}_{\text{S}}\text{(W)}$ | $\text{IAE}_{\text{P}}\text{(W)}$ |
| 1 | -0.2057 | 0.7640 | -0.15715480 | 0.763987474 | 0.000012526 | -0.157152223 | 2.576518332E-06 |
| 2 | -0.1291 | 0.7620 | -0.09837420 | 0.762606362 | 0.000606362 | -0.098452481 | 7.828139681E-05 |
| 3 | -0.0588 | 0.7605 | -0.04471740 | 0.761338325 | 0.000838325 | -0.044766694 | 4.929350432E-05 |
| 4 | 0.0057 | 0.7605 | 0.00433485 | 0.760172934 | 0.000327066 | 0.004332986 | 1.864278598E-06 |
| 5 | 0.0646 | 0.7600 | 0.04909600 | 0.759105536 | 0.000894464 | 0.049038218 | 5.778238238E-05 |
| 6 | 0.1185 | 0.7590 | 0.08994150 | 0.758118234 | 0.000881766 | 0.089837011 | 1.044892700E-04 |
| 7 | 0.1678 | 0.7570 | 0.12702460 | 0.757184748 | 0.000184748 | 0.127055601 | 3.100067407E-05 |
| 8 | 0.2132 | 0.7570 | 0.16139240 | 0.756239589 | 0.000760411 | 0.161230280 | 1.621196684E-04 |
| 9 | 0.2545 | 0.7555 | 0.19227475 | 0.755173849 | 0.000326151 | 0.192191744 | 8.300555529E-05 |
| 10 | 0.2924 | 0.7540 | 0.22046960 | 0.753720309 | 0.000279691 | 0.220387818 | 8.178164307E-05 |
| 11 | 0.3269 | 0.7505 | 0.24533845 | 0.751399253 | 0.000899253 | 0.245632416 | 2.939657923E-04 |
| 12 | 0.3585 | 0.7465 | 0.26762025 | 0.747304113 | 0.000804113 | 0.267908524 | 2.882744324E-04 |
| 13 | 0.3873 | 0.7385 | 0.28602105 | 0.740015525 | 0.001515525 | 0.286608013 | 5.869627648E-04 |
| 14 | 0.4137 | 0.7280 | 0.30117360 | 0.727252818 | 0.000747182 | 0.300864491 | 3.091089885E-04 |
| 15 | 0.4373 | 0.7065 | 0.30895245 | 0.706855292 | 0.000355292 | 0.309107819 | 1.553693902E-04 |
| 16 | 0.4590 | 0.6755 | 0.31005450 | 0.675212929 | 0.000287071 | 0.309922734 | 1.317657099E-04 |
| 17 | 0.4784 | 0.6320 | 0.30234880 | 0.630759797 | 0.001240203 | 0.301755487 | 5.933131998E-04 |
| 18 | 0.4960 | 0.5730 | 0.28420800 | 0.571990970 | 0.001009030 | 0.283707521 | 5.004788799E-04 |
| 19 | 0.5119 | 0.4990 | 0.25543810 | 0.499701143 | 0.000701143 | 0.255797015 | 3.589149807E-04 |
| 20 | 0.5265 | 0.4130 | 0.21744450 | 0.413729704 | 0.000729704 | 0.217828689 | 3.841891023E-04 |
| 21 | 0.5398 | 0.3165 | 0.17084670 | 0.317544841 | 0.001044841 | 0.171410705 | 5.640052577E-04 |
| 22 | 0.5521 | 0.2120 | 0.11704520 | 0.212125028 | 0.000125028 | 0.117114228 | 6.902816731E-05 |
| 23 | 0.5633 | 0.1035 | 0.05830155 | 0.102167967 | 0.001332033 | 0.057551216 | 7.503340336E-04 |
| 24 | 0.5736 | -0.0100 | -0.00573600 | -0.008787915 | 0.001212085 | -0.005040748 | 6.952520746E-04 |
| 25 | 0.5833 | -0.1230 | -0.07174590 | -0.125541821 | 0.002541821 | -0.073228544 | 1.482644323E-03 |
| 26 | 0.5900 | -0.2100 | -0.12390000 | -0.208376895 | 0.001623105 | -0.122942368 | 9.576320827E-04 |
| Sum |  |  |  |  | **0.021278941** |  | **8.773434071E-03** |

**Appendix Table 9**. IAE of DNMRIME on PV.

| **Item** | **Measured data** | | | **Current simulated data** | | **Power simulated data** | |
| --- | --- | --- | --- | --- | --- | --- | --- |
|  | $\text{V(V)}$ | $\text{I(A)}$ | $P\left(W\right)$ | $\text{I}_{\text{S}}\text{(A)}$ | $\text{IAE}_{\text{I}}\text{(A)}$ | $\text{P}_{\text{S}}\text{(W)}$ | $\text{IAE}_{\text{P}}\text{(W)}$ |
| 1 | 0.1248 | 1.0315 | 0.12873120 | 1.029119077 | 0.002380923 | 0.128434061 | 2.971392499E-04 |
| 2 | 1.8093 | 1.0300 | 1.86357900 | 1.027381034 | 0.002618966 | 1.858840505 | 4.738494808E-03 |
| 3 | 3.3511 | 1.0260 | 3.43822860 | 1.025741799 | 0.000258201 | 3.437363343 | 8.652565683E-04 |
| 4 | 4.7622 | 1.0220 | 4.86696840 | 1.024107194 | 0.002107194 | 4.877003277 | 1.003487695E-02 |
| 5 | 6.0538 | 1.0180 | 6.16276840 | 1.022291874 | 0.004291874 | 6.188750546 | 2.598214637E-02 |
| 6 | 7.2364 | 1.0155 | 7.34856420 | 1.019930774 | 0.004430774 | 7.380627050 | 3.206284954E-02 |
| 7 | 8.3189 | 1.0140 | 8.43536460 | 1.016363212 | 0.002363212 | 8.455023924 | 1.965932358E-02 |
| 8 | 9.3097 | 1.0100 | 9.40279700 | 1.010496259 | 0.000496259 | 9.407417020 | 4.620020485E-03 |
| 9 | 10.2163 | 1.0035 | 10.25205705 | 1.000629064 | 0.002870936 | 10.222726708 | 2.933034217E-02 |
| 10 | 11.0449 | 0.9880 | 10.91236120 | 0.984548446 | 0.003451554 | 10.874239126 | 3.812207357E-02 |
| 11 | 11.8018 | 0.9630 | 11.36513340 | 0.959521705 | 0.003478295 | 11.324083256 | 4.105014393E-02 |
| 12 | 12.4929 | 0.9255 | 11.56217895 | 0.922838804 | 0.002661196 | 11.528932895 | 3.324605528E-02 |
| 13 | 13.1231 | 0.8725 | 11.44990475 | 0.872599610 | 0.000099610 | 11.451211943 | 1.307192705E-03 |
| 14 | 13.6983 | 0.8075 | 11.06137725 | 0.807274185 | 0.000225815 | 11.058283963 | 3.093286563E-03 |
| 15 | 14.2221 | 0.7265 | 10.33235565 | 0.728336389 | 0.001836389 | 10.358472952 | 2.611730169E-02 |
| 16 | 14.6995 | 0.6345 | 9.32683275 | 0.637137917 | 0.002637917 | 9.365608804 | 3.877605428E-02 |
| 17 | 15.1346 | 0.5345 | 8.08944370 | 0.536212999 | 0.001712999 | 8.115369248 | 2.592554817E-02 |
| 18 | 15.5311 | 0.4275 | 6.63954525 | 0.429511284 | 0.002011284 | 6.670782701 | 3.123745113E-02 |
| 19 | 15.8929 | 0.3185 | 5.06188865 | 0.318774466 | 0.000274466 | 5.066250714 | 4.362063560E-03 |
| 20 | 16.2229 | 0.2085 | 3.38247465 | 0.207389508 | 0.001110492 | 3.364459248 | 1.801540242E-02 |
| 21 | 16.5241 | 0.1010 | 1.66893410 | 0.096167184 | 0.004832816 | 1.589076159 | 7.985794093E-02 |
| 22 | 16.7987 | -0.0080 | -0.13438960 | -0.008325387 | 0.000325387 | -0.139855674 | 5.466073727E-03 |
| 23 | 17.0499 | -0.1110 | -1.89253890 | -0.110936504 | 0.000063496 | -1.891456295 | 1.082604733E-03 |
| 24 | 17.2793 | -0.2090 | -3.61137370 | -0.209247319 | 0.000247319 | -3.615647201 | 4.273501374E-03 |
| 25 | 17.4885 | -0.3030 | -5.29901550 | -0.300863688 | 0.002136312 | -5.261654606 | 3.736089387E-02 |
| Sum |  |  |  |  | **0.048923685** |  | **5.168840376E-01** |

**Appendix Table 10**. DNMRIME extracts SDM and DDM parameters of KC200GT at 25℃ and different irradiance.

| **Parameters** | **Irradiance** | | | | |
| --- | --- | --- | --- | --- | --- |
|  | $200W/\text{m}^{2}$ | $400W/\text{m}^{2}$ | $600W/\text{m}^{2}$ | $800W/\text{m}^{2}$ | $1000W/\text{m}^{2}$ |
|  | **SDM** | | | | |
| $I_{ph}(A)$ | 1.646157299 | 3.287812929 | 4.934139549 | 6.570866698 | 8.21160685 |
| $I_{sd}(A)$ | 5.20095E-10 | 1.54015E-09 | 3.99668E-09 | 1.04E-09 | 4.08154E-09 |
| $R_{s}(\Omega)$ | 0.381266049 | 0.352118461 | 0.3365338 | 0.355890351 | 0.334429867 |
| $R_{sh}(\Omega)$ | 690.0389143 | 755.005233 | 753.6347142 | 769.4328411 | 1177.115659 |
| $n$ | 1.003165164 | 1.056650316 | 1.105825834 | 1.039101898 | 1.106210471 |
| $\text{RMSE}$ | 1.418467E-03 | 1.429533E-03 | 1.310762E-03 | 1.702764E-03 | 4.675463E-03 |
|  | **DDM** | | | | |
| $I_{ph}(A)$ | 1.646020352 | 3.28745798 | 4.934307946 | 6.568723794 | 8.21616225 |
| $I_{sd1}(A)$ | 1.39301E-06 | 9.71313E-10 | 3.86143E-09 | 4.60456E-09 | 3.00722E-05 |
| $I_{sd2}(A)$ | 4.84292E-10 | 1.47073E-09 | 1.20283E-14 | 1.17093E-09 | 2.08533E-09 |
| $R_{s}(\Omega)$ | 0.384261248 | 0.347225828 | 0.337338293 | 0.353252529 | 0.344499702 |
| $R_{sh}(\Omega)$ | 698.8817999 | 779.0851184 | 743.0017315 | 870.9906532 | 827.3789549 |
| $n_{1}$ | 2.825785586 | 1.044235942 | 1.104020484 | 1.531807404 | 3.945333436 |
| $n_{2}$ | 1.000000255 | 1.14319551 | 3.974053613 | 1.044866315 | 1.072951415 |
| $\text{RMSE}$ | 1.411115E-03 | 1.534605E-03 | 1.297668E-03 | 2.244126E-03 | 1.563251E-03 |

**Appendix Table 11**. DNMRIME extracts SDM and DDM parameters of ST40 at 25℃ and different irradiance.

| **Parameters** | **Irradiance** | | | | |
| --- | --- | --- | --- | --- | --- |
|  | $200W/\text{m}^{2}$ | $400W/\text{m}^{2}$ | $600W/\text{m}^{2}$ | $800W/\text{m}^{2}$ | $1000W/\text{m}^{2}$ |
|  | **SDM** | | | | |
| $I_{ph}(A)$ | 0.533137374 | 1.067543086 | 1.604806279 | 2.138019925 | 2.675801507 |
| $I_{sd}(A)$ | 1.42974E-06 | 1.84894E-06 | 1.44242E-06 | 1.15777E-06 | 1.52906E-06 |
| $R_{s}(\Omega)$ | 1.185687668 | 1.080566404 | 1.112554079 | 1.125303993 | 1.113209031 |
| $R_{sh}(\Omega)$ | 344.9832157 | 362.5323498 | 347.7225779 | 332.8274815 | 357.5831607 |
| $n$ | 1.747114238 | 1.778543953 | 0.000674038 | 1.718640012 | 1.750346629 |
| $\text{RMSE}$ | 4.772008E-04 | 6.307248E-04 | 6.740378E-04 | 7.739071E-04 | 7.341002E-04 |
|  | **DDM** | | | | |
| $I_{ph}(A)$ | 0.533357073 | 1.06752122 | 1.604728458 | 2.138017608 | 2.675627528 |
| $I_{sd1}(A)$ | 2.66316E-06 | 2.04167E-06 | 4.32638E-07 | 9.58246E-09 | 7.1269E-07 |
| $I_{sd2}(A)$ | 6.62817E-08 | 7.51933E-07 | 1.60604E-06 | 1.15767E-06 | 1.27213E-06 |
| $R_{s}(\Omega)$ | 1.387276621 | 1.102383462 | 1.124890849 | 1.125284076 | 1.117079619 |
| $R_{sh}(\Omega)$ | 348.0235384 | 364.9260795 | 351.1491024 | 332.8085918 | 361.922282 |
| $n_{1}$ | 1.951517497 | 2.021174434 | 1.650834367 | 3.802359808 | 1.692993384 |
| $n_{2}$ | 1.470694586 | 1.695926092 | 1.909667288 | 1.718629562 | 1.91505611 |
| $\text{RMSE}$ | 4.637593E-04 | 6.605579E-04 | 6.852763E-04 | 7.739278E-04 | 7.501145E-04 |

**Appendix Table 12**. DNMRIME extracts SDM and DDM parameters of SM55 at 25℃ and various irradiance.

| **Parameters** | **Irradiance** | | | | |
| --- | --- | --- | --- | --- | --- |
|  | $200W/\text{m}^{2}$ | $400W/\text{m}^{2}$ | $600W/\text{m}^{2}$ | $800W/\text{m}^{2}$ | $1000W/\text{m}^{2}$ |
|  | **SDM** | | | | |
| $I_{ph}(A)$ | 0.692013672 | 1.382843187 | 2.070899898 | 2.760345479 | 3.450102171 |
| $I_{sd}(A)$ | 1.30922E-07 | 1.00449E-07 | 1.55484E-07 | 1.45887E-07 | 1.71172E-07 |
| $R_{s}(\Omega)$ | 0.312419391 | 0.396620592 | 0.330507196 | 0.336833278 | 0.329143309 |
| $R_{sh}(\Omega)$ | 438.0431039 | 427.0621385 | 450.0004948 | 461.2058367 | 483.9480901 |
| $n$ | 1.370898376 | 1.352011516 | 1.387518033 | 1.382225333 | 1.395761298 |
| $\text{RMSE}$ | 5.205415E-04 | 7.076086E-04 | 8.239509E-04 | 6.695905E-04 | 1.146215E-03 |
|  | **DDM** | | | | |
| $I_{ph}(A)$ | 0.692013614 | 1.382310065 | 2.07100592 | 2.760381957 | 3.45008286 |
| $I_{sd1}(A)$ | 1.30925E-07 | 6.83203E-05 | 1.47565E-07 | 1.43956E-07 | 1.63593E-09 |
| $I_{sd2}(A)$ | 1.04916E-18 | 3.72966E-08 | 5.41701E-09 | 1.72114E-13 | 1.69604E-07 |
| $R_{s}(\Omega)$ | 0.312405724 | 0.466954392 | 0.333689566 | 0.337587603 | 0.329164205 |
| $R_{sh}(\Omega)$ | 438.0433695 | 476.7848354 | 446.4360131 | 459.8743427 | 484.3162976 |
| $n_{1}$ | 1.370900446 | 3.543131224 | 1.383254507 | 1.381147475 | 1.366570619 |
| $n_{2}$ | 3.960308188 | 1.277468917 | 1.741532686 | 3.361804944 | 1.396141423 |
| $\text{RMSE}$ | 5.205415E-04 | 6.152014E-04 | 8.320532E-04 | 6.685795E-04 | 1.146331E-03 |

**Appendix Table 13**. DNMRIME extracts SDM and DDM parameters of KC200GT at different temperatures and $1000W/\text{m}^{2}$ irradiance.

| **Parameters** | **Temperatures** | | |
| --- | --- | --- | --- |
|  | $25^{\circ}C$ | $50^{\circ}C$ | $75^{\circ}C$ |
|  | **SDM** | | |
| $I_{ph}(A)$ | 8.217793408 | 8.295304486 | 8.377663363 |
| $I_{sd}(A)$ | 1.93447E-09 | 1.2595E-07 | 1.63081E-06 |
| $R_{s}(\Omega)$ | 0.346017926 | 0.335655109 | 0.342498047 |
| $R_{sh}(\Omega)$ | 707.8369397 | 953.9296324 | 790.5196089 |
| $n$ | 1.069327463 | 1.117290782 | 1.101479421 |
| $\text{RMSE}$ | 1.886218E-03 | 2.746513E-03 | 4.472930E-03 |
|  | **DDM** | | |
| $I_{ph}(A)$ | 8.215256213 | 8.293958345 | 8.376988886 |
| $I_{sd1}(A)$ | 2.55778E-09 | 1.68516E-07 | 1.14524E-06 |
| $I_{sd2}(A)$ | 8.53733E-12 | 7.16408E-09 | 5.28335E-07 |
| $R_{s}(\Omega)$ | 0.341793788 | 0.336973364 | 0.342008729 |
| $R_{sh}(\Omega)$ | 817.8888855 | 1091.582242 | 839.0883767 |
| $n_{1}$ | 1.083457816 | 1.161288477 | 1.100542564 |
| $n_{2}$ | 1.020787401 | 1.018862534 | 1.109770555 |
| $\text{RMSE}$ | 1.927423E-03 | 2.841653E-03 | 4.487460E-03 |

**Appendix Table 14**. DNMRIME extracts SDM and DDM parameters of ST40 at different temperatures and $1000W/\text{m}^{2}$ irradiance.

| **Parameters** | **Temperatures** | | | | |
| --- | --- | --- | --- | --- | --- |
|  | $25^{\circ}C$ | $40^{\circ}C$ | $50^{\circ}C$ | $70^{\circ}C$ | |
|  | **SDM** | | | | |
| $I_{ph}(A)$ | 2.675799161 | 2.680914012 | 2.691967697 | 2.692329553 | |
| $I_{sd}(A)$ | 1.52904E-06 | 5.66556E-06 | 1.86797E-05 | 8.75223E-05 | |
| $R_{s}(\Omega)$ | 1.113216295 | 1.129305434 | 1.149594168 | 1.125888268 | |
| $R_{sh}(\Omega)$ | 357.6141062 | 364.0825703 | 295.0209312 | 367.7531484 | |
| $n$ | 1.750345687 | 1.722545387 | 1.717568679 | 1.727320703 | |
| $\text{RMSE}$ | 7.341004E-04 | 1.321414E-03 | 1.823260E-03 | 7.777181E-04 | |
|  | **DDM** | | | | |
| $I_{ph}(A)$ | 2.675250079 | 2.680588544 | 2.690187295 | 2.69247273 | |
| $I_{sd1}(A)$ | 2.85783E-06 | 5.04333E-06 | 2.98134E-05 | 2.74144E-07 | |
| $I_{sd2}(A)$ | 9.34944E-07 | 2.56593E-06 | 1.11707E-09 | 8.71268E-05 | |
| $R_{s}(\Omega)$ | 1.120177216 | 1.133776068 | 1.257557043 | 1.126205376 | |
| $R_{sh}(\Omega)$ | 369.8967667 | 372.9231098 | 347.5519727 | 365.0633242 | |
| $n_{1}$ | 2.241076089 | 1.913215583 | 1.843945491 | 3.388189143 | |
| $n_{2}$ | 1.701950337 | 1.657162255 | 1.00068434 | 1.72658218 | |
| $\text{RMSE}$ | 7.810730E-04 | 1.349806E-03 | 1.545617E-03 | 7.784114E-04 |  |

**Appendix Table 15**. DNMRIME extracts SDM and DDM parameters of SM55 at different temperatures and $1000W/\text{m}^{2}$ irradiance.

| **Parameters** | **Temperatures** | | |
| --- | --- | --- | --- |
|  | $25^{\circ}C$ | $40^{\circ}C$ | $60^{\circ}C$ |
|  | **SDM** | | |
| $I_{ph}(A)$ | 3.450106418 | 3.469139389 | 3.494609871 |
| $I_{sd}(A)$ | 1.71125E-07 | 1.145E-06 | 6.90914E-06 |
| $R_{s}(\Omega)$ | 0.329150633 | 0.313100326 | 0.31870747 |
| $R_{sh}(\Omega)$ | 483.7973815 | 533.0332424 | 484.849447 |
| $n$ | 1.39573871 | 1.417831047 | 1.40513617 |
| $\text{RMSE}$ | 1.146216E-03 | 3.788815E-03 | 3.780388E-03 |
|  | **DDM** | | |
| $I_{ph}(A)$ | 3.450700054 | 3.469006578 | 3.494609366 |
| $I_{sd1}(A)$ | 9.64786E-08 | 1.17714E-06 | 3.15026E-13 |
| $I_{sd2}(A)$ | 5.4342E-08 | 4.34731E-08 | 6.90955E-06 |
| $R_{s}(\Omega)$ | 0.333416108 | 0.31126866 | 0.318707936 |
| $R_{sh}(\Omega)$ | 461.4881144 | 551.9030184 | 484.8890028 |
| $n_{1}$ | 1.385829743 | 1.427647505 | 1.793120902 |
| $n_{2}$ | 1.384622064 | 1.35869137 | 1.405142619 |
| $\text{RMSE}$ | 1.298120E-03 | 3.810830E-03 | 3.780389E-03 |
